# Supplementary material for: The Effect of Anti-Tumor Necrosis Factor-Alpha Therapy within 12 Weeks Prior to Surgery on Postoperative Complications in Inflammatory Bowel Disease: A Systematic Review and Meta-Analysis
Source: J Clin Med. 2022 Nov 22;11(23):6884. doi: 10.3390/jcm11236884 (PMC9738467; doi:10.3390/jcm11236884)
Supplement: Supplementary file 1 [file jcm-11-06884-s001.zip › jcm-1950232-supplementary.pdf]

BACK MATTER

Study or Subgroup	anti-TNF-alpha group Events	anti-TNF-alpha group Total	control group Events	control group Total	Weight	Odds Ratio M-H, Random, 95% CI
Bregnbak 2012	4	20	21	51	2.7%	0.36 [0.10, 1.22]
Ferrante 2009	2	22	29	119	1.9%	0.31 [0.07, 1.41]
Gainsbury 2011	5	29	14	52	3.0%	0.57 [0.18, 1.77]
Jouvin 2018	14	55	44	305	5.8%	2.03 [1.02, 4.02]
Kim 2020	8	74	11	54	3.7%	0.47 [0.18, 1.27]
Krane 2013	17	142	42	376	6.6%	1.08 [0.59, 1.97]
Kunitake 2008	6	101	32	312	4.2%	0.55 [0.22, 1.36]
Lau 2015 (1)	11	50	7	73	3.5%	2.66 [0.95, 7.43]
Lau 2015 (2)	2	17	10	77	1.7%	0.89 [0.18, 4.51]
Lightner 2019	33	197	30	167	7.2%	0.92 [0.53, 1.58]
Myrelid 2014	18	111	26	187	6.1%	1.20 [0.62, 2.30]
Nelson 2014	6	24	12	50	3.0%	1.06 [0.34, 3.26]
Regadas 2011	0	28	22	221	0.6%	0.16 [0.01, 2.64]
Rizzo 2011	9	54	8	60	3.5%	1.30 [0.46, 3.65]
Selvasekar 2007	6	23	25	254	3.5%	3.23 [1.17, 8.95]
Syed 2013	54	150	44	175	8.0%	1.67 [1.04, 2.70]
Tang 2020	22	85	43	305	6.8%	2.13 [1.19, 3.81]
Tay 2003	3	22	8	78	2.1%	1.38 [0.33, 5.72]
Ward 2018	35	753	270	5472	9.4%	0.94 [0.65, 1.35]
Yamada 2017 (1)	10	96	16	121	4.6%	0.76 [0.33, 1.77]
Yamada 2017 (2)	9	33	18	129	4.1%	2.31 [0.93, 5.77]
Yu 2019	28	122	126	695	8.1%	1.35 [0.85, 2.14]
**Total (95% CI)**		**2208**		**9333**	**100.0%**	**1.16 [0.92, 1.45]**
Total events	302		858			

Heterogeneity: Tau² = 0.11; Chi² = 37.08, df = 21 (P = 0.02); I² = 43%
Test for overall effect: Z = 1.24 (P = 0.21)

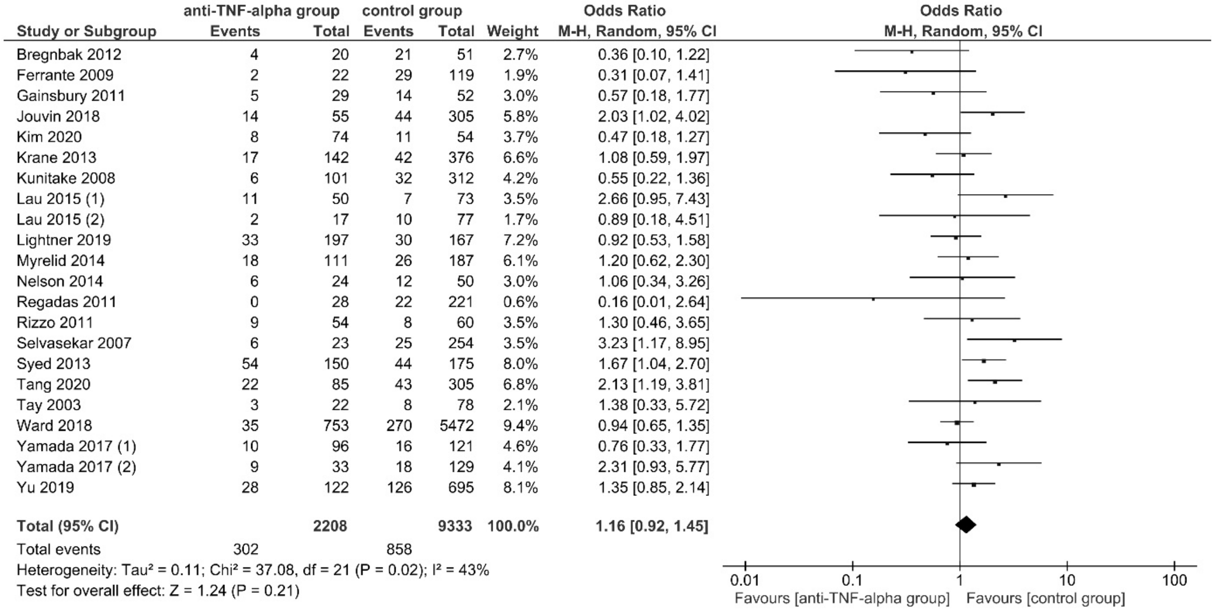

Figure S1: Random-effects model meta-analysis for 30-day overall infectious postoperative complications in the anti-TNF-α (intervention) and control group. Forest plot of all studies included.

Study or Subgroup	anti-TNF-alpha group Events	anti-TNF-alpha group Total	control group Events	control group Total	Weight	Odds Ratio M-H, Fixed, 95% CI
Kotze 2018	7	25	9	25	7.3%	0.69 [0.21, 2.28]
Krane 2013	12	142	32	376	18.0%	0.99 [0.50, 1.99]
Mascarenhas 2012	2	19	3	74	1.2%	2.78 [0.43, 17.99]
Myrelid 2014	12	111	20	187	14.9%	1.01 [0.47, 2.16]
Syed 2013	47	150	47	175	33.4%	1.24 [0.77, 2.01]
Tang 2020	19	85	61	305	23.2%	1.15 [0.64, 2.06]
Tiberi 2020	2	32	4	101	2.0%	1.62 [0.28, 9.27]
**Total (95% CI)**		**564**		**1243**	**100.0%**	**1.13 [0.85, 1.50]**
Total events	101		176			

Heterogeneity: Chi² = 2.08, df = 6 (P = 0.91); I² = 0%
Test for overall effect: Z = 0.84 (P = 0.40)

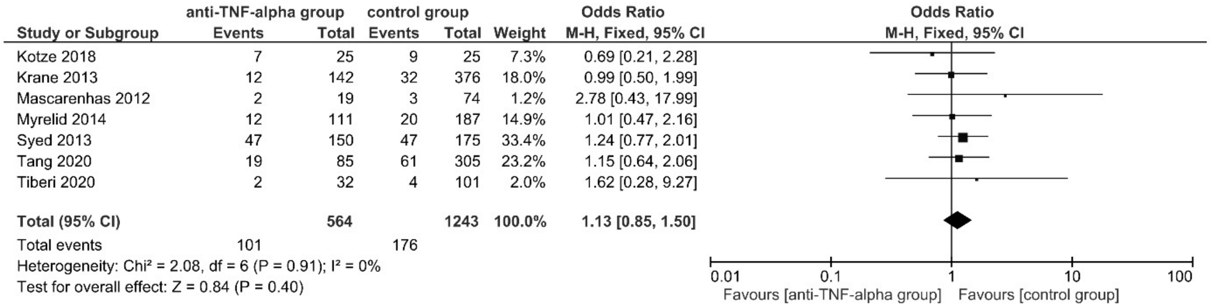

Figure S2: Fixed-effects model meta-analysis for 30-day overall postoperative Clavien-Dindo major complications in the anti-TNF-α (intervention) and control group. Forest plot of all studies included.

Study or Subgroup	anti-TNF-alpha group Events	anti-TNF-alpha group Total	control group Events	control group Total	Weight	Odds Ratio M-H, Fixed, 95% CI
Appau 2008	5	60	10	329	1.7%	2.90 [0.95, 8.81]
Canedo 2010	2	65	9	160	3.0%	0.53 [0.11, 2.53]
Gu 2013 (2)	8	142	20	265	7.8%	0.73 [0.31, 1.71]
Kotze 2017	12	71	7	52	4.0%	1.31 [0.48, 3.59]
Kotze 2018	4	25	4	25	2.0%	1.00 [0.22, 4.54]
Krane 2013	5	142	9	376	2.8%	1.49 [0.49, 4.52]
Myrelid 2014	9	111	13	187	5.3%	1.18 [0.49, 2.86]
Nørgård 2012	43	199	230	997	35.5%	0.92 [0.64, 1.33]
Nørgård 2013	16	213	175	2025	18.2%	0.86 [0.50, 1.46]
Regadas 2011	0	28	9	221	1.3%	0.39 [0.02, 6.93]
Rizzo 2011	3	54	1	60	0.5%	3.47 [0.35, 34.41]
Syed 2013	24	150	23	175	10.5%	1.26 [0.68, 2.34]
Waterman 2012	3	104	12	278	3.8%	0.66 [0.18, 2.38]
Yamada 2017 (1)	6	96	4	121	2.0%	1.95 [0.53, 7.12]
Yamada 2017 (2)	2	33	8	129	1.8%	0.98 [0.20, 4.83]
**Total (95% CI)**		**1493**		**5400**	**100.0%**	**1.02 [0.82, 1.26]**
Total events	142		534			

Heterogeneity: Chi² = 9.53, df = 14 (P = 0.80); I² = 0%
Test for overall effect: Z = 0.15 (P = 0.88)

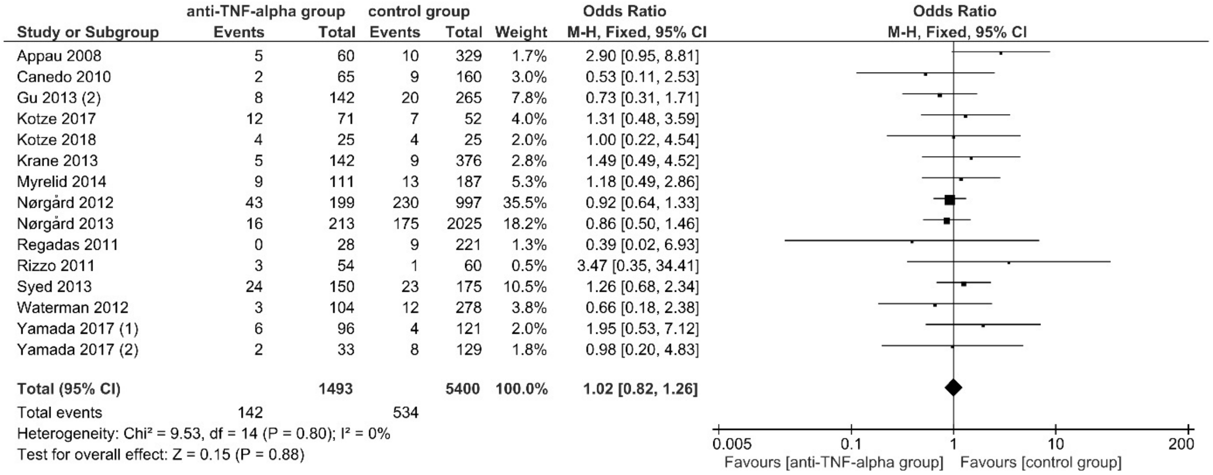

Figure S3: Fixed-effects model meta-analysis for 30-day postoperative reoperation rate in the anti-TNF-α (intervention) and control group. Forest plot of all studies included.

Study or Subgroup	anti-TNF-alpha group Events	Total	control group Events	Total	Weight	Odds Ratio M-H, Fixed, 95% CI
Appau 2008	1	60	0	329	0.8%	16.61 [0.67, 412.69]
Bregnbak 2012	0	20	1	51	4.4%	0.82 [0.03, 21.00]
Ferrante 2009	1	22	5	119	7.8%	1.09 [0.12, 9.77]
Gu 2013 (2)	2	142	8	265	29.0%	0.46 [0.10, 2.19]
Kotze 2017	1	71	2	52	12.0%	0.36 [0.03, 4.05]
Kotze 2018	0	25	1	25	7.7%	0.32 [0.01, 8.25]
Krane 2013	0	142	1	376	4.3%	0.88 [0.04, 21.69]
Waterman 2012	6	104	11	278	29.7%	1.49 [0.54, 4.13]
Yamada 2017 (2)	1	33	2	129	4.2%	1.98 [0.17, 22.58]
**Total (95% CI)**		**619**		**1624**	**100.0%**	**1.02 [0.54, 1.91]**
Total events	12		31			

Heterogeneity: $Chi^2 = 5.95$, df = 8 (P = 0.65); $I^2 = 0\%$
Test for overall effect: Z = 0.05 (P = 0.96)

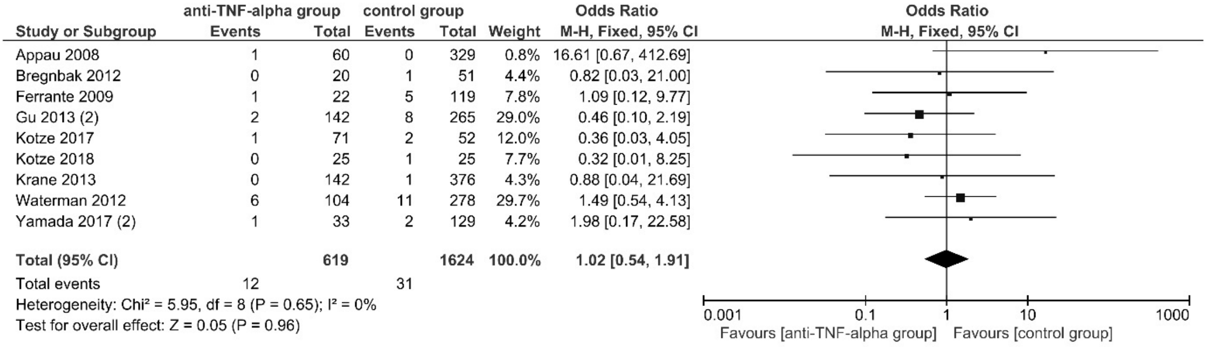

Figure S4: Fixed-effects model meta-analysis for 30-day postoperative mortality in the anti-TNF-α (intervention) and control group. Forest plot of all studies included.

Study or Subgroup	anti-TNF-alpha group Events	Total	control group Events	Total	Weight	Odds Ratio M-H, Random, 95% CI
Ferrante 2009	1	22	23	119	5.0%	0.20 [0.03, 1.55]
Kotze 2017	26	71	11	52	10.4%	2.15 [0.95, 4.90]
Kotze 2018	9	25	6	25	8.3%	1.78 [0.52, 6.09]
Maeda 2015	8	65	27	112	10.2%	0.44 [0.19, 1.04]
Nelson 2014	4	24	8	50	7.9%	1.05 [0.28, 3.90]
Serradori 2013	9	42	15	175	10.0%	2.91 [1.17, 7.21]
Tang 2020	24	85	50	305	11.7%	2.01 [1.14, 3.52]
Uchino 2013	9	79	99	326	10.9%	0.29 [0.14, 0.61]
Uchino 2013 (UC)	1	22	46	174	5.0%	0.13 [0.02, 1.01]
Uchino 2015	5	44	32	137	9.4%	0.42 [0.15, 1.16]
Uchino 2019	16	146	25	155	11.2%	0.64 [0.33, 1.25]
**Total (95% CI)**		**625**		**1630**	**100.0%**	**0.81 [0.45, 1.45]**
Total events	112		342			

Heterogeneity: $Tau^2 = 0.66$; $Chi^2 = 40.98$, df = 10 (P < 0.0001); $I^2 = 76\%$
Test for overall effect: Z = 0.71 (P = 0.48)

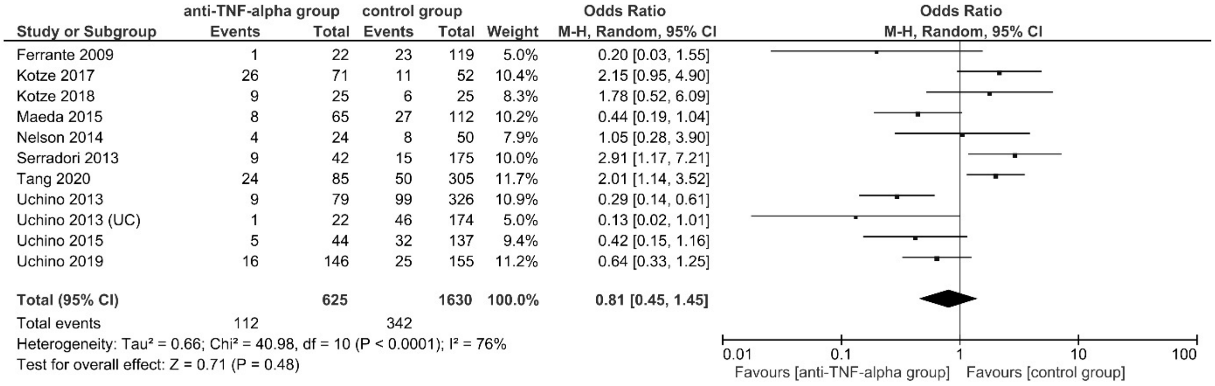

Figure S5: Random-effects model meta-analysis for 30-day overall infectious surgical-site postoperative complications in the anti-TNF-α (intervention) and control group. Forest plot of all studies included.

Study or Subgroup	anti-TNF-alpha group Events	Total	control group Events	Total	Weight	Odds Ratio M-H, Fixed, 95% CI
Appau 2008	6	60	14	329	3.3%	2.50 [0.92, 6.79]
Canedo 2010	2	35	5	101	2.1%	1.16 [0.22, 6.29]
Ferrante 2009	0	22	15	119	4.1%	0.15 [0.01, 2.60]
Gu 2013 (2)	16	142	24	265	12.6%	1.28 [0.65, 2.49]
Kotze 2017	6	71	6	52	5.4%	0.71 [0.21, 2.33]
Kotze 2018	2	25	4	25	3.1%	0.46 [0.08, 2.75]
Krane 2013	3	142	5	376	2.3%	1.60 [0.38, 6.79]
Kunitake 2008	3	101	9	312	3.6%	1.03 [0.27, 3.88]
Myrelid 2014	8	111	15	187	8.8%	0.89 [0.36, 2.17]
Nørgård 2012	1	199	16	997	4.5%	0.31 [0.04, 2.35]
Nørgård 2013	8	213	56	2025	8.7%	1.37 [0.65, 2.92]
Regadas 2011	0	28	3	221	0.7%	1.10 [0.06, 21.75]
Rizzo 2011	4	54	3	60	2.2%	1.52 [0.32, 7.12]
Shwaartz 2016	4	73	11	209	4.6%	1.04 [0.32, 3.38]
Syed 2013	9	150	9	175	6.6%	1.18 [0.45, 3.05]
Tang 2020	5	85	18	305	6.3%	1.00 [0.36, 2.77]
Tay 2003	1	22	3	78	1.1%	1.19 [0.12, 12.05]
Waterman 2012	2	104	15	278	6.8%	0.34 [0.08, 1.53]
Yamada 2017 (1)	2	96	2	121	1.5%	1.27 [0.18, 9.16]
Yamada 2017 (2)	2	33	2	129	0.6%	4.10 [0.56, 30.24]
Yamamoto 2016	7	79	12	152	6.3%	1.13 [0.43, 3.01]
Zittan 2016	2	27	66	562	4.8%	0.60 [0.14, 2.60]
**Total (95% CI)**		**1872**		**7078**	**100.0%**	**1.02 [0.79, 1.32]**
Total events	93		313			

Heterogeneity: $Chi^2 = 13.63$, df = 21 (P = 0.88); $I^2 = 0\%$
Test for overall effect: Z = 0.18 (P = 0.86)

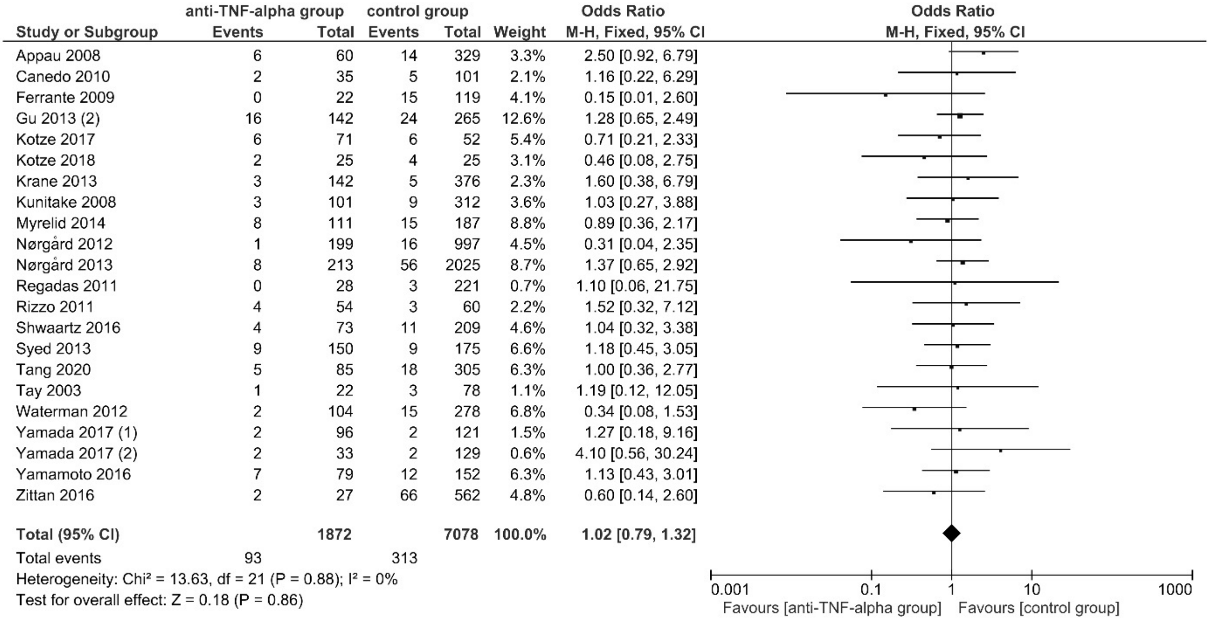

Figure S6: Fixed-effects model meta-analysis for 30-day postoperative anastomotic leakage in the anti-TNF-α (intervention) and control group. Forest plot of all studies included.

Study or Subgroup	anti-TNF-alpha group Events	anti-TNF-alpha group Total	control group Events	control group Total	Weight	Odds Ratio M-H, Fixed, 95% CI
Appau 2008	6	60	14	329	4.8%	2.50 [0.92, 6.79]
Bregnbak 2012	1	20	2	51	1.3%	1.29 [0.11, 15.07]
Canedo 2010	2	65	8	160	5.6%	0.60 [0.12, 2.92]
Ferrante 2009	0	22	9	119	3.7%	0.26 [0.01, 4.60]
Gainsbury 2011	4	29	7	52	5.4%	1.03 [0.27, 3.86]
Gu 2013 (2)	3	142	12	265	10.2%	0.46 [0.13, 1.64]
Kim 2020	2	74	0	54	0.7%	3.76 [0.18, 79.89]
Kotze 2017	10	71	2	52	2.5%	4.10 [0.86, 19.57]
Kotze 2018	3	25	2	25	2.2%	1.57 [0.24, 10.30]
Krane 2013	8	142	20	376	12.8%	1.06 [0.46, 2.47]
Nelson 2014	2	24	2	50	1.5%	2.18 [0.29, 16.51]
Regadas 2011	0	28	5	221	1.5%	0.69 [0.04, 12.82]
Shwaartz 2016	4	73	4	209	2.4%	2.97 [0.72, 12.20]
Syed 2013	21	150	18	175	17.7%	1.42 [0.73, 2.78]
Tang 2020	2	85	6	305	3.2%	1.20 [0.24, 6.06]
Tay 2003	2	22	4	78	2.0%	1.85 [0.32, 10.84]
Yamada 2017 (1)	6	96	8	121	8.2%	0.94 [0.32, 2.81]
Yamada 2017 (2)	2	33	10	129	4.7%	0.77 [0.16, 3.69]
Zittan 2016	3	27	96	562	9.7%	0.61 [0.18, 2.06]
**Total (95% CI)**		**1188**		**3333**	**100.0%**	**1.19 [0.89, 1.60]**
Total events	81		229			

Heterogeneity: Chi² = 13.46, df = 18 (P = 0.76); I² = 0%
Test for overall effect: Z = 1.18 (P = 0.24)

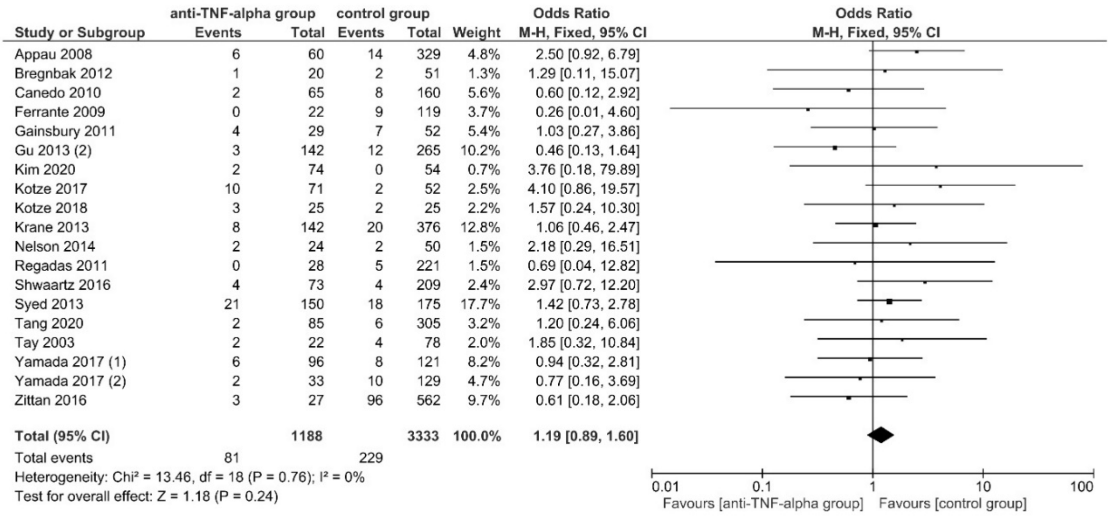

Figure S7: Fixed-effects model meta-analysis for 30-day postoperative intra-abdominal abscesses in the anti-TNF-α (intervention) and control group. Forest plot of all studies included.

Study or Subgroup	anti-TNF-alpha group Events	anti-TNF-alpha group Total	control group Events	control group Total	Weight	Odds Ratio M-H, Fixed, 95% CI
Bregnbak 2012	6	20	10	51	8.0%	1.76 [0.54, 5.72]
Gainsbury 2011	12	29	16	52	13.5%	1.59 [0.62, 4.09]
Nelson 2014	5	24	20	50	20.7%	0.39 [0.13, 1.23]
Yamada 2017 (1)	15	96	25	121	37.6%	0.71 [0.35, 1.44]
Yamada 2017 (2)	6	33	30	129	20.2%	0.73 [0.28, 1.94]
**Total (95% CI)**		**202**		**403**	**100.0%**	**0.85 [0.56, 1.29]**
Total events	44		101			

Heterogeneity: Chi² = 5.22, df = 4 (P = 0.27); I² = 23%
Test for overall effect: Z = 0.76 (P = 0.45)

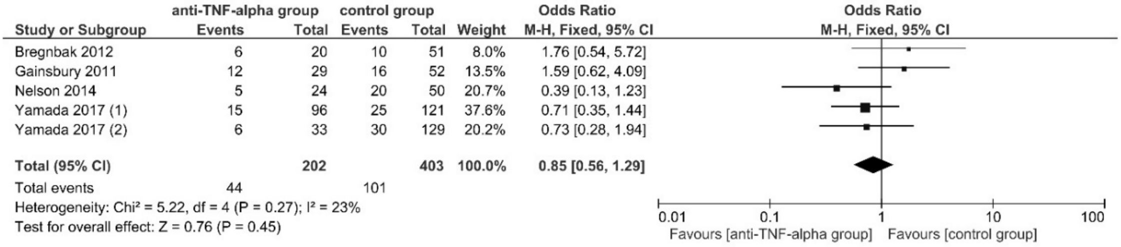

Figure S8: Fixed-effects model meta-analysis for 30-day overall non-infectious postoperative complications in the anti-TNF-α (intervention) and control group. Forest plot of all studies included.

Study or Subgroup	anti-TNF-alpha group Events	anti-TNF-alpha group Total	control group Events	control group Total	Weight	Odds Ratio M-H, Fixed, 95% CI
Kotze 2018	5	25	8	25	7.6%	0.53 [0.15, 1.93]
Krane 2013	24	142	60	376	32.4%	1.07 [0.64, 1.80]
Mascarenhas 2012	2	19	3	74	1.3%	2.78 [0.43, 17.99]
Myrelid 2014	43	111	66	187	35.7%	1.16 [0.71, 1.88]
Tang 2020	33	85	73	305	23.1%	2.02 [1.21, 3.36]
**Total (95% CI)**		**382**		**967**	**100.0%**	**1.30 [0.98, 1.72]**
Total events	107		210			

Heterogeneity: Chi² = 6.09, df = 4 (P = 0.19); I² = 34%
Test for overall effect: Z = 1.85 (P = 0.06)

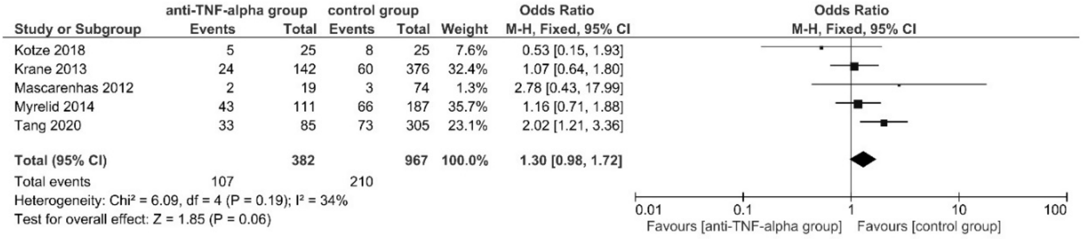

Figure S9: Fixed-effects model meta-analysis for 30-day overall postoperative Clavien-Dindo minor complications in the anti-TNF-α (intervention) and control group. Forest plot of all studies included.

Study or Subgroup	anti-TNF-alpha group Events	anti-TNF-alpha group Total	control group Events	control group Total	Weight	Odds Ratio M-H, Random, 95% CI
El-Hussuna 2018	1	18	2	28	5.6%	0.76 [0.06, 9.10]
Maeda 2015	8	65	27	112	12.7%	0.44 [0.19, 1.04]
Nelson 2014	2	24	6	50	8.5%	0.67 [0.12, 3.58]
Regadas 2011	0	28	10	221	4.6%	0.35 [0.02, 6.19]
Tang 2020	15	85	24	305	13.4%	2.51 [1.25, 5.03]
Uchino 2013	4	79	70	326	11.7%	0.20 [0.07, 0.55]
Uchino 2013 (UC)	1	22	31	174	7.1%	0.22 [0.03, 1.69]
Uchino 2015	2	44	22	137	9.4%	0.25 [0.06, 1.10]
Uchino 2019	9	146	11	155	12.4%	0.86 [0.35, 2.14]
Yamada 2017 (1)	1	96	3	121	6.2%	0.41 [0.04, 4.04]
Yamada 2017 (2)	4	33	2	129	8.3%	8.76 [1.53, 50.13]
**Total (95% CI)**		**640**		**1758**	**100.0%**	**0.67 [0.32, 1.40]**
Total events	47		208			

Heterogeneity: Tau² = 0.93; Chi² = 32.21, df = 10 (P = 0.0004); I² = 69%
Test for overall effect: Z = 1.06 (P = 0.29)

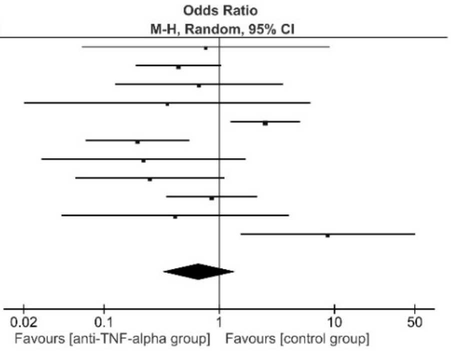

Figure S10: Random-effects model meta-analysis for 30-day postoperative superficial surgical-site infections in the anti-TNF-α (intervention) and control group. Forest plot of all studies included.

Study or Subgroup	anti-TNF-alpha group Events	Total	control group Events	Total	Weight	Odds Ratio M-H, Fixed, 95% CI
Tang 2020	9	85	26	305	24.3%	1.27 [0.57, 2.83]
Uchino 2013	5	79	29	326	25.4%	0.69 [0.26, 1.85]
Uchino 2013 (UC)	0	22	15	174	8.4%	0.23 [0.01, 3.96]
Uchino 2015	3	44	10	137	10.9%	0.93 [0.24, 3.54]
Uchino 2019	7	146	14	155	31.0%	0.51 [0.20, 1.29]
**Total (95% CI)**		**376**		**1097**	**100.0%**	**0.76 [0.48, 1.22]**
Total events	24		94			

Heterogeneity: Chi² = 3.10, df = 4 (P = 0.54); I² = 0%
Test for overall effect: Z = 1.13 (P = 0.26)

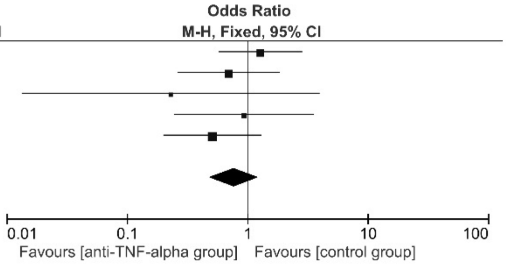

Figure S11: Fixed-effects model meta-analysis for 30-day postoperative deep or organ space surgical-site infections in the anti-TNF-α (intervention) and control group. Forest plot of all studies included.

Study or Subgroup	anti-TNF-alpha group Events	Total	control group Events	Total	Weight	Odds Ratio M-H, Fixed, 95% CI
Regadas 2011	0	28	2	221	9.9%	1.54 [0.07, 32.90]
Syed 2013	2	150	4	175	63.3%	0.58 [0.10, 3.20]
Tay 2003	0	22	3	78	26.8%	0.48 [0.02, 9.63]
**Total (95% CI)**		**200**		**474**	**100.0%**	**0.65 [0.17, 2.47]**
Total events	2		9			

Heterogeneity: Chi² = 0.36, df = 2 (P = 0.83); I² = 0%
Test for overall effect: Z = 0.64 (P = 0.52)

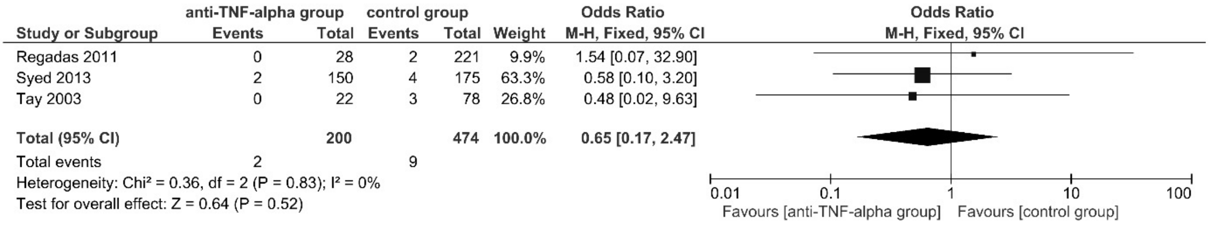

Figure S12: Fixed-effects model meta-analysis for 30-day postoperative fistula formations in the anti-TNF-α (intervention) and control group. Forest plot of all studies included.

Study or Subgroup	anti-TNF-alpha group Events	Total	control group Events	Total	Weight	Odds Ratio M-H, Fixed, 95% CI
Bregnbak 2012	6	20	7	51	3.7%	2.69 [0.78, 9.36]
Gu 2013 (2)	17	142	34	265	27.9%	0.92 [0.50, 1.72]
Kim 2020	16	74	8	54	9.7%	1.59 [0.62, 4.03]
Krane 2013	8	142	9	376	6.2%	2.43 [0.92, 6.44]
Mascarenhas 2012	1	19	0	74	0.3%	12.08 [0.47, 308.74]
Nasir 2010	13	119	38	251	29.1%	0.69 [0.35, 1.35]
Regadas 2011	2	28	41	221	11.4%	0.34 [0.08, 1.48]
Rizzo 2011	2	54	0	60	0.6%	5.76 [0.27, 122.74]
Tang 2020	3	85	4	305	2.2%	2.75 [0.60, 12.55]
Yamada 2017 (1)	4	96	5	121	5.7%	1.01 [0.26, 3.86]
Yamada 2017 (2)	1	33	6	129	3.2%	0.64 [0.07, 5.51]
**Total (95% CI)**		**812**		**1907**	**100.0%**	**1.11 [0.81, 1.51]**
Total events	73		152			

Heterogeneity: Chi² = 14.65, df = 10 (P = 0.15); I² = 32%
Test for overall effect: Z = 0.64 (P = 0.52)

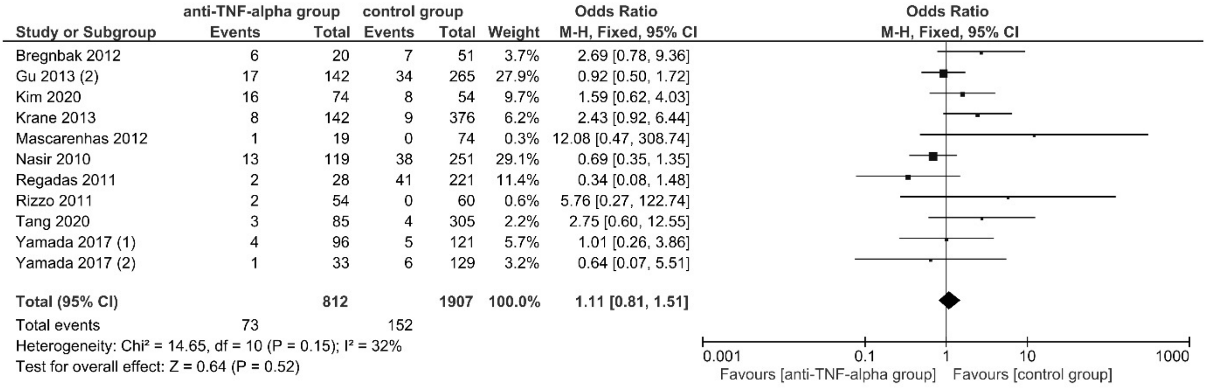

Figure S13: Fixed-effects model meta-analysis for 30-day postoperative ileus in the anti-TNF-α (intervention) and control group. Forest plot of all studies included.

Study or Subgroup	anti-TNF-alpha group Events	Total	control group Events	Total	Weight	Odds Ratio M-H, Fixed, 95% CI
Gu 2013 (1)	1	25	6	156	5.4%	1.04 [0.12, 9.04]
Gu 2013 (2)	6	142	12	265	27.1%	0.93 [0.34, 2.53]
Kotze 2017	3	71	5	52	18.7%	0.41 [0.09, 1.82]
Kotze 2018	0	25	3	25	11.6%	0.13 [0.01, 2.58]
Krane 2013	3	142	9	376	16.3%	0.88 [0.23, 3.30]
Regadas 2011	1	28	9	221	6.6%	0.87 [0.11, 7.16]
Tang 2020	3	85	10	305	14.2%	1.08 [0.29, 4.01]
**Total (95% CI)**		**518**		**1400**	**100.0%**	**0.76 [0.43, 1.32]**
Total events	17		54			

Heterogeneity: Chi² = 2.59, df = 6 (P = 0.86); I² = 0%
Test for overall effect: Z = 0.98 (P = 0.33)

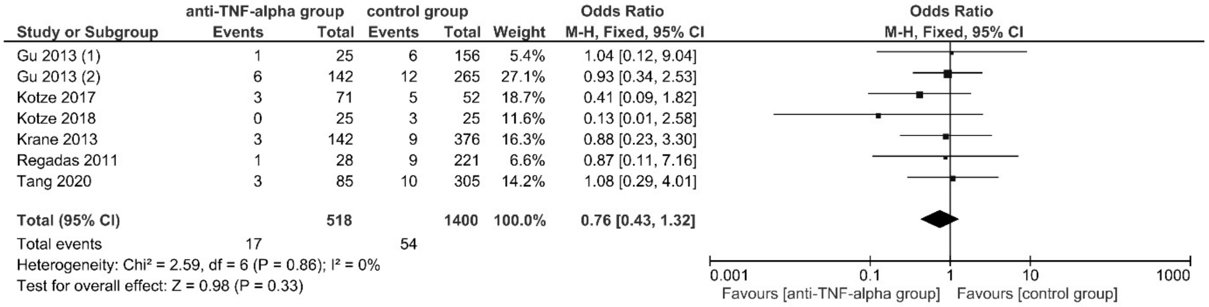

Figure S14: Fixed-effects model meta-analysis for 30-day postoperative small bowel obstructions in the anti-TNF-α (intervention) and control group. Forest plot of all studies included.

Study or Subgroup	anti-TNF-alpha group Events	Total	control group Events	Total	Weight	Odds Ratio M-H, Fixed, 95% CI
Gu 2013 (1)	0	25	5	156	4.9%	0.54 [0.03, 10.07]
Gu 2013 (2)	3	142	9	265	19.8%	0.61 [0.16, 2.30]
Kunitake 2008	1	101	2	312	3.1%	1.55 [0.14, 17.28]
Rizzo 2011	3	54	1	60	2.9%	3.47 [0.35, 34.41]
Syed 2013	7	150	12	175	34.0%	0.66 [0.25, 1.73]
Tang 2020	4	85	14	305	18.7%	1.03 [0.33, 3.20]
Yamada 2017 (1)	2	96	5	121	14.0%	0.49 [0.09, 2.60]
Yamada 2017 (2)	1	33	2	129	2.5%	1.98 [0.17, 22.58]
**Total (95% CI)**		**686**		**1523**	**100.0%**	**0.83 [0.49, 1.41]**
Total events	21		50			

Heterogeneity: Chi² = 3.24, df = 7 (P = 0.86); I² = 0%
Test for overall effect: Z = 0.67 (P = 0.50)

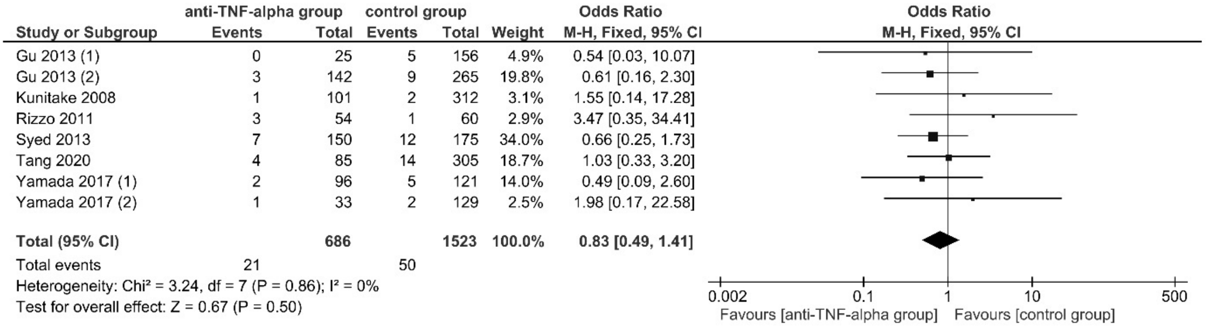

Figure S15: Fixed-effects model meta-analysis for 30-day postoperative hemorrhage in the anti-TNF-α (intervention) and control group. Forest plot of all studies included.

Study or Subgroup	anti-TNF-alpha group Events	Total	control group Events	Total	Weight	Odds Ratio M-H, Fixed, 95% CI
Ferrante 2009	1	22	10	119	40.6%	0.52 [0.06, 4.27]
Nelson 2014	2	24	4	50	32.4%	1.05 [0.18, 6.15]
Shwaartz 2016	3	73	4	209	27.0%	2.20 [0.48, 10.06]
**Total (95% CI)**		**119**		**378**	**100.0%**	**1.14 [0.43, 3.03]**
Total events	6		18			

Heterogeneity: Chi² = 1.26, df = 2 (P = 0.53); I² = 0%
Test for overall effect: Z = 0.27 (P = 0.79)

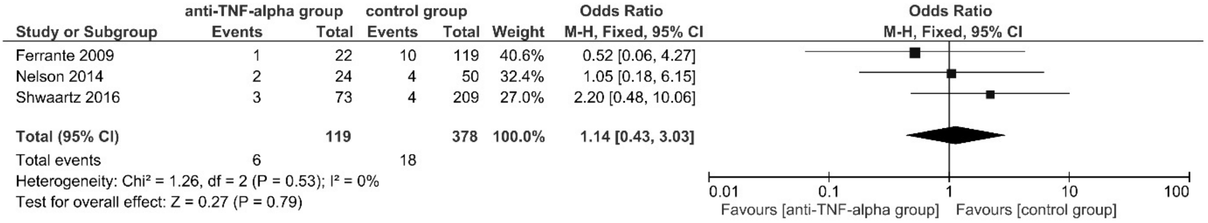

Figure S16: Fixed-effects model meta-analysis for 30-day overall infectious non-surgical-site postoperative complications in the anti-TNF-α (intervention) and control group. Forest plot of all studies included.

Study or Subgroup	anti-TNF-alpha group Events	Total	control group Events	Total	Weight	Odds Ratio M-H, Fixed, 95% CI
Bregnbak 2012	0	20	1	51	3.9%	0.82 [0.03, 21.00]
Krane 2013	5	142	21	376	50.9%	0.62 [0.23, 1.67]
Kunitake 2008	3	101	2	312	4.4%	4.74 [0.78, 28.81]
Nelson 2014	2	24	5	50	13.6%	0.82 [0.15, 4.56]
Rizzo 2011	1	54	0	60	2.1%	3.39 [0.14, 85.05]
Syed 2013	10	150	4	175	15.8%	3.05 [0.94, 9.95]
Zittan 2016	1	27	23	562	9.3%	0.90 [0.12, 6.93]
**Total (95% CI)**		**518**		**1586**	**100.0%**	**1.30 [0.75, 2.25]**
Total events	22		56			

Heterogeneity: Chi² = 6.96, df = 6 (P = 0.32); I² = 14%
Test for overall effect: Z = 0.95 (P = 0.34)

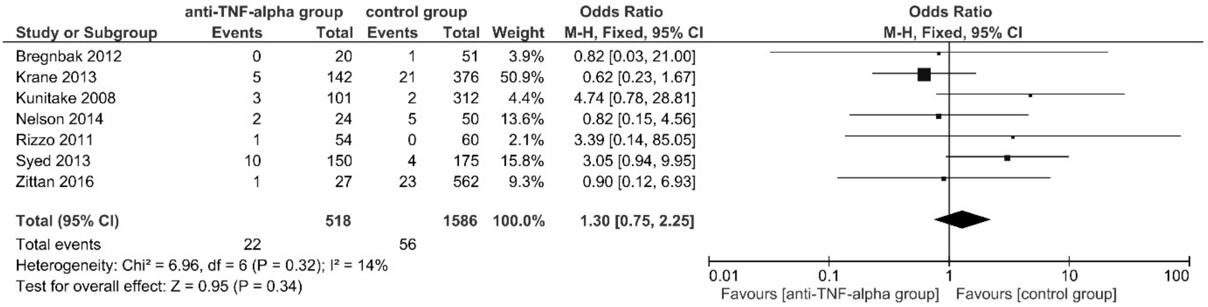

Figure S17: Fixed-effects model meta-analysis for 30-day postoperative thrombosis in the anti-TNF-α (intervention) and control group. Forest plot of all studies included.

Study or Subgroup	Experimental Events	Total	Control Events	Total	Weight	Odds Ratio M-H, Fixed, 95% CI
Krane 2013	0	142	2	376	13.2%	0.53 [0.03, 11.02]
Kunitake 2008	0	101	2	312	11.8%	0.61 [0.03, 12.85]
Nasir 2010	3	119	8	251	48.4%	0.79 [0.20, 3.02]
Syed 2013	1	150	3	175	26.6%	0.38 [0.04, 3.74]
**Total (95% CI)**		**512**		**1114**	**100.0%**	**0.62 [0.23, 1.73]**
Total events	4		15			

Heterogeneity: Chi² = 0.30, df = 3 (P = 0.96); I² = 0%
Test for overall effect: Z = 0.91 (P = 0.36)

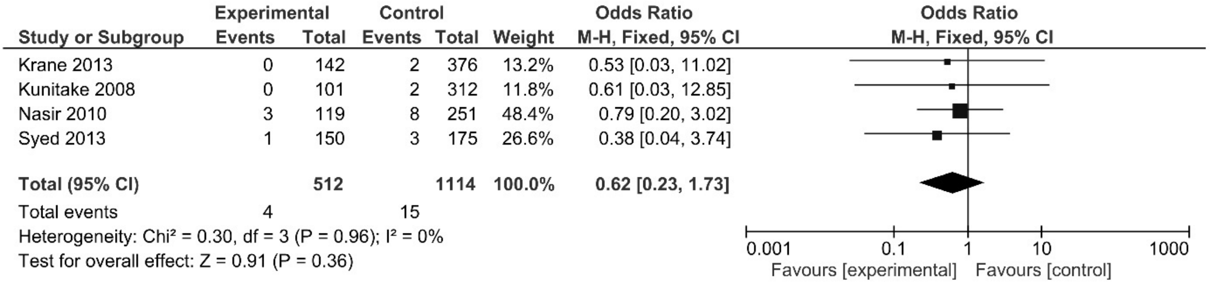

Figure S18: Fixed-effects model meta-analysis for 30-day postoperative cardiovascular complications in the anti-TNF-α (intervention) and control group. Forest plot of all studies included.

Study or Subgroup	anti-TNF-alpha group Events	Total	control group Events	Total	Weight	Odds Ratio M-H, Fixed, 95% CI
Bregnbak 2012	1	20	1	51	2.0%	2.63 [0.16, 44.22]
Canedo 2010	1	65	3	160	6.2%	0.82 [0.08, 8.01]
Gu 2013 (2)	2	142	2	265	5.0%	1.88 [0.26, 13.48]
Kim 2020	0	74	1	54	6.3%	0.24 [0.01, 5.99]
Kotze 2017	7	71	3	52	11.4%	1.79 [0.44, 7.26]
Kotze 2018	0	25	2	25	9.0%	0.18 [0.01, 4.04]
Krane 2013	0	142	1	376	3.0%	0.88 [0.04, 21.69]
Syed 2013	4	150	8	175	26.3%	0.57 [0.17, 1.94]
Waterman 2012	3	104	9	278	17.4%	0.89 [0.24, 3.35]
Yamada 2017 (1)	1	96	3	121	9.6%	0.41 [0.04, 4.04]
Yamada 2017 (2)	0	33	2	129	3.7%	0.76 [0.04, 16.24]
**Total (95% CI)**		**922**		**1686**	**100.0%**	**0.83 [0.48, 1.46]**
Total events	19		35			

Heterogeneity: Chi² = 4.66, df = 10 (P = 0.91); I² = 0%
Test for overall effect: Z = 0.64 (P = 0.52)

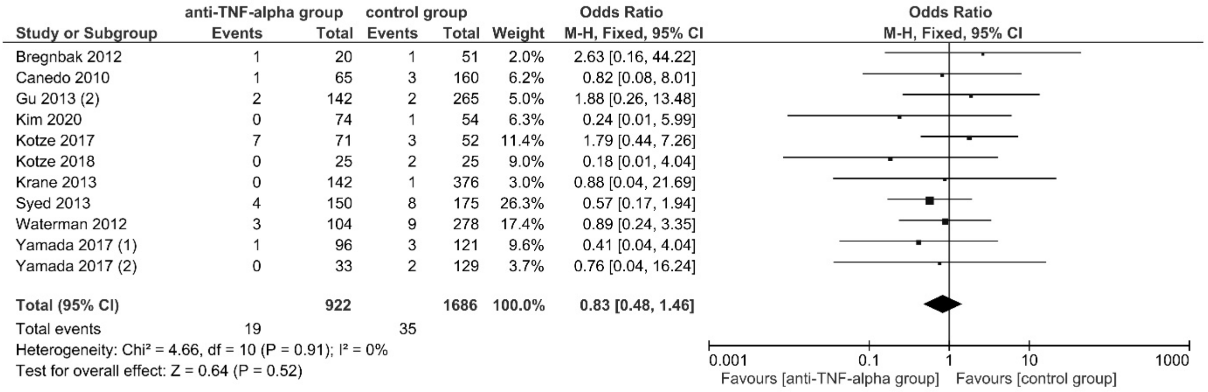

Figure S19: Fixed-effects model meta-analysis for 30-day postoperative pneumonia in the anti-TNF-α (intervention) and control group. Forest plot of all studies included.

Study or Subgroup	anti-TNF-alpha group Events	Total	control group Events	Total	Weight	Odds Ratio M-H, Fixed, 95% CI
Appau 2008	1	60	0	329	0.8%	16.61 [0.67, 412.69]
Bregnbak 2012	0	20	1	51	4.4%	0.82 [0.03, 21.00]
Ferrante 2009	1	22	5	119	7.8%	1.09 [0.12, 9.77]
Gu 2013 (2)	2	142	8	265	29.0%	0.46 [0.10, 2.19]
Kotze 2017	1	71	2	52	12.0%	0.36 [0.03, 4.05]
Kotze 2018	0	25	1	25	7.7%	0.32 [0.01, 8.25]
Krane 2013	0	142	1	376	4.3%	0.88 [0.04, 21.69]
Waterman 2012	6	104	11	278	29.7%	1.49 [0.54, 4.13]
Yamada 2017 (2)	1	33	2	129	4.2%	1.98 [0.17, 22.58]
**Total (95% CI)**		**619**		**1624**	**100.0%**	**1.02 [0.54, 1.91]**
Total events	12		31			

Heterogeneity: Chi² = 5.95, df = 8 (P = 0.65); I² = 0%
Test for overall effect: Z = 0.05 (P = 0.96)

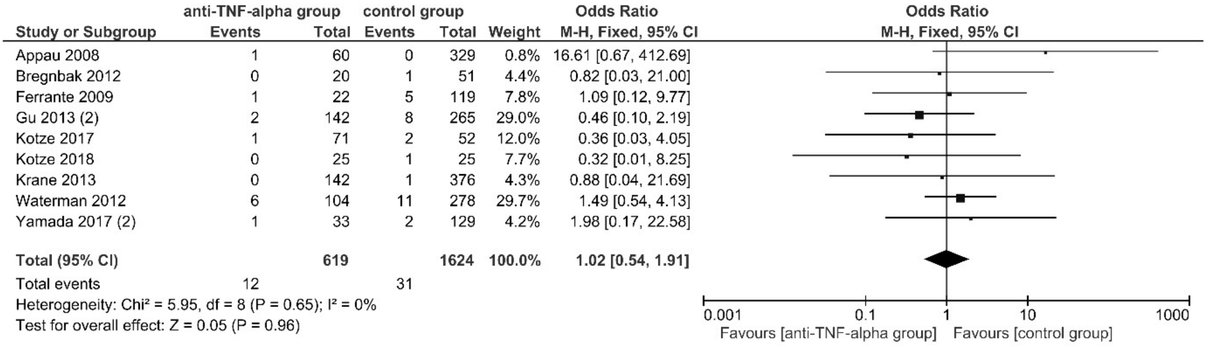

Figure S20: Fixed-effects model meta-analysis for 30-day postoperative urinary tract infections in the anti-TNF-α (intervention) and control group. Forest plot of all studies included.

Study or Subgroup	anti-TNF-alpha group Events	Total	control group Events	Total	Weight	Odds Ratio M-H, Fixed, 95% CI
Appau 2008	12	60	32	329	29.6%	2.32 [1.12, 4.82]
Bregnbak 2012	0	20	1	51	3.2%	0.82 [0.03, 21.00]
Ferrante 2009	0	22	3	119	4.1%	0.74 [0.04, 14.82]
Regadas 2011	0	28	2	221	2.1%	1.54 [0.07, 32.90]
Syed 2013	11	150	17	175	54.5%	0.74 [0.33, 1.62]
Tang 2020	1	85	4	305	6.5%	0.90 [0.10, 8.12]
**Total (95% CI)**		**365**		**1200**	**100.0%**	**1.24 [0.75, 2.04]**
Total events	24		59			

Heterogeneity: Chi² = 4.79, df = 5 (P = 0.44); I² = 0%
Test for overall effect: Z = 0.83 (P = 0.41)

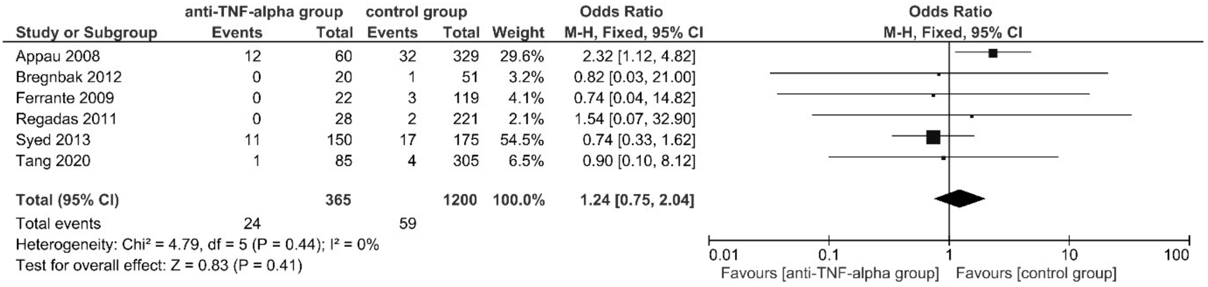

Figure S21: Fixed-effects model meta-analysis for 30-day postoperative sepsis in the anti-TNF-α (intervention) and control group. Forest plot of all studies included.

Table S1.1.-1.7.: Search strategy

**Supplementary Table S1.1.** Search strategy. Electronic medical database PubMed.				
**Search concept**		**Number of search results (n) (Name abbreviation of author; date (DD.MM.JJJJ))**		
**Concept 1**	**Inflammatory bowel diseases**	K.C.; 17.10.2021	K.C.; 04.11.2021	S.R.; 17.02.2022
Keywords:	“inflammatory bowel disease*”[tw] OR “crohns disease”[tw] OR “ulcerative colitis”[tw] OR “indeterminate colitis”[tw] OR “inflammatory bowel disease unclassified”[tw]			
Mesh:	"Inflammatory Bowel Diseases"[Mesh]			
Search:	"Inflammatory Bowel Diseases"[Mesh] OR “inflammatory bowel disease*”[tw] OR “crohns disease”[tw] OR “ulcerative colitis”[tw] OR “indeterminate colitis”[tw] OR “inflammatory bowel disease unclassified” [tw]	114906	115300	117455
**Concept 2**	**Abdominal surgery**	K.C.; 17.10.2021	K.C.; 04.11.2021	S.R.; 17.02.2022
Keywords:	“colectom*”[tw] OR “IPAA”[tw] OR “bowel resection”[tw] OR “anastomo*”[tw] OR “abdominal surgery”[tw]			
Mesh:	"Digestive System Surgical Procedures"[Mesh]			
Search:	"Digestive System Surgical Procedures"[Mesh] OR: “colectomy*”[tw] OR “IPAA”[tw] OR “bowel resection”[tw] OR “anastomo*”[tw] OR “abdominal surgery”[tw]	506973	508724	515352
**Concept 3**	**Postoperative complications**	K.C.; 17.10.2021	K.C.; 04.11.2021	S.R.; 17.02.2022
Keywords:	“postoperative complication*”[tw] OR “postoperative outcome*”[tw]			
Mesh:	"Postoperative Complications"[Mesh]			
Search:	"Postoperative Complications"[Mesh] OR “postoperative complication*”[tw] OR “postoperative outcome*”[tw]	626362	628255	636911
**Concept 4**	**Tumor necrosis factor-alpha**	K.C.; 17.10.2021	K.C.; 04.11.2021	S.R.; 17.02.2022
Keywords:	“tumor necrosis factor-alpha”[tw] OR “TNF”[tw] OR “infliximab”[tw] OR “Humira”[tw] OR“Remicade”[tw] OR “Adalimumab”[tw] OR “Golimumab”[tw] OR “Certolizumab Pegol”[tw]			

Mesh:	"Antibodies, Monoclonal"[Mesh]			
Search:	"Antibodies, Monoclonal"[Mesh] OR “tumor necrosis factor-alpha”[tw] OR “TNF”[tw] OR “infliximab”[tw] OR “Humira”[tw] OR “Remicade”[tw] OR “Adalimumab”[tw] OR “Golimumab”[tw] OR “Certolizumab Pegol”[tw]	498473	500291	509913
**Final search**		K.C.; 17.10.2021	K.C.; 04.11.2021	S.R.; 17.02.2022
Search:	((("Inflammatory Bowel Diseases"[Mesh] OR "inflammatory bowel disease*"[tw] OR "crohns disease"[tw] OR "ulcerative colitis"[tw] OR "indeterminate colitis"[tw] OR "inflammatory bowel disease unclassified"[tw]) AND ("Digestive System Surgical Procedures"[Mesh] OR "colectom*"[tw] OR "IPAA"[tw] OR "bowel resection"[tw] OR "anastomo*"[tw] OR "abdominal surgery"[tw])) AND ("Postoperative Complications"[Mesh] OR "postoperative complication*"[tw] OR "postoperative outcome*"[tw])) AND ("Antibodies, Monoclonal"[Mesh] OR "tumor necrosis factor-alpha"[tw] OR "TNF"[tw] OR "infliximab"[tw] OR "Humira"[tw] OR "Remicade"[tw] OR "Adalimumab"[tw] OR "Golimumab"[tw] OR "Certolizumab Pegol"[tw])	213	214	219

Supplementary Table S1.2. Search strategy. Electronic medical database Web of Science.

Search concept		Number of search results (n) (Name abbreviation of author; Date (DD.MM.JJJJ))		
**Concept 1**	**Inflammatory bowel diseases**	K.C.; 17.10.2021	K.C.; 04.11.2021	S.R.; 17.02.2022
Search:	((((((TS=(inflammatory bowel disease)) OR TS=(crohns disease)) OR TS=(ulcerative colitis)) OR TS=(indeterminate colitis)) OR TS=(inflammatory bowel disease unclassified)) OR TS=(crohn*)) OR TS=(ulcera* colit*)	157411	157872	161407
**Concept 2**	**Abdominal surgery**	K.C.; 17.10.2021	K.C.; 04.11.2021	S.R.; 17.02.2022
Search:	((((TS=(colectom*)) OR TS=(bowel resection)) OR TS=(anastomo*)) OR TS=(abdominal surgery)) OR TS=(IPAA)	161137	161552	164184

**Concept 3**	**Postoperative complications**	K.C.; 17.10.2021	K.C.; 04.11.2021	S.R.; 17.02.2022
Search:	(TS=(postoperative complication*)) OR TS=(postoperative outcome)	231889	233021	239415
**Concept 4**	**Tumor necrosis factor-alpha**	K.C.; 17.10.2021	K.C.; 04.11.2021	S.R.; 17.02.2022
Search:	(((((((TS=(tumor necrosis factor alpha)) OR TS=(TNF)) OR TS=(infliximab)) OR TS=(humira)) OR TS=(remicade)) OR TS=(adalimumab)) OR TS=(golimumab)) OR TS=(certolizumab pegol)	309243	310398	315830
**Final search**		K.C.; 17.10.2021	K.C.; 04.11.2021	S.R.; 17.02.2022
Search:	concept 1 AND concept 2 AND concept 3 AND concept 4	342	342	349

Supplementary Table S1.3. Search strategy. Electronic medical database Cochrane Library.

Search concept		Number of search results (n) (Name abbreviation of author; Date (DD.MM.JJJJ))		
**Concept 1**	**Inflammatory bowel diseases**	K.C.; 17.10.2021	K.C.; 04.11.2021	S.R.; 17.02.2022
Keywords:	“inflammatory bowel disease” OR “bowel disease” OR “inflammatory”<br>• “inflammatory bowel disease 1” OR “crohns disease” OR “crohn’s enteritis” OR “regional enteritis” OR “crohn’s disease” OR “granulomatous colitis” OR “granulomatous” OR “colitis” OR “granulomatous enteritis” OR “enteritis” OR “ileitis” OR “terminal ileitis” OR “regional ileitis” OR “regional ileitides” OR “ileocolitis”<br>• “ulcerative colitis” OR “idiopathic proctocolitis” OR “inflammatory Bowel Disease” OR “ulcerative colitis type” OR “colitis gravis”			
Mesh:	"Inflammatory Bowel Diseases"[Mesh] OR "Crohns Disease"[Mesh] OR "Colitis, Ulcerative"[Mesh]			
Search:	(Inflammatory Bowel Diseases):ti,ab,kw OR (Crohns Disease):ti,ab,kw OR (Colitis, Ulcerative):ti,ab,kw	6487	6520	6695
**Concept 2**	**Abdominal surgery**	K.C.; 17.10.2021	K.C.; 04.11.2021	S.R.; 17.02.2022

Keywords:	"procedure*"OR "digestive system surg*" OR "surg* procedure*"OR "gastrointestinal surg* procedure*" OR "gastrointestinal surg*"			
Mesh:	"Digestive System Surgical Procedures"[Mesh]			
Search:	(surg*):ti,ab,kw OR (anastomo*):ti,ab,kw OR (resection*):ti,ab,kw OR (digestive system surgical procedure*):ti,ab,kw	271460	273812	278397
**Concept 3**	**Postoperative complications**	K.C.; 17.10.2021	K.C.; 04.11.2021	S.R.; 17.02.2022
Keywords:	"postoperative complication*" OR "complication*"OR "postoperative"			
Mesh:	"Postoperative Complications"[Mesh]			
Search:	(postoperative complications):ti,ab,kw	44531	44808	45572
**Concept 4**	**Tumor necrosis factor-alpha**	K.C.; 17.10.2021	K.C.; 04.11.2021	S.R.; 17.02.2022
Keywords:	"infliximab" OR "renflexis" OR "infliximab dyyb" OR "monoclonal antibod* cA2" OR "cA2" OR "inflectra" OR "remicade" OR "infliximab-abda" OR "adalimumab" OR "amjevita" OR "adalimumab-adbm" OR "cyltezo" OR "adalimumab-atto" OR "humira" OR "D2E7" OR "certolizumab pegol" OR "CDP870" OR "Cimzia" OR "golimumab"			
Mesh:	"Antibodies, Monoclonal"[Mesh]			
Search:	(antibodies, monoclonal):ti,ab,kw OR (infliximab):ti,ab,kw OR (adalimumab):ti,ab,kw OR (certolizumab pegol):ti,ab,kw OR (golimumab):ti,ab,kw OR (TNF):ti,ab,kw OR (tumor necrosis factor-alpha):ti,ab,kw OR (vedolizumab):ti,ab,kw OR (ustekinumab):ti,ab,kw	30633	30901	31463
**Final search**		K.C.; 17.10.2021	K.C.; 04.11.2021	S.R.; 17.02.2022
Search:	concept 1 AND concept 2 AND concept 3 AND concept 4	13	13	13

**Supplementary Table S1.4.** Search strategy. Electronic medical database Scopus.				
	**Search concept**	Number of search results (n) (Name abbreviation of author; Date (DD.MM.JJJJ))		
**Concept 1**	**Inflammatory bowel diseases**	K.C.; 17.10.2021	K.C.; 04.11.2021	S.R.; 17.02.2022
Search:	TITLE ((inflammatory AND bowel AND disease) OR (ulcerative OR colitis) OR (crohns))	76147	76393	77884
**Concept 2**	**Tumor necrosis factor-alpha**	K.C.; 17.10.2021	K.C.; 04.11.2021	S.R.; 17.02.2022
Search:	TITLE (infliximab OR (anti AND tnf) OR tnf OR biologic OR adalimumab OR humira OR remicade OR golimumab OR certolizumab OR vedolizumab OR ustekinumab)	56495	56640	57550
**Concept 3**	**Abdominal surgery**	K.C.; 17.10.2021	K.C.; 04.11.2021	S.R.; 17.02.2022
Search:	TITLE (resection OR operation OR surgery OR colectomy)	816949	819022	831473
**Concept 4**	**Postoperative complications**	K.C.; 17.10.2021	K.C.; 04.11.2021	S.R.; 17.02.2022
Search:	TITLE (postoperative OR (postoperative AND complication) OR complication* OR outcome)	697020	699923	716766
**Final search**		K.C.; 17.10.2021	K.C.; 04.11.2021	S.R.; 17.02.2022
Search 1:	concept 1 AND concept 2 AND concept 3:<br><br>*(TITLE ((inflammatory AND bowel AND disease) OR (ulcerative OR colitis) OR (crohns))) AND (TITLE (infliximab OR (anti AND tnf) OR tnf OR biologic OR adalimumab OR humira OR remicade OR golimumab OR certolizumab OR vedolizumab OR ustekinumab)) AND (TITLE (resection OR operation OR surgery OR colectomy))*	106	106	108
Search 2:	concept 1 AND concept 2 AND concept 4:<br><br>*(TITLE ((inflammatory AND bowel AND disease) OR (ulcerative OR colitis) OR (crohns))) AND (TITLE (infliximab OR (anti AND tnf) OR tnf OR biologic OR adalimumab OR humira OR remicade OR golimumab OR certolizumab OR vedolizumab OR ustekinumab)) AND (TITLE (postoperative OR (postoperative AND complication) OR complication* OR outcome))*	231	231	246

Final search:	(concept 1 AND concept 2 AND concept 3) OR (concept 1 AND concept 2 AND concept 4):<br><br>*((TITLE ((inflammatory AND bowel AND disease) OR (ulcerative OR colitis) OR (crohns))) AND (TITLE (infliximab OR (anti AND tnf) OR tnf OR biologic OR adalimumab OR humira OR remicade OR golimumab OR certolizumab OR vedolizumab OR ustekinumab)) AND (TITLE (resection OR operation OR surgery OR colectomy))) OR ((TITLE ((inflammatory AND bowel AND disease) OR (ulcerative OR colitis) OR (crohns))) AND (TITLE (infliximab OR (anti AND tnf) OR tnf OR biologic OR adalimumab OR humira OR remicade OR golimumab OR certolizumab OR vedolizumab OR ustekinumab)) AND (TITLE (postoperative OR (postoperative AND complication) OR complication* OR outcome)))*	315	315	332

Supplementary Table S1.5. Search strategy. Electronic medical database ClinicalTrials.gov.

**Search concept**	
**Condition or disease**	inflammatory bowel disease
**Intervention/treatment**	anti-tumor necrosis factor-alpha
**Outcome measures**	postoperative complications
**Name abbreviation of author; Date (DD.MM.JJJJ)**	**Number of search results (n)**
K.C.; 18.10.2021	1
K.C.; 04.11.2021	1
S.R.; 17.02.2022	1

Supplementary Table S1.6. Search strategy. Electronic medical database World Health Organization Trials Registry.

Search concept	Number of search results (n) (Name abbreviation of author; Date (DD.MM.JJJJ))		
	K.C.; 18.10.2021	K.C.; 04.11.2021	S.R.; 17.02.2022
(inflammatory bowel disease OR crohns disease OR ulcerative colitis) AND (anti tumor necrosis factor OR TNF OR infliximab OR adalimumab OR certolizumab pegol OR golimumab OR humira OR remicade) AND (surgery OR resection) AND (postoperative complication OR complication OR postoperative outcome)	0	0	0

Supplementary Table S1.7. Search strategy. Additional data sets identified.

Method	Study (author and year)	Name abbreviation of author; Date (DD.MM.JJJJ)		
**Citation searching**	Ayoub et al., 2018 [76]	K.C., 04.11.2021	K.C.; 04.11.2021	S.R.; 17.02.2022
**Citation searching**	de Buck van Overstraeten et al., 2017 [77]	K.C., 04.11.2021	K.C.; 04.11.2021	S.R.; 17.02.2022
**Citation searching**	Guasch et al., 2016 [78]	K.C., 04.11.2021	K.C.; 04.11.2021	S.R.; 17.02.2022
**Citation searching**	Gudsoorkar et al., 2018 [79]	K.C., 04.11.2021	K.C.; 04.11.2021	S.R.; 17.02.2022
**Citation searching**	Guo et al., 2017 [80]	K.C., 04.11.2021	K.C.; 04.11.2021	S.R.; 17.02.2022
**Citation searching**	Kim et al., 2018 [81]	K.C., 04.11.2021	K.C.; 04.11.2021	S.R.; 17.02.2022
**Citation searching**	Oh et al., 2014 [82]	K.C., 04.11.2021	K.C.; 04.11.2021	S.R.; 17.02.2022
**Citation searching**	Rizvi et al., 2019 [83]	K.C., 04.11.2021	K.C.; 04.11.2021	S.R.; 17.02.2022
**Citation searching**	Schils et al., 2019 [84]	K.C., 04.11.2021	K.C.; 04.11.2021	S.R.; 17.02.2022
**Website search**	Brzezinski et al., 2002 [85]	K.C., 04.11.2021	K.C.; 04.11.2021	S.R.; 17.02.2022
**Website search**	Lau et al., 2013 [86]	K.C., 04.11.2021	K.C.; 04.11.2021	S.R.; 17.02.2022
**Website search**	Lau et al., 2013 [87]	K.C., 04.11.2021	K.C.; 04.11.2021	S.R.; 17.02.2022
**Website search**	de Silva et al., 2011 [88]	K.C., 04.11.2021	K.C.; 04.11.2021	S.R.; 17.02.2022
**Website search**	Desai et al., 2012 [89]	K.C., 04.11.2021	K.C.; 04.11.2021	S.R.; 17.02.2022
**Website search**	García et al., 2021 [90]	K.C., 04.11.2021	K.C.; 04.11.2021	S.R.; 17.02.2022

**Website search**	Karjalainen et al., 2021[91]	K.C., 04.11.2021	K.C.; 04.11.2021	S.R.; 17.02.2022
**Website search**	Marchal et al., 2004 [92]	K.C., 04.11.2021	K.C.; 04.11.2021	S.R.; 17.02.2022
**Website search**	Melo-Pinto et al., 2018 [30]	K.C., 04.11.2021	K.C.; 04.11.2021	S.R.; 17.02.2022
**Website search**	Tang et al., 2020 [60]	K.C., 04.11.2021	K.C.; 04.11.2021	S.R.; 17.02.2022
**Website search**	Uchino et al., 2010 [93]	K.C., 04.11.2021	K.C.; 04.11.2021	S.R.; 17.02.2022
**Website search**	Weber et al., 2017 [94]	K.C., 04.11.2021	K.C.; 04.11.2021	S.R.; 17.02.2022

Table S2.1.- 2.51.: Newcastle Ottawa Quality (NOS) [22] Assessment to assess the quality of included cohort studies

Table S2.1. NOS assessment [22] – Study ID: Appau et al., 2008 [25]

Criteria for cohort studies	Authors judgement	Support for judgement
Selection		
Representativeness of the exposed cohort	⋆	*"All patients undergoing surgery in the department of colorectal surgery at the Cleveland Clinic are currently accrued into an institution review board approved Crohn's disease database."* [25]; *"From this database, data of all contemporary and historical cohort patients undergoing ileocolonic resection before and after 1998 were identified […]"* [25]
Selection of the non-exposed cohort	⋆	The exposed and non-exposed cohort were obtained from the same database, hospital and time period (1998 – 2007): *"The 60 IFX patients were compared with 329 contemporary cohort patients undergoing ileocolonic resection who had never received IFX (non-IFX group)."* [25]
Ascertainment of exposure	⋆	*"Medication use was verified with the pharmacy department at the Cleveland Clinic Foundation."* [25]
Demonstration that outcome of interest was not presented at start of study	-	In total, 167 patients presented with an abscess before surgery (23 in the exposed and 144 in the non-exposed cohort). [25]
Comparability		
Comparability of cohorts on the basis of the design and analysis: controlled for a critical factor and or other medication	⋆	Adjusted for medication use (IFX; 6-MP; AZA and corticosteroids): *"Using Cox multivariate analysis to adjust for differences in medication use, age, gender, comorbidity, disease phenotypes […]"* [25]
Comparability of cohorts on the basis of the design and analysis: controlled for additional factor	⋆	Adjusted for comorbidity, penetrating abscess, diverting stoma: *"Using Cox multivariate analysis to adjust for differences in medication use, age, gender, comorbidity, disease phenotypes […]"* [25]
Outcome		
Assessment of outcome	⋆	Medical charts were reviewed: *"[…] Charts were reviewed, and all patients who took IFX contacted over the telephone to confirm the last dose of IFX infusion before ileocolonic resection."* [25]
Was the follow-up long enough for outcomes to occur	⋆	*"Outcomes evaluated included 30-day mortality, wound infection, wound complications, anastomotic leak, sepsis, intraabdominal abscess, and readmissions rate."* [25]

Adequacy of follow up of cohorts	-	No statement.
**Total score**	⋆ ⋆ ⋆ ⋆ ⋆ ⋆ ⋆ (7)	

**Table S2.2.** NOS assessment [22] – Study ID: Bafford et al., 2013 [26]		
**Criteria for cohort studies**	**Authors judgement**	**Support for judgement**
Selection		
Representativeness of the exposed cohort	⋆	*"Study subjects were identified through a prospectively maintained relational database of patients with Crohn's disease undergoing intestinal surgery by colon and rectal surgeons at the Mount Sinai Medical Center between June 1999 and May 2010. After institutional review board approval was granted, patient information was gathered from the database and supplemented with chart review. Patients who received thiopurines and anti-TNF agents within 3 months of surgery or corticosteroids for at least 7 days within 6 weeks of surgery were compared with those who did not. Medications were analyzed both individually and in combination."*
Selection of the non-exposed cohort	⋆	The exposed and non-exposed cohort were obtained from the same database, hospital and time period: *"Patients who received thiopurines and anti-TNF agents within 3 months of surgery or corticosteroids for at least 7 days within 6 weeks of surgery were compared with those who did not. Medications were analyzed both individually and in combination […]"* [26]
Ascertainment of exposure	⋆	Data obtained from a prospectively maintained database and medical charts were reviewed: *"Study subjects were identified through a prospectively maintained relational database of patients with Crohn's disease undergoing intestinal surgery by colon and rectal surgeons at the Mount Sinai Medical Center between June 1999 and May 2010. After institutional review board approval was granted, patient information was gathered from the database and supplemented with chart review […]"* [26]
Demonstration that outcome of interest was not presented at start of study	-	No statement.
Comparability		

Comparability of cohorts on the basis of the design and analysis: controlled for a critical factor and or other medication	-	Not adjusted for critical factor and or other medication.
Comparability of cohorts on the basis of the design and analysis: controlled for additional factor	-	Not adjusted for additional factor.
Outcome		
Assessment of outcome	⋆	Data obtained from a prospectively maintained database and medical charts were reviewed. [26]
Was the follow-up long enough for outcomes to occur	⋆	*"The primary data analysis compared the 30-day complication rate, hospital length of stay and 30-day readmission rates between group […]"* [26]
Adequacy of follow up of cohorts	-	No statement.
**Total score**	⋆ ⋆ ⋆ ⋆ ⋆ (5)	

**Table S2.3.** NOS assessment [22] – Study ID: Brouquet et al., 2018 [28]		
**Criteria for cohort studies**	**Authors judgement**	**Support for judgement**
Selection		
Representativeness of the exposed cohort	⋆	*"From September 1, 2013, to September 1, 2015, all patients who underwent surgery for ileocolonic CD at 19 French academic centers of the GETAID Chirurgie group, which specializes in inflammatory bowel disease (IBD) management, were prospectively included."* [28]
Selection of the non-exposed cohort	⋆	The exposed and non-exposed cohort were obtained from the same database, hospitals and time period. [28]
Ascertainment of exposure	⋆	Data on exposure were prospectively collected on an electronic dedicated clinical research form: *"Variables including demographics, disease type and severity, previous treatment of CD, intraoperative findings, and surgical procedures were prospectively collected on an electronic dedicated clinical research form (Cleanweb)."* [28]
Demonstration that outcome of interest was not presented at start of study	-	Intraoperatively, 35% of patients (208 out of 592) presented with an internal fistula and 18% of patients (106 out of 592) with an abscess. [28]

Comparability		
Comparability of cohorts on the basis of the design and analysis: controlled for a critical factor and or other medication	⋆	Multivariate analysis for: use of systemic steroids, use of budesonide.[28]
Comparability of cohorts on the basis of the design and analysis: controlled for additional factor	⋆	Multivariate analysis for: hypertension, number of acute episodes >3, recurrent CD, multifocal CD, hemoglobin level <10 g/dL, total parenteral nutrition, laparoscopic approach and operative time >180 minutes: *"In the multivariate analysis, anti-TNF <3 months prior to surgery was identified as an independent risk factor for the overall postoperative morbidity (odds-ratio [OR]=1.99; confidence interval [CI] 95%=[1.17–3.39], P = 0.011), with preoperative hemoglobin <10 g/dL (OR = 4.77; CI 95%=[1.32–17.35], P = 0.017), operative time >180 min. (OR = 2.71; CI 95=[1.54–4.78], P <0.001) and recurrent CD (OR = 1.95; CI 95%=[1.13–3.36], P = 0.017). The risk of postoperative complications increased significantly according to the number of risk factors present in the same patient. The postoperative complication rate was 18% among the 262 patients with no risk factor for postoperative morbidity, 33% among the 233 patients with 1 risk factor, and 52% among the 97 patients with 2 risk factors or more (Fig. 1) […]"* [28]
Outcome		
Assessment of outcome	⋆	Data were prospectively collected on an electronic dedicated clinical research form. [28]
Was the follow-up long enough for outcomes to occur	⋆	*"The in-hospital or 30-day postoperative morbidity and mortality were recorded prospectively starting from the date of the surgery."* [28]
Adequacy of follow up of cohorts	-	No statement.
**Total score**	⋆ ⋆ ⋆ ⋆ ⋆ ⋆ ⋆ (7)	

Table S2.4. NOS assessment [22] – Study ID: Canedo et al., 2011 [29]

Criteria for cohort studies	Authors judgement	Support for judgement
Selection		

Representativeness of the exposed cohort	⋆	*"After Institutional Review Board (IRB) approval, a retrospective analysis of a prospectively accrued surgical database was performed. Consecutive patients from 1 January 2000 to 30 April 2008 with CD, who underwent surgical intestinal and colorectal resection, were included."* [29]
Selection of the non-exposed cohort	⋆	The exposed and non-exposed cohort were obtained from the same database, hospitals and time period: *"Two hundred and twenty-five patients with CD underwent intestinal and/or colorectal resection during the study period. Group IFX (n = 65) included patients who were actively receiving infliximab or infliximab and steroids and or immunosuppressive drugs. Group OD included 85 patients who were actively receiving steroids and/or immunosuppressive drugs but not infliximab. Group ND (n = 75) included patients not receiving any medication except 5-ASA compounds."* [29]
Ascertainment of exposure	⋆	Exposure was controlled by a retrospective analysis of a prospectively accrued surgical database. [29]
Demonstration that outcome of interest was not presented at start of study	-	Fistula/abscess were the indication for surgery in 72 patients: 36,9 % in the exposed cohort (24 out of 65 patients), 30% in the non-exposed cohort (48 out of 160 patients). *[29]*
Comparability		
Comparability of cohorts on the basis of the design and analysis: controlled for a critical factor and or other medication	-	Not adjusted for critical factor and or other medication.
Comparability of cohorts on the basis of the design and analysis: controlled for additional factor	-	Not adjusted for additional factor.
Outcome		
Assessment of outcome	⋆	A retrospective analysis of a prospectively accrued database was performed. [29]
Was the follow-up long enough for outcomes to occur	⋆	A 30-day postoperative follow-up period was reported. [29]
Adequacy of follow up of cohorts	⋆	*"Due to the small number of complications, incomplete data were excluded."* [29]

**Total score**	⋆ ⋆ ⋆ ⋆ ⋆ ⋆ (6)	

**Table S2.5.** NOS assessment [22] – Study ID: El-Hussuna et al., 2012 [31]		
**Criteria for cohort studies**	**Authors judgement**	**Support for judgement**
Selection		
Representativeness of the exposed cohort	⋆	*"The study comprises operations for Crohn's disease at four different Danish hospitals in the period 2000–2007. All operations with resection and anastomosis or with stricturoplasty were included in the study."* [31]
Selection of the non-exposed cohort	⋆	The exposed and non-exposed cohort were obtained from the same hospitals and time period: *"In total, 417 consecutive operations for Crohn's disease with anastomosis and/or stricturoplasty from four hospitals were investigated for postoperative intraabdominal septic complications (IASC)"; "Thirty-two patients were on biologic treatment thirty-one with IFX and one with certolizumab"* [31]
Ascertainment of exposure	⋆	Medical records were examined: *"Age, gender, duration of disease, number of previous laparotomies, intraabdominal infection as preoperative abscess or fistula, and medication for Crohn's disease were recorded preoperatively."* [31]
Demonstration that outcome of interest was not presented at start of study	-	Overall, 20,4 % of patients (85 out of 417) presented with an intraabdominal infection preoperatively. [31]
Comparability		
Comparability of cohorts on the basis of the design and analysis: controlled for a critical factor and or other medication	⋆	Multivariate analysis was conducted for preoperative use of immunomodulators; biologics; corticosteroids and urgency of surgery. [31]

Comparability of cohorts on the basis of the design and analysis: controlled for additional factor	⋆	Multivariate analysis was conducted for demographic data (age; sex; disease duration and previous laparotomy); anastomotic technique, type and number; operation time and perioperative bleeding. [31]
Outcome		
Assessment of outcome	-	No statement.
Was the follow-up long enough for outcomes to occur	⋆	*"In the postoperative course, any adverse event or complication, readmission, or death within 30 days was registered."* [31]
Adequacy of follow up of cohorts	-	No statement.
**Total score**	⋆ ⋆ ⋆ ⋆ ⋆ ⋆ (6)	

Table S2.6. NOS assessment [22] – Study ID: Gutierrez et al., 2019 [36]

Criteria for cohort studies	Authors judgement	Support for judgement
Selection		
Representativeness of the exposed cohort	⋆	*"We retrospectively reviewed the records of patients at 26 Spanish IBD units. No data were collected prospectively. The study was approved by the corresponding Research Ethics Committees. […] We identified patients from IBD outpatient clinics aged more than 18 years who underwent ileocolonic resection with ileocolonic anastomosis for CD between January 2007 and December 2010."* [36]
Selection of the non-exposed cohort	⋆	The exposed and non-exposed cohort were obtained from the same hospitals and time period: *"Data from 364 CD patients were analyzed with a median age at time of surgery of 38 years (interquartile range [IQR] 30–48). The patients' demographic and clinical characteristics are summarized in Table 1. […] 64 (17.6%) to biological treatments […]"* [36]
Ascertainment of exposure	⋆	Medical records were examined: *"Those who signed an informed consent authorizing the use of their clinical data for research were included. For this analysis, patients' data from diagnosis of CD to the index surgery and until 5 years after the index surgery were collected from medical records."* [36]

Demonstration that outcome of interest was not presented at start of study	-	No statement.
Comparability		
Comparability of cohorts on the basis of the design and analysis: controlled for a critical factor and or other medication	-	Not adjusted for critical factor and or other medication by conducting a multivariate analysis, only a univariate analysis was performed. *[36]*
Comparability of cohorts on the basis of the design and analysis: controlled for additional factor	⋆	Partially adjusted for critical factor by conducting a multivariate analysis (surgical characteristics): *"Models were compared based on baseline clinical variables and surgical characteristics using a maximum-likelihood test, with an evaluation of possible interactions. Variables with P<0.15 in the bivariate analysis were selected for the multivariate analysis."* [36]
Outcome		
Assessment of outcome	⋆	Medical records were examined: *"Data collected from the medical records included demographic data […] We evaluated the rate of ePOCs during the 30 days after surgery, including death, ileus, anastomotic leak, digestive bleeding, abscess, wound infection, catheter-related infection and other extra-abdominal infections. We performed a separate analysis of infectious complications (wound infections, intraabdominal abscesses, extra-abdominal infections and catheter-related infections)."* [36]
Was the follow-up long enough for outcomes to occur	⋆	*"We evaluated the rate of ePOCs during the 30 days after surgery, including death, ileus, anastomotic leak, digestive bleeding, […]"* [36]
Adequacy of follow up of cohorts	⋆	*"Missing values and their frequency were tabulated but included in calculating the percentages."* [36]
**Total score**	⋆ ⋆ ⋆ ⋆ ⋆ ⋆ ⋆ (7)	

**Table S2.7.** NOS assessment [22] – Study ID: Indar et al., 2009 [37]		
**Criteria for cohort studies**	**Authors judgement**	**Support for judgement**
Selection		
Representativeness of the exposed cohort	⋆	*"A Mayo Clinic Institutional Review Board approved review of medical records was performed on consecutive patients with histologically proven Crohn's disease who had undergone intestinal surgery at Mayo Clinic, Arizona, from January 1999 to May 2007. Patients undergoing local anorectal procedures were not included."* [37]
Selection of the non-exposed cohort	⋆	The exposed and non-exposed cohort were obtained from the same database, hospitals and time period: *"Data extracted from patient charts included demographics, duration of disease, surgical procedure, blood loss, and history of corticosteroids, immunomodulators (azathioprine, 6-mercaptopurine), and anti-TNFa antibodies used for CD, as well as the occurrence of 30-day postoperative complications was also recorded."*
Ascertainment of exposure	⋆	Medical records and patient charts were reviewed: *"Data extracted from patient charts included demographics, duration of disease, surgical procedure, blood loss, and history of corticosteroids, immunomodulators (azathioprine, 6-mercaptopurine), and anti-TNFa antibodies used for CD, as well as the occurrence of 30-day postoperative complications was also recorded."* [37]
Demonstration that outcome of interest was not presented at start of study	-	Overall, 16% of patient's indication for surgery was perforation (free and abscessed). [37]
Comparability		
Comparability of cohorts on the basis of the design and analysis: controlled for a critical factor and or other medication	-	Not adjusted for critical factor and or other medication.
Comparability of cohorts on the basis of the design and analysis: controlled for additional factor	-	Not adjusted for additional factor.
Outcome		
Assessment of outcome	⋆	Medical records and patient charts were reviewed: *"Data extracted from patient charts included demographics, duration of disease, surgical procedure, blood loss, and history of corticosteroids, immunomodulators (azathioprine, 6-mercaptopurine), and anti-TNFa antibodies used for CD, as well as the occurrence of 30-day postoperative complications was also recorded."* [37]

Was the follow-up long enough for outcomes to occur	⋆	*"Data extracted from patient charts included demographics, duration of disease, surgical procedure, blood loss, and history of corticosteroids, immunomodulators (azathioprine, 6-mercaptopurine), and anti-TNFa antibodies used for CD, as well as the occurrence of 30-day postoperative complications was also recorded."* [37]
Adequacy of follow up of cohorts	-	No statement.
**Total score**	⋆ ⋆ ⋆ ⋆ ⋆ (5)	

**Table S2.8.** NOS assessment [22] – Study ID: Jouvin et al., 2018 [38]		
**Criteria for cohort studies**	**Authors judgement**	**Support for judgement**
Selection		
Representativeness of the exposed cohort	⋆	*"Three hundred and seventy-nine patients underwent ICR for CD between January 2002 and December 2013. Nineteen patients were excluded due to missing data or exclusion criteria; therefore 360 patients were included. […] For retrospective studies in France, no institutional review board approval is required. In the department, all patients signed a nonopposition form for the use of their specimen/clinical data. The database was anonymized."* [38]
Selection of the non-exposed cohort	⋆	The exposed and non-exposed cohort were obtained from the same database, hospitals and time period. [38]
Ascertainment of exposure	-	No statement.
Demonstration that outcome of interest was not presented at start of study	-	No statement.
Comparability	⋆	*We decided to give one star for comparability because the study was adjusted for some important factors, but not all.*

Comparability of cohorts on the basis of the design and analysis: controlled for a critical factor and or other medication	-	Adjusted for use of AZA by conducting a multivariate analysis. [38]
Comparability of cohorts on the basis of the design and analysis: controlled for additional factor	-	Partially adjusted for active smoking; comorbidity; history of ileocolonic resection; BMI <18.5 kg/m^2; other surgical procedures; sex and type of anastomosis by conducting multivariate analysis. [38]
Outcome		
Assessment of outcome	-	No statement.
Was the follow-up long enough for outcomes to occur	⋆	*"Morbidity was defined as the occurrence of a complication within 30 days, with the most significant complication being recorded if more than 1 complication occurred in a patient."* [38]
Adequacy of follow up of cohorts	-	No statement.
**Total score**	⋆ ⋆ ⋆ ⋆ (4)	

**Table S2.9.** NOS assessment [22] – Study ID: Kotze et al., 2017 [41]		
**Criteria for cohort studies**	**Authors judgement**	**Support for judgement**
Selection		
Representativeness of the exposed cohort	⋆	*"This was a retrospective longitudinal and observational study, with CD patients submitted to intestinal resections due to complications or failure to medical therapy, from 2 different IBD referral units from Brazil, in a 7-year period (January 2007–July 2014). […] Patients with an established diagnosis of CD with clinical, imaging, endoscopic, and histological criteria, over 18 years old at the time of surgery, submitted to any elective abdominal surgical procedure with intestinal resection, were included in the study."* [41]
Selection of the non-exposed cohort	⋆	The exposed and non-exposed cohort were obtained from the same database, hospitals and time period: *"Therefore, 123 patients composed the final population analyzed (Fig. 1). From this sample, 52 had no previous exposure to anti-TNF agents, while 71 used these agents preoperatively (39 on IFX and 32 on ADA)."* [41]
Ascertainment of exposure	⋆	Medical records were assessed: *"Initially 144 patients were identified from the surgical databases of both units, and had their records accessed."* [41]
Demonstration that outcome of interest was not presented at start of study	-	No statement.
Comparability		
Comparability of cohorts on the basis of the design and analysis: controlled for a critical factor and or other medication	⋆	Adjusted by conducting multivariate analysis for overall surgical complications (for use of previous anti-TNF agents and previous steroids): *"In multivariate analysis (logistic regression model and Wald test), it was confirmed that preoperative anti-TNF agents were not associated with an increase in surgical complications (OR 0.90; 95% CI 0.37–2.18; p = 0.815). However, previous steroids were confirmed as a risk factor associated with these types of complications (Table 3)."* [41] and for overall medical complications (use of previous anti-TNF agents and previous steroids): *"In multivariate analysis, preoperative anti-TNF agents were also not confirmed as a risk factor for medical complications (OR 0.98; IC 0.32–3.06; p = 0.976). Again, in accordance with the univariate analysis findings, hypoalbuminemia and previous steroids were associated with higher postoperative medical complications, as illustrated in Table 5 […]"* [41] .
Comparability of cohorts on the basis of the design and analysis: controlled for additional factor	⋆	Adjusted by conducting multivariate analysis for overall surgical complications (perianal CD, primary anastomosis and stomas: *"In multivariate analysis (logistic regression model and Wald test), it was confirmed that preoperative anti-TNF agents were not associated with an increase in surgical complications (OR 0.90; 95% CI 0.37–2.18; p = 0.815). However, previous steroids were confirmed as a risk factor associated with these types of*

		complications (Table 3)." [10] and for overall medical complications (hypoalbuminemia and perianal CD): *"In multivariate analysis, preoperative anti-TNF agents were also not confirmed as a risk factor for medical complications (OR 0.98; IC 0.32–3.06; p = 0.976). Again, in accordance with the univariate analysis findings, hypoalbuminemia and previous steroids were associated with higher postoperative medical complications, as illustrated in Table 5 […]"* [41]
Outcome		
Assessment of outcome	⋆	Medical records were assessed: *"Initially 144 patients were identified from the surgical databases of both units, and had their records accessed."* [41]
Was the follow-up long enough for outcomes to occur	⋆	*"Early postoperative surgical and medical complications (up to 30 days after the surgical procedure) were evaluated"* [41]
Adequacy of follow up of cohorts	-	No statement.
**Total score**	⋆ ⋆ ⋆ ⋆ ⋆ ⋆ ⋆ (7)	

**Table S2.10.** NOS assessment [22] – Study ID: Kotze et al., 2018 [40]		
**Criteria for cohort studies**	**Authors judgement**	**Support for judgement**
Selection		
Representativeness of the exposed cohort	⋆	*"This was a case-matched, retrospective, longitudinal observational study, with CD patients submitted to intestinal resections due to complications or failure of medical therapy, from 2 different IBD referral units in Brazil, over a 7-year period. […] Patients with an established diagnosis of CD with clinical, imaging, endoscopic and histological criteria, over 18 years old, submitted to any elective abdominal surgical procedure with intestinal resection, on ADA or conventional therapy preoperatively, were initially included in the study."* [40]
Selection of the non-exposed cohort	⋆	The exposed and non-exposed cohort were obtained from the same database, hospitals and time period: *"Therefore, 123 patients were considered for inclusion in the study. Of these, 52 had no previous exposure to anti-TNF agents (conventional therapy), while 71 used these agents preoperatively (39 on IFX and 32 on ADA). The 39 IFX patients were excluded […]"* [40]
Ascertainment of exposure	⋆	Medical records were assessed: *"Initially, 144 patients were identified and had their records accessed."* [40]
Demonstration that outcome of interest was not presented at start of study	-	Overall, 17 patients presented with fistula/ abscess as indication for surgery - 40% of the exposed cohort (10 out of 25 patients) and 28% of the non-exposed cohort (7 out of 25 patients) - and one of 25 patients of the exposed cohort presented with inflammation as indication for surgery. [40]
Comparability		
Comparability of cohorts on the basis of the design and analysis: controlled for a critical factor and or other medication	-	Not adjusted for critical factor and or other medication: *"A multivariate analysis could not be performed due to the lack of significant variables found in univariate analysis (two variables in surgical complications and one variable in medical complications)."* [40]
Comparability of cohorts on the basis of the design and analysis: controlled for additional factor	⋆	Propensity score method case-matched study: *"After including the selected patients in the software, the PSM finally matched 25 ADA patients with 25 patients under conventional therapy in a 1:1 ratio, according to the main variables previously mentioned (age at surgery, CD location and phenotype)."* [40]

Outcome		
Assessment of outcome	⋆	Medical records were assessed. [40]
Was the follow-up long enough for outcomes to occur	⋆	*"Early postoperative surgical and medical complications (up to 30 days after the surgical procedure) were evaluated […]"* [40]
Adequacy of follow up of cohorts	-	No statement.
**Total score**	⋆ ⋆ ⋆ ⋆ ⋆ ⋆ (6)	

**Table S2.11.** NOS assessment [22] – Study ID: Lightner et al., 2019 [44]		
**Criteria for cohort studies**	**Authors judgement**	**Support for judgement**
Selection		
Representativeness of the exposed cohort	⋆	*"After institutional review board approval, a retrospective chart review of the Mayo Clinic Rochester electronic medical chart system between May 20, 2014, and December 31, 2017, was performed. A list of all patients with CD who underwent a major abdominal operation was obtained as identified by International Classification of Diseases, Ninth Revision (555.x), and International Classification of Diseases, Tenth Revision, codes for CD (50.x) and Current Procedural Terminology codes for major abdominal surgery […] 45119, 45120, 45136, 45395, 45397, 45800, 45805, 45820, 45825, 49000)."* [44]
Selection of the non-exposed cohort	⋆	The exposed and non-exposed cohort were obtained from the same database, hospitals and time period: *"Study patients included adults (aged ≥18 y) with CD who received an anti-TNF (infliximab, adalimumab, certolizumab pegol), anti-integrin (vedolizumab), or anti-interleukin (ustekinumab) within 12 weeks of a major abdominal operation. The control cohort included patients not exposed to a biologic within 12 weeks of a major abdominal operation."* [44]
Ascertainment of exposure	⋆	Electronic medical records were reviewed: *"After institutional review board approval, a retrospective chart review of the Mayo Clinic Rochester electronic medical chart system between May 20, 2014, and December 31, 2017, was performed."* [44]

Demonstration that outcome of interest was not presented at start of study	-	Exposed and non-exposed cohort presented with an increased median (interquartile range (IQR)) C-reactive protein preoperatively (median (IQR) = 6.7 (3.0-31.3) for the exposed cohort and median (IQR) = 13.1 (3.0-37.9) for the nonexposed cohort). [44]
Comparability		
Comparability of cohorts on the basis of the design and analysis: controlled for a critical factor and or other medication	-	Not adjusted for critical factor and or other medication by conducting multivariate analysis, only univariate and multivariable analysis were conducted. [44]
Comparability of cohorts on the basis of the design and analysis: controlled for additional factor	-	Not adjusted for additional factor by conducting multivariate analysis, only univariate and multivariable analysis were conducted. [44]
Outcome		
Assessment of outcome	⋆	Electronic medical records were reviewed. [44]
Was the follow-up long enough for outcomes to occur	⋆	*"The primary end points were the overall infectious complication rate (combination of nonsurgical and surgical infectious complications listed above) and the rate of intra-abdominal sepsis defined as a deep space abscess or anastomotic leak. The secondary end points were rates of 30-day postoperative nonsurgical infectious complications, 30-day surgical infectious complications, 30-day superficial SSI, 30-day readmission, 30-day return to the operating room, and 30-day mortality."* [44]
Adequacy of follow up of cohorts	⋆	*" Patients were excluded if they underwent an emergent operation, did not have ≥30 days of follow-up after their operation, or had their operation performed at an outside hospital."* [44]
**Total score**	⋆ ⋆ ⋆ ⋆ ⋆ ⋆ (6)	

Table S2.12. NOS assessment [22] – Study ID: Maeda et al., 2015 [45]

Criteria for cohort studies	Authors judgement	Support for judgement
Selection		
Representativeness of the exposed cohort	⋆	*"We retrospectively collected and reviewed the patient characteristics and perioperative data after obtaining approval from our institutional review board. A total of 177 patients with CD who underwent bowel resection at the Department of Surgical Oncology, Osaka*

		City University Hospital between January 2005 and December 2013 were included in this study." [45]
Selection of the non-exposed cohort	⋆	The exposed and non-exposed cohort were obtained from the same database, hospitals and time period: *"The clinical characteristics of the 177 patients are summarized in Table 1. […] Preoperative infliximab or adalimumab had been administered to 36.7 % (65/177) of the patients."* [45]
Ascertainment of exposure	-	No statement.
Demonstration that outcome of interest was not presented at start of study	-	Overall, 35 patients presented with an abscess at the laparotomy and another 35 patients with a perianal suppurating lesion preoperatively. [45]
Comparability	⋆	*We decided to give one star for comparability since some adjustment was taken.*
Comparability of cohorts on the basis of the design and analysis: controlled for a critical factor and or other medication		Partially adjusted for critical factor by conducting multivariate analysis (of variables with a value of $p < 0.05$ in the univariate analyses): *"In the present study, obesity (BMI ≥25) was observed in only four patients, a hand-sewn anastomosis was performed in five patients and preoperative steroids were administered to only seven patients; therefore, these factors were excluded from the statistical analysis."* [45]
Comparability of cohorts on the basis of the design and analysis: controlled for additional factor		Partially adjusted for critical factor by conducting multivariate analysis (of variables with a value of $p < 0.05$ in the univariate analyses). [45]
Outcome		
Assessment of outcome	-	No statement.
Was the follow-up long enough for outcomes to occur	⋆	*"The criteria for a diagnosis of an SSI included the occurrence of an infection within 30 days after surgery, […]"* [45]
Adequacy of follow up of cohorts	-	No statement.
**Total score**	⋆ ⋆ ⋆ ⋆ (4)	

**Table S2.13.** NOS assessment [22] – Study ID: Mascarenhas et al., 2012 [46]		
**Criteria for cohort studies**	**Authors judgement**	**Support for judgement**
Selection		
Representativeness of the exposed cohort	⋆	*"A review of patients who underwent ileocolic resections and/or right hemicolectomies from January 1, 2003, through December 31, 2010, was performed. Approval for this study was obtained from the Spectrum Health Institutional Review Board."* [46]
Selection of the non-exposed cohort	⋆	The exposed and non-exposed cohort were obtained from the same database, hospitals and time period: *"Of the 93 patients with CD, 19 patients were identified who had received biologic therapy within 3 months of Surgery […]"* [46]
Ascertainment of exposure	⋆	Medical records were reviewed: *"Data extracted from the medical record included age, sex, operating room time, major and minor complications, length of stay, medication use (eg, steroids, biologics), mortality, American Society of Anesthesiologists (ASA) score, and estimated blood loss (EBL)."* [46]
Demonstration that outcome of interest was not presented at start of study	-	No statement.
Comparability		
Comparability of cohorts on the basis of the design and analysis: controlled for a critical factor and or other medication	-	Not adjusted to critical factor and or other medication.
Comparability of cohorts on the basis of the design and analysis: controlled for additional factor	-	Not adjusted for additional factor.
Outcome		
Assessment of outcome	⋆	Medical records were reviewed: *"Only complications and mortalities that occurred within the first 30 days were included in this study."* [46]

Was the follow-up long enough for outcomes to occur	⋆	*"Only complications and mortalities that occurred within the first 30 days were included in this study."* [46]
Adequacy of follow up of cohorts	-	No Statement.
**Total score**	⋆ ⋆ ⋆ ⋆ ⋆ (5)	

**Table S2.14.** NOS assessment [22] – Study ID: Melo-Pinto et al., 2018 [30]		
**Criteria for cohort studies**	**Authors judgement**	**Support for judgement**
Selection		
Representativeness of the exposed cohort	⋆	*"In this retrospective study, all patients operated due to CD at HSJ between 1st January 2010and 31st December 2015 was included. Patients were identified by the diagnosis code for CD in the hospital registries and all the data was obtained through the electronic health registries."* [30]
Selection of the non-exposed cohort	⋆	The exposed and non-exposed cohort were obtained from the same database, hospitals and time period: *"Other therapeutic regimens included anti-TNF (62 patients; 35.8%) […]"* [30]
Ascertainment of exposure	⋆	Electronic health registries were examined: *"Patients were identified by the diagnosis code for CD in the hospital registries and all the data was obtained through the electronic health registries."* [30]
Demonstration that outcome of interest was not presented at start of study	-	Overall, 16,8% of patients (29 out of 173 patients) presented with an abscess at surgery. [30]
Comparability		
Comparability of cohorts on the basis of the design and analysis: controlled for a critical factor and or other medication	⋆	Adjusted for use of previous anti-TNF, previous ustekinumab and previous corticosteroids by conducting a multivariate analysis: *"A significant association was found between age at surgery, age at diagnosis, use of CCT, anti-TNF and ustekinumab and indication for surgery and post-operative complications in the univariate analysis. In this multivariate analysis, only the age at surgery and indication for surgery was independent risk factors for post-operative complications. Those aged 40 or more at surgery (Montreal Classification A3) had higher probability of postoperative complications comparing to those under 40 years (Montreal Classification A1 + A2) (OR = 4.12; p <*

		0.05). The composite group of other indications for surgery (intestinal perforation, mesenteric ischemia and postoperative complications) were also associated with an increase of postoperative complications comparing to the ones who underwent surgery due to occlusion or fistula/abscess (OR = 4.12; p < 0.05). Neither age at diagnosis or previous CCT/anti-TNF/ustekinumab was significantly associated with an increased risk in postoperative complications." [30]
Comparability of cohorts on the basis of the design and analysis: controlled for additional factor	⋆	Adjusted for age at diagnosis >40, age at surgery >40, surgical indication, intestinal occlusion, fistula/abscess and other: *"A significant association was found between age at surgery, age at diagnosis, use of CCT, anti-TNF and ustekinumab and indication for surgery and post-operative complications in the univariate analysis. In this multivariate analysis, only the age at surgery and indication for surgery was independent risk factors for post-operative complications. Those aged 40 or more at surgery (Montreal Classification A3) had higher probability of postoperative complications comparing to those under 40 years (Montreal Classification A1 + A2) (OR = 4.12; p < 0.05). The composite group of other indications for surgery (intestinal perforation, mesenteric ischemia and postoperative complications) were also associated with an increase of postoperative complications comparing to the ones who underwent surgery due to occlusion or fistula/abscess (OR = 4.12; p < 0.05). Neither age at diagnosis or previous CCT/anti-TNF/ustekinumab was significantly associated with an increased risk in postoperative complications."* [30]
Outcome		
Assessment of outcome	⋆	Electronic health registries were examined. [30]
Was the follow-up long enough for outcomes to occur	⋆	*"Postoperative complications data occurring within 30 days were also collected […]"* [30]
Adequacy of follow up of cohorts	-	No Statement.
**Total score**	⋆ ⋆ ⋆ ⋆ ⋆ ⋆ ⋆ (7)	

Table S2.15. NOS assessment [22] – Study ID: Morar et al., 2015 [48]

Criteria for cohort studies	Authors judgement	Support for judgement
Selection		

Representativeness of the exposed cohort	⋆	*"Institutional approval from the local board research and development committee was obtained. Data were obtained by cross-referencing three prospectively maintained databases within our institution from over a 6-year period [January 1, 2005 to December 31, 2010]. A total of 1589 ICD-10 [International Classification of Diseases] established diagnoses relating to admissions for Crohn's disease were obtained from the hospital coding department. A total of 988 positively diagnosed Crohn's disease samples from our histopathology department was also obtained. The patient identification codes for each group were cross-referenced and duplicate cases were removed. A further cross-referencing with 7298 small- and large bowel surgical procedures was performed within the same time period, obtained from OPCS [Classification of Interventions and Procedures] codes. A final figure of 236 ileocolonic Crohn's surgical procedures was obtained."* [48]
Selection of the non-exposed cohort	⋆	The exposed and non-exposed cohort were obtained from the same database, hospitals and time period. [48]
Ascertainment of exposure	⋆	Clinical case records were screened: *"The clinical case records were screened to ensure specific eligibility criteria was met as follows […]"* [48]
Demonstration that outcome of interest was not presented at start of study	-	Some patients presented with abscess or fistula intraoperatively. [48]
Comparability		
Comparability of cohorts on the basis of the design and analysis: controlled for a critical factor and or other medication	-	Adjusted for biological therapy by conducting a multivariate analysis. [48]
Comparability of cohorts on the basis of the design and analysis: controlled for additional factor	⋆	Adjusted for smoking, concomitant upper gastrointestinal crohn's disease, perioperative anemia, perioperative hypoalbuminemia and intraoperative sepsis by conducting multivariate analysis for intra-abdominal septic complication: *"Multivariate analysis adjusting for smoking status, concomitant UGI Crohn's, peri-operative biological therapy, peri-operative anaemia, peri-operative hypoalbuminaemia, intra-operative sepsis. The presence of intra-abdominal sepsis [OR 8.6, 95% CI 1.2 – 60.1; p = 0.03], and peri-operative biological therapy [OR 24.6, 95% CI: 2 – 298; p = 0.01] were demonstrated as independent risk factors for the development of post-operative intra-abdominal septic complications."* [48]
Outcome		
Assessment of outcome	⋆	Clinical case records were screened. [48]

Was the follow-up long enough for outcomes to occur	⋆	*"The primary outcome measure was a measure of the incidence of intra-abdominal septic complications within a 30-day post-operative time frame […]"*[48]
Adequacy of follow up of cohorts	-	Below 15 % of patients had missing data. [48]
**Total score**	⋆ ⋆ ⋆ ⋆ ⋆ ⋆ (6)	

Table S2.16. NOS assessment [22] – Study ID: Myrelid et al., 2014 [49]

Criteria for cohort studies	Authors judgement	Support for judgement
Selection		
Representativeness of the exposed cohort	⋆	*"All patients undergoing surgery for Crohn's disease involving one or more intestinal anastomoses and who received anti-TNF therapy either before or after surgery were identified from databases."* [49]; *"All patients receiving biologicals and undergoing abdominal surgery with anastomosis or strictureplasty were identified at six tertiary referral centres."* [49]
Selection of the non-exposed cohort	⋆	The exposed and non-exposed cohort were obtained from the same database, hospitals and time period: *"Patients were categorized into two groups: those in the treatment group received anti-TNF therapy within 2 months of surgery, whereas patients in the control group were treated with anti-TNF therapy, but had discontinued treatment more than 2 months before operation or started treatment at least 6 weeks after surgery." [17]; "All patients receiving biologicals and undergoing abdominal surgery with anastomosis or strictureplasty were identified at six tertiary referral centres."* [49]
Ascertainment of exposure	⋆	Data obtained from a database. [49]
Demonstration that outcome of interest was not presented at start of study	-	Overall, 19,8% of exposed cohort (22 out of 111) and 20,9% of non-exposed cohort (39 out of 187) presented with abscess/fistula preoperatively. [49]
Comparability		

Comparability of cohorts on the basis of the design and analysis: controlled for a critical factor and or other medication	-	Not adjusted for critical factor and or other medication by multivariate analysis, only univariate analysis and multivariable analysis were conducted. [49]
Comparability of cohorts on the basis of the design and analysis: controlled for additional factor	-	Not adjusted for additional factor by multivariate analysis, only univariate analysis and multivariable analysis were conducted. [49]
Outcome		
Assessment of outcome	⋆	Data obtained from a database. [49]
Was the follow-up long enough for outcomes to occur	⋆	*"The primary endpoint of the study was the development of anastomotic complications within 30 days or during the postoperative hospital stay […]"* [49]
Adequacy of follow up of cohorts	-	No statement.
**Total score**	⋆ ⋆ ⋆ ⋆ ⋆ (5)	

Table S2.17. NOS assessment [22] – Study ID: Nasir et al., 2010 [50]

Criteria for cohort studies	Authors judgement	Support for judgement
Selection		
Representativeness of the exposed cohort	⋆	*"A retrospective analysis was performed on all patients undergoing surgery for Crohn's disease from 2005 through 2009. Patients were identified from a prospective registry of patients undergoing surgery at Division of Colon and Rectal Surgery, Mayo Clinic, Rochester. The study was approved by the Institutional Review Board of the Mayo Foundation […]"* [50]
Selection of the non-exposed cohort	⋆	The exposed and non-exposed cohort were obtained from the same database, hospitals and time period. [50]
Ascertainment of exposure	⋆	Data obtained from a prospective registry. [50]

Demonstration that outcome of interest was not presented at start of study	-	No statement.
Comparability		
Comparability of cohorts on the basis of the design and analysis: controlled for a critical factor and or other medication	-	Not adjusted for a critical factor and or other medication.
Comparability of cohorts on the basis of the design and analysis: controlled for additional factor	-	Not adjusted for additional factor.
Outcome		
Assessment of outcome	⋆	Data obtained from a prospective registry. [50]
Was the follow-up long enough for outcomes to occur	⋆	*"Patient characteristics, disease severity, medication use, operative intervention and 30-day complication were compared between the two groups."* [50]
Adequacy of follow up of cohorts	-	No statement.
**Total score**	⋆ ⋆ ⋆ ⋆ ⋆ (5)	

Table S2.18. NOS assessment [22] – Study ID: Nørgård et al., 2013 [53]

Criteria for cohort studies	Authors judgement	Support for judgement
Selection		
Representativeness of the exposed cohort	⋆	*"In addition, the availability of nationwide Danish registries made it possible to assess data from The Danish National Patient Registry (NPR) on (i) patients with CD, (ii) treatment with anti-TNF-a agents, (iii) post-operative complications (reoperation, anastomosis leakage, percutaneous abscess drainage and bacteraemia), The Danish Medicine Agency at Statistic Denmark on, (iv) therapeutic drug use before date of surgery for CD, The Central Personal Registration system on, (v) death, and regional bacteraemia registries on, (vi) cases of bacteraemia."* [53]

Selection of the non-exposed cohort	⋆	The exposed and non-exposed cohort were obtained from the same database, hospitals and time period: *"The exposed cohort comprised 214 (9.3%) operations where patients were treated with anti- TNF-a agents within 12 weeks before surgery, and in the remaining operations, no anti-TNF-a agent was given within 12 weeks before surgery [unexposed cohort 1 = 2079 (90.7%)]."* [53]
Ascertainment of exposure	⋆	Data obtained from the National Patient Registry by codes and out-patient drug prescriptions from a nationwide prescription database maintained by Danish Medicine Agency, as well by codes. [53]
Demonstration that outcome of interest was not presented at start of study	-	No statement.
Comparability		
Comparability of cohorts on the basis of the design and analysis: controlled for a critical factor and or other medication	-	Adjusted OR for use of steroids within 4 weeks before surgery: *"We used logistic regression analyses to compute relative risk estimates for adverse post-operative outcomes (reoperations, anastomosis leakage, intra-abdominal abscess, bacteraemia, and death within 30 days and 60 days after CD operation) associated with anti-TNF-a agent therapy pre-operatively, adjusted for potential confounders. Adjustment was made for age (years: 15–25 as reference, 26–55, >55), gender (females as reference), comorbidity (Charlson Index 0 as reference, Charlson Index 1–2, Charlson Index >2), calendar period (2003–2004 as reference, 2005–2006, 2007–2008, 2009–2010), and duration of CD (<5 years as reference, ≥5 years), use of steroids within 4 weeks before surgery (no as reference, yes), number of inpatient days at hospital within 4 weeks before surgery (<2 weeks as reference, ≥2 weeks) in a logistic regression model."* [53]
Comparability of cohorts on the basis of the design and analysis: controlled for additional factor	-	Adjusted OR for age, gender, comorbidity, calendar period, duration of CD and number of inpatient days at hospital within 4 weeks before surgery: *"We used logistic regression analyses to compute relative risk estimates for adverse post-operative outcomes (reoperations, anastomosis leakage, intra-abdominal abscess, bacteraemia, and death within 30 days and 60 days after CD operation) associated with anti-TNF-a agent therapy pre-operatively, adjusted for potential confounders. Adjustment was made for age (years: 15–25 as reference, 26–55, >55), gender (females as reference), comorbidity (Charlson Index 0 as reference, Charlson Index 1–2, Charlson Index >2), calendar period (2003–2004 as reference, 2005–2006, 2007–2008, 2009–2010), and duration of CD (<5 years as reference, ≥5 years), use of steroids within 4 weeks before surgery (no as reference, yes), number of inpatient days at*

		hospital within 4 weeks before surgery (<2 weeks as reference, ≥2 weeks) in a logistic regression model." [53]
Outcome		
Assessment of outcome	⋆	Data obtained by codes from the National Patient Registry. [53]
Was the follow-up long enough for outcomes to occur	⋆	*"We evaluated all outcomes occurring within 30 and 60 days after surgery for CD."* [53]
Adequacy of follow up of cohorts	⋆	*"[…] only patients available for full follow-up were included."* [53]
**Total score**	⋆ ⋆ ⋆ ⋆ ⋆ ⋆ (6)	

Table S2.19. NOS assessment [22] – Study ID: Serradori et al., 2013 [57]

Criteria for cohort studies	Authors judgement	Support for judgement
Selection		
Representativeness of the exposed cohort	⋆	*"All adult patients (aged more than 16 years) who underwent ileocaecal or ileocolonic resection for Crohn's disease in three French referral centres (Visceral Surgery Department and Emergency Surgery Department of University Hospital of Nancy (centre 1), Visceral Surgery Department of University Hospital of Strasbourg (centre 2) and Visceral Surgery Department of University of Reims (centre 3) between January 2000 and December 2010 were included in this retrospective study. The terms 'Crohn disease', 'ileocaecal resection', 'ileo-colonic resection' and 'right colectomy' were used to identify eligible patients using the database of the Department of Medical Information. Retrospective studies do not require any ethical approval in France."* [57]
Selection of the non-exposed cohort	⋆	The exposed and non-exposed cohort were obtained from the same database, hospitals and time period: *"A total of 217 patients were included over a 10-year period, […] Anti-TNF- α (12-week washout period) 33 (17,1) No PIIC (n = 193), 9 (38%) PIIC (n =24) […]"* [57]
Ascertainment of exposure	⋆	Medical charts were reviewed: *"The charts of all included patients with an established diagnosis of Crohn's disease, according to Lennard-Jones criteria[7], were reviewed […] dose of systemic steroids, use of anti-TNF-α therapy (infliximab, adalimumab), use of immunomodulators (thiopurine, methotrexate)."* [57]

Demonstration that outcome of interest was not presented at start of study	-	Overall, 5 patients had a fistula at time of surgery (4 out of 193 patients of the no PIIC group and 1 out of 24 of the PIIC Group9, 2 had a peritonitis (1 of each group), 30 had an abscess at time of surgery (28 in the no PIIC group and 2 in the PIIC group). [57]
Comparability		
Comparability of cohorts on the basis of the design and analysis: controlled for a critical factor and or other medication	-	Not adjusted for critical factor and or other medication by conducting multivariate analysis, only multivariable analysis was conducted. [57]
Comparability of cohorts on the basis of the design and analysis: controlled for additional factor	-	Not adjusted additional factor by conducting multivariate analysis, only multivariable analysis was conducted. [57]
Outcome		
Assessment of outcome	⋆	Medical charts were reviewed. [57]
Was the follow-up long enough for outcomes to occur	⋆	*"The median time to onset of first PIIC was 10 (range 2–30) days."* [57]
Adequacy of follow up of cohorts	-	No statement. [57]
**Total score**	⋆ ⋆ ⋆ ⋆ ⋆ (5)	

Table S2.20. NOS assessment [22] – Study ID: Syed et al., 2013 [59]

Criteria for cohort studies	Authors judgement	Support for judgement
Selection		
Representativeness of the exposed cohort	⋆	*"All CD patients who underwent abdominal surgery while followed at UMB between 1 July 2004 and 1 May 2011 were identified. Patients were selected through a search of the UMB Clinical Database, a prospectively maintained Institutional Review Board approved instrument containing clinical and demographic information on all patients with inflammatory bowel disease seen at UMB since 1 July 2004. All CD patients have a verified diagnosis based on accepted criteria (22)."* [59]
Selection of the non-exposed cohort	⋆	The exposed and non-exposed cohort were obtained from the same database, hospitals and time period: *"Between July 2004 and May 2011, 325 intra-abdominal*

		surgeries were performed in 211 CD patients. […] One hundred and fifty surgeries (46.1 %) were associated with a dose of anti-TNF […]" [59]
Ascertainment of exposure	⋆	Electronic medical records and clinical charts were reviewed: *"A chart review of identified patients yielded 211 CD patients who underwent abdominal surgery during the study period. Isolated perianal surgeries were excluded. […] Review of the UMB electronic medical record (Cerner Powerchart, Kansas City, MO) and clinic charts provided operative details as well as […]"* [59]
Demonstration that outcome of interest was not presented at start of study	-	No statement.
Comparability		
Comparability of cohorts on the basis of the design and analysis: controlled for a critical factor and or other medication	-	Not adjusted for critical factor and or other medication.
Comparability of cohorts on the basis of the design and analysis: controlled for additional factor	-	Not adjusted for additional factor by conducting multivariate analysis, only multivariable analysis was performed. [59]
Outcome		
Assessment of outcome	⋆	Electronic medical records and clinical charts were reviewed. [59]
Was the follow-up long enough for outcomes to occur	⋆	*"All complications were defined as those occurring within 30 days from the date of surgery or discharge, whichever timeframe was longer."* [59]
Adequacy of follow up of cohorts	-	No statement.
**Total score**	⋆ ⋆ ⋆ ⋆ ⋆ (5)	

Table S2.21. NOS assessment [22] – Study ID: Tang et al., 2020 [60]

Criteria for cohort studies	Authors judgement	Support for judgement
Selection		
Representativeness of the exposed cohort	⋆	*"This study was a retrospective review of 390 CD patients undergoing surgical resection from June 2014 to June 2018 at IBD Center, a teaching hospital of Zhejiang University. All the data of CD patients were collected from patients' medical charts in the IBD database. All CD patients were diagnosed depended on accepted criteria [9] […] CD patients with intestinal resection due to failure of medical therapy or developed complications (structuring, penetrating, or malignancy) were included in this study [9]."* [60]
Selection of the non-exposed cohort	⋆	The exposed and non-exposed cohort were obtained from the same database, hospitals and time period: *"Totally, 390 CD patients were enrolled in this study […] For medication history, 85 (21.8%) received IFX within 8 weeks before surgery […]"* [60]
Ascertainment of exposure	⋆	Medical records were reviewed: *"This study was a retrospective review of 390 CD patients undergoing surgical resection from June 2014 to June 2018 at IBD Center, a teaching hospital of Zhejiang University. All the data of CD patients were collected from patients' medical charts in the IBD database."* [60]
Demonstration that outcome of interest was not presented at start of study	-	No statement.
Comparability		
Comparability of cohorts on the basis of the design and analysis: controlled for a critical factor and or other medication	-	Adjusted for preoperative use of anti-TNF by conducting multivariate analysis: *"Multivariate analysis revealed that laparoscopic surgery (OR = 0.398, 95% CI 0.203–0.782, P = 0.008), operative time (OR = 2.105, 95% CI 1.046– 4.238, P = 0.037), and preoperative IFX (OR = 1.951, 95% CI 1.025–3.713, P = 0.042) were independent factors associated with infectious complications in the overall group of CD patients. However, multivariate analysis suggested that ESR level was an independent risk factor associated with infectious complications in patients receiving preoperative IFX (OR = 3.562, 95% CI 1.198–10.595, P = 0.022), and CRP level was an independent risk factor in patients not receiving preoperative IFX (OR = 2.637, 95% CI 1.174–5.923, P = 0.019). Different risk factors were found in CD patients treated with and without preoperative IFX in our study as shown in Tables 4 and 5."* [60]
Comparability of cohorts on the basis of the design and analysis: controlled for additional factor	⋆	Adjusted for preoperative white blood cell count, albumin level, C-reactive protein level, creation of a stoma, laparoscopic surgery, estimated blood loss, operative time and Montreal B classification by conducting multivariate analysis: *"Multivariate*

		analysis revealed that laparoscopic surgery (OR = 0.398, 95% CI 0.203–0.782, P = 0.008), operative time (OR = 2.105, 95% CI 1.046– 4.238, P = 0.037), and preoperative IFX (OR = 1.951, 95% CI 1.025–3.713, P = 0.042) were independent factors associated with infectious complications in the overall group of CD patients. However, multivariate analysis suggested that ESR level was an independent risk factor associated with infectious complications in patients receiving preoperative IFX (OR = 3.562, 95% CI 1.198–10.595, P = 0.022), and CRP level was an independent risk factor in patients not receiving preoperative IFX (OR = 2.637, 95% CI 1.174–5.923, P = 0.019). Different risk factors were found in CD patients treated with and without preoperative IFX in our study as shown in Tables 4 and 5." [60]
Outcome		
Assessment of outcome	⋆	Medical records were reviewed. [60]
Was the follow-up long enough for outcomes to occur	⋆	*"Postoperative complications were defined as those occurring within 30 days from the date of operation including discharge based on the Clavien-Dindo system [10]."* [60]
Adequacy of follow up of cohorts	-	No statement.
**Total score**	⋆ ⋆ ⋆ ⋆ ⋆ ⋆ (6)	

Table S2.22. NOS assessment [22] – Study ID: Tay et al., 2003 [61]

Criteria for cohort studies	Authors judgement	Support for judgement
Selection		
Representativeness of the exposed cohort	⋆	*"A review of all CD patients followed at a tertiary referral Inflammatory Bowel Disease Center between 1998 and 2002 was performed. Patients undergoing their first resection with anastomosis or strictureplasty at our institution were eligible for analysis." [61]*
Selection of the non-exposed cohort	⋆	*"A chart review of identified patients yielded 211 CD patients who underwent abdominal surgery during the study period. Isolated perianal surgeries were excluded. […] Review of the UMB electronic medical record (Cerner Powerchart, Kansas City, MO) and clinic charts provided operative details as well as […] Among patients treated with immunomodulators (azathioprine, 6-mercaptopurine, methotrexate, or infliximab) for >8 weeks before surgery, the rate of IASC was 5.6%, compared with […]"[61]*

Ascertainment of exposure	⋆	*"A chart review of identified patients yielded 211 CD patients who underwent abdominal surgery during the study period. Isolated perianal surgeries were excluded. […] Review of the UMB electronic medical record (Cerner Powerchart, Kansas City, MO) and clinic charts provided operative details as well as […] Among patients treated with immunomodulators (azathioprine, 6-mercaptopurine, methotrexate, or infliximab) for >8 weeks before surgery, the rate of IASC was 5.6%, compared with […]" [61]; "Concomitant medications used by patients during 72 Crohn's disease surgical procedures in the immunomodulator-treated group." [61]*
Demonstration that outcome of interest was not presented at start of study	-	No statement.
Comparability		
Comparability of cohorts on the basis of the design and analysis: controlled for a critical factor and or other medication	⋆	Adjusted for use of steroids by conducting multivariate analysis. *[61]*
Comparability of cohorts on the basis of the design and analysis: controlled for additional factor	⋆	Adjusted for serum albumin level, preoperative abscess/fistula, age and smoking status by conducting multivariate analysis. *[61]*
Exposure		
Assessment of outcome	⋆	Medical records were reviewed. *[61]*
Was the follow-up long enough for outcomes to occur	⋆	*"This study showed a strong inverse correlation between serum albumin level with 30-day postoperative morbidity and mortality." [61]*
Adequacy of follow up of cohorts	-	No statement.
**Total score**	⋆ ⋆ ⋆ ⋆ ⋆ ⋆ ⋆ (7)	

Table S2.23. NOS assessment [22] – Study ID: Tiberi et al., 2020 ***[62]***

Criteria for cohort studies	Authors judgement	Support for judgement
Selection		

Representativeness of the exposed cohort	⋆	*"A review of consecutive CD patients who underwent an ileocolic resection and CC patients treated with right hemicolectomy from January 1, 2008, to June 1, 2019, at Digestive and IBD Surgery Unit, Careggi University Hospital, was performed." [62]*
Selection of the non-exposed cohort	⋆	The exposed and non-exposed cohort were obtained from the same database, hospitals and time period: *"Regarding the preoperative treatment with anti-TNF drugs, only patients who were still in treatment with anti-TNFα in the 12 weeks before surgery have been considered, in line with other studies conducted on the same topic. […] Of the 133 patients with CD, 24.4% had received preoperative treatment with anti-TNF drugs up to 3 months before surgery (Table 1)." [62]*
Ascertainment of exposure	⋆	Medical records were reviewed: *"The data have been collected consulting medical and operating records, and include: age, sex, BMI and nutritional status, exposure to cigarette smoking, familiarity with Crohn's disease, Montreal classification, symptoms experienced before surgery (including extra-intestinal disease manifestations), type of pre- and post-surgical pharmacological and nutritional treatment, type and duration of surgical procedure, length of hospitalization, location of active disease and extention of resected bowel, type of anastomosis, presence of major perioperative complications" [62]*
Demonstration that outcome of interest was not presented at start of study	-	No statements.
Comparability		
Comparability of cohorts on the basis of the design and analysis: controlled for a critical factor and or other medication	-	Not adjusted for critical factor and or other medication.
Comparability of cohorts on the basis of the design and analysis: controlled for additional factor	-	Not adjusted for additional factor.
Outcome		
Assessment of outcome	⋆	Medical records were reviewed.
Was the follow-up long enough for outcomes to occur	⋆	*"Only major complication (Clavien–Dindo III–IV) occurred within 30 days from surgery were considered." [62]*

Adequacy of follow up of cohorts	-	No statement.
**Total score**	⋆ ⋆ ⋆ ⋆ ⋆ (5)	

**Table S2.24.** NOS assessment [22] – Study ID: Uchino et al., 2013 [65]		
**Criteria for cohort studies**	**Authors judgement**	**Support for judgement**
Selection		
Representativeness of the exposed cohort	⋆	*"We prospectively collected and reviewed patients' background and perioperative data following our institutional review board's approval. Consecutive patients with CD who underwent a laparotomy at the Hyogo College of Medicine between January 2008 and December 2011 were included in this study." [65]*
Selection of the non-exposed cohort	⋆	The exposed and non-exposed cohort were obtained from the same database, hospitals and time period: *"We analyzed 405 patients with CD who underwent abdominal surgery. […] Among the patients, 60.0% (243/405) had the penetrating disease type. Preoperative IFX was used in 19.5% (79/405) of the patients. Preoperative IFX was […]"[65]*
Ascertainment of exposure	⋆	Data obtained from prospectively maintained database of Hyogo College of Medicine. *[65]*
Demonstration that outcome of interest was not presented at start of study	-	Overall, 133 patients were classified as having a contaminated wound class III (26 out of 79 patients of the exposed cohort and 107 out of 326 patients of the non-exposed cohort) and 27 patients were classified as having a dirty-infected wound class IV (5 out of 79 patients of the exposed cohort and 22 out of 326 patients of the non-exposed cohort). *[65]*
Comparability	⋆	*We decided to give one star for comparability because some of the important factors were adjusted, others not.*
Comparability of cohorts on the basis of the design and analysis: controlled for a critical factor and or other medication		Adjusted for infliximab administration for incisional SSI by conducting a multivariate analysis: *"The result of the final 3 steps of the multivariate analysis of risk factors for SSIs was restricted to patients with penetrating CD. The data are shown in Table 5. In the stepwise logistic regression model, duration of surgery ≥167 minutes (OR 3.1) and frequencies of surgeries ≥2 (OR 2.6) were selected as independent risk factors for*

		overall SSIs. Ostomy creation (OR 4.3) and proctectomy (OR 3.2) were selected as independent risk factors for incisional SSIs. A serum Alb level <2.9 g/dL (OR 4.5) and proctectomy (OR 3.3) were selected as independent risk factors for organ/space SSIs. A clean-contaminated wound (OR 0.5) was selected as a risk-reducing factor for overall SSIs. Additionally, IFX administration (OR 0.1) was selected as a risk-reducing factor for incisional SSIs […]"[65]
Comparability of cohorts on the basis of the design and analysis: controlled for additional factor		Overall SSI adjusted by conducting multivariate analysis for colonic surgery; duration of surgery >167 minutes; national nosocomial infection surveillance risk index ≥2; ostomy creation; frequency of surgery ≥2; penetrating type and proctectomy. Incisional SSI adjusted by conducting multivariate analysis for colonic surgery; clean-contaminated wound; ostomy creation; duration of surgery >167 min and proctectomy. Organ/ space SSI adjusted by conducting multivariate analysis for immunomodulator administration; duration of surgery >167 min; ostomy creation; preoperative serum albumin <2.9g/dL; postoperative blood sugar ≥200 mg/dL and proctectomy. *[25] "The result of the final 3 steps of the multivariate analysis of risk factors for SSIs was restricted to patients with penetrating CD. The data are shown in Table 5. In the stepwise logistic regression model, duration of surgery ≥167 minutes (OR 3.1) and frequencies of surgeries ≥2 (OR 2.6) were selected as independent risk factors for overall SSIs. Ostomy creation (OR 4.3) and proctectomy (OR 3.2) were selected as independent risk factors for incisional SSIs. A serum Alb level <2.9 g/dL (OR 4.5) and proctectomy (OR 3.3) were selected as independent risk factors for organ/space SSIs. A clean-contaminated wound (OR 0.5) was selected as a risk-reducing factor for overall SSIs. Additionally, IFX administration (OR 0.1) was selected as a risk-reducing factor for incisional SSIs […]"[65]*
Outcome		
Assessment of outcome	⋆	Data obtained from prospectively maintained database of Hyogo College of Medicine. *[65]*
Was the follow-up long enough for outcomes to occur	⋆	*"The criteria for the diagnosis of SSI included an infection that occurred within 30 days after the operation and at least […].[65]*
Adequacy of follow up of cohorts	-	No statement.

Total score	⋆ ⋆ ⋆ ⋆ ⋆ ⋆ (6)	

**Table S2.25.** NOS assessment [22] – Study ID: White et al., 2012 [69]		
**Criteria for cohort studies**	**Authors judgement**	**Support for judgement**
Selection		
Representativeness of the exposed cohort	⋆	*“A prospective database of CD patients requiring abdominal surgery was queried. Consecutive operations done between December 1999 and July 2009 were included in the analysis. All procedures were performed by one surgeon (P.F.) using an open or laparoscopic technique, and included resection, strictureplasty, or both. All patients were included in a hospital-wide standardized fast-track protocol.” [69]*
Selection of the non-exposed cohort	⋆	The exposed and non-exposed cohort were obtained from the same database, hospitals and time period: *“The study group included 338 CD patients who required abdominal surgery during the study period. […]. Biologic therapies included infliximab (n = 38), adalimumab (n = 17), and certolizumab (n = 4).” [69]*
Ascertainment of exposure	⋆	Data obtained by a prospectively maintained database: *“Detailed clinical profiles assessing demographic information and characteristics of the disease and its treatment had been previously prospectively generated by one investigator (P.F.).” [69]*
Demonstration that outcome of interest was not presented at start of study	-	No statements.
Comparability		
Comparability of cohorts on the basis of the design and analysis: controlled for a critical factor and or other medication	-	Not adjusted for critical factor and or other medication.

Comparability of cohorts on the basis of the design and analysis: controlled for additional factor	-	Not adjusted for additional factor.
Outcome		
Assessment of outcome	⋆	Data obtained by a prospectively maintained database: *"Detailed clinical profiles assessing demographic information and characteristics of the disease and its treatment had been previously prospectively generated by one investigator (P.F.)." [69]*
Was the follow-up long enough for outcomes to occur	⋆	*"UR was defined as readmission occurring within 30 days of hospital discharge." [69]*
Adequacy of follow up of cohorts	-	No statement.
**Total score**	⋆ ⋆ ⋆ ⋆ ⋆ (5)	

Table S2.26. NOS assessment [22] – Study ID: Yamamoto et al., 2016 [71]

Criteria for cohort studies	Authors judgement	Support for judgement
Selection		
Representativeness of the exposed cohort	⋆	*"This was a retrospective international multicentre study. In this study, seven IBD referral centres from three countries (Japan, Brazil and Italy) were involved. Our study protocol was approved by the ethics committee of each institution involved. […] Patients who underwent ileocolonic resection (by open or laparoscopic approach) with primary anastomosis for active CD between January 2008 and January 2013 were included. Patients who underwent concomitant intestinal resection or strictureplasty for small bowel or colorectal CD were included."* [71]
Selection of the non-exposed cohort	⋆	The exposed and non-exposed cohort were obtained from the same database, hospitals and time period: *"A total of 231 consecutive patients were included in this study. […] Within eight weeks before surgery, 65 patients (28%) were receiving immunosuppressive drugs (azathioprine or 6-mercaptopurine). Of the patients, 79 (34%) received biologic agents (infliximab for 55 patients, adalimumab for 23 patients, both for one patient) within eight weeks before surgery."* [71]
Ascertainment of exposure	⋆	Data obtained from a prospectively maintained database in each institution: *"The methodological features of a retrospective multicentre and observational analysis, without a*

		fixed perioperative management protocol, may have led to some bias, even if the database in each institution was prospectively maintained." [71]
Demonstration that outcome of interest was not presented at start of study	-	Overall, 99 patients presented with perforating disease at surgery, 48% of the exposed cohort (38 out of 79 patients) and 40% of the non-exposed cohort (61 out of 152 patients): *"The indication for surgery was perforating disease in 99 patients (43%) […]"* [71]
Comparability		
Comparability of cohorts on the basis of the design and analysis: controlled for a critical factor and or other medication	⋆	Adjusted for the use of preoperative steroids; immunosuppressants and biologicals by conducting a multivariate analysis: *"In multivariate analysis (Table 6), only blood transfusion was a significant independent risk factor for overall complications (odds ratio [OR] 3.02, 95% confidence interval [CI] 1.21–7.52; P=0.02). Perforating disease was the only significant risk factor for intraabdominal sepsis (OR 2.67, 95% CI 1.04–6.86; P=0.04). Further, previous resection was the only significant risk factor for anastomotic leak (OR 2.87, 95% CI 1.01–8.18; P=0.048)."* [71]
Comparability of cohorts on the basis of the design and analysis: controlled for additional factor	⋆	Adjusted for age at surgery; gender; behaviour of CD; smoking; previous resection; blood transfusion; surgical procedure and type of anastomosis by conducting a multivariate analysis: *"In multivariate analysis (Table 6), only blood transfusion was a significant independent risk factor for overall complications (odds ratio [OR] 3.02, 95% confidence interval [CI] 1.21–7.52; P=0.02). Perforating disease was the only significant risk factor for intraabdominal sepsis (OR 2.67, 95% CI 1.04–6.86; P=0.04). Further, previous resection was the only significant risk factor for anastomotic leak (OR 2.87, 95% CI 1.01–8.18; P=0.048)."* [71]
Outcome		
Assessment of outcome	⋆	Data obtained from a prospectively maintained database in each institution. [71]
Was the follow-up long enough for outcomes to occur	⋆	*"Postoperative complications occurring within 30 days after surgery were analysed."* [71]
Adequacy of follow up of cohorts	⋆	*"Patients with insufficient data for analysis were also excluded."* [71]
**Total score**	⋆ ⋆ ⋆ ⋆ ⋆ ⋆ ⋆ ⋆ (8)	

**Table S2.27.** NOS assessment [22] – Study ID: Yu et al., 2019 [72]		
**Criteria for cohort studies**	**Authors judgement**	**Support for judgement**
Selection		
Representativeness of the exposed cohort	⋆	*"This retrospective study evaluated patients who underwent intestinal surgery for CD at the Asan Medical Center, Seoul, Korea, between January 2006 and December 2015. Patients were included if they had undergone bowel resection, with or without anastomosis, and strictureplasty for CD during the study period. Only patients aged 16 years and older at the time of surgery were included."* [72]
Selection of the non-exposed cohort	⋆	The exposed and non-exposed cohort were obtained from the same database, hospitals and time period: *"Of the 461 patients in the medication group, 254 (55.1%) had received immunomodulators alone and 116 (25.2%) were on combined medication. Of the latter, 54 (46.6%) had received immunomodulators plus anti-TNF-α agents, 42 (36.2%) immunomodulators plus steroids, 8 (1.7%) anti-TNF-α agents plus steroids, and 12 (2.6%) immunomodulators plus anti-TNF-α agents plus steroids (Fig. 1) […]"* [72]
Ascertainment of exposure	⋆	Medical records were reviewed: *"The medical records of the patients included in the cohort were reviewed."* [72]
Demonstration that outcome of interest was not presented at start of study	-	Overall, 188 patients underwent surgery for abscess, 234 underwent surgery for fistula and 75 patients underwent surgery for peritonitis. [72]
Comparability		
Comparability of cohorts on the basis of the design and analysis: controlled for a critical factor and or other medication	-	Not adjusted for critical factor and or other medication by conducting multivariate analysis, only univariable and multivariable analysis were performed. [72]
Comparability of cohorts on the basis of the design and analysis: controlled for additional factor	-	Not adjusted for additional factor by conducting multivariate analysis, only univariable and multivariable analysis were performed. [72]
Outcome		
Assessment of outcome	⋆	Medical records were reviewed. [72]

Was the follow-up long enough for outcomes to occur	⋆	*"[…] postoperative complications within the 30 days postoperation including […] Postoperative complications were defined as grade 2A or higher on the Clavien-Dindo classification and the 30-day postoperative complications"* [72]
Adequacy of follow up of cohorts	-	No statement.
**Total score**	⋆ ⋆ ⋆ ⋆ ⋆ (5)	

**Table S2.28.** NOS assessment [22] – Study ID: Zhu et al., 2020 [73]		
**Criteria for cohort studies**	**Authors judgement**	**Support for judgement**
Selection		
Representativeness of the exposed cohort	⋆	*"Institutional review board approval was obtained to query the IBD Database of Jinling Hospital. Data of consecutive surgeries for CD were collected from the database over a 7-year period (January 2011 to December 2017). All of the surgeries were performed on patients diagnosed with stricturing or penetrating CD."* [73]
Selection of the non-exposed cohort	⋆	The exposed and non-exposed cohort were obtained from the same database, hospitals and time period. [73]
Ascertainment of exposure	-	No statement.
Demonstration that outcome of interest was not presented at start of study	-	Overall, 128 patients presented with a preoperative intraabdominal abscess. [73]
Comparability		
Comparability of cohorts on the basis of the design and analysis: controlled for a critical factor and or other medication	⋆	Adjusted for critical factor and or other medication by conducting multivariate analysis. [73]

Comparability of cohorts on the basis of the design and analysis: controlled for additional factor	⋆	Adjusted for additional factor by conducting multivariate analysis. [73]
Outcome		
Assessment of outcome	-	No statement
Was the follow-up long enough for outcomes to occur	⋆	*"The primary outcome was occurrence of IASCs within 30 days after surgery or before discharge."* [73]
Adequacy of follow up of cohorts	⋆	*"No patient had missing outcome data."* [73]
**Total score**	⋆ ⋆ ⋆ ⋆ ⋆ ⋆ (6)	

Table S2.29. NOS assessment [22] – Study ID: Bregnbak et al., 2012 [27]

Criteria for cohort studies	Authors judgement	Support for judgement
Selection		
Representativeness of the exposed cohort	⋆	*"Retrospective data was gathered from a study population treated at Hvidovre Hospital (HH) in the period of January 1, 2005 to September 23, 2010. The cohort of patients studied was initially found through a database query with the Danish National Health Register. This database contains all contacts and hospitalizations within the Danish healthcare system, including primary diagnosis and surgical procedures."* [27]
Selection of the non-exposed cohort	⋆	The exposed and non-exposed cohort were obtained from the same database, hospital and time period: *"The patients were grouped into 2 separate cohorts based on use or no use of IFX within 90 days prior to primary surgery. Among the 71 patients analyzed, 20 patients received IFX prior to surgery […]"* [27]
Ascertainment of exposure	⋆	The Danish National Health Register and its database contain the information about the exposure, patient records were systematically scrutinized. [27]

Demonstration that outcome of interest was not presented at start of study	-	No statement.
Comparability		
Comparability of cohorts on the basis of the design and analysis: controlled for a critical factor and or other medication	-	Not adjusted for critical factor and or other medication.
Comparability of cohorts on the basis of the design and analysis: controlled for additional factor	-	Not adjusted for additional factor.
Outcome		
Assessment of outcome	⋆	Medical records were examined: *"These 87 candidate patient records were systematically scrutinized."* [27]
Was the follow-up long enough for outcomes to occur	⋆	*"The primary outcome measure was complications within 30 days after primary surgery […]"* [27]
Adequacy of follow up of cohorts	-	No statement.
**Total score**	⋆ ⋆ ⋆ ⋆ ⋆ (5)	

Table S2.30. NOS assessment [22] – Study ID: Ferrante et al., 2009 [33]

Criteria for cohort studies	Authors judgement	Support for judgement
Selection		
Representativeness of the exposed cohort	⋆	*"Patients were identified through the surgical database and diagnosis of UC or IBDU was confirmed after thorough review of medical and pathological records."* [33]
Selection of the non-exposed cohort	⋆	The exposed and non-exposed cohort were obtained from the same database, hospitals and time period: *"We identified 41 patients who received IFX at some*

		timepoint prior to surgery. Twenty-two of these patients received IFX within 12 weeks prior to primary surgery […].” [33]
Ascertainment of exposure	⋆	Medical records were examined: *“Clinical charts of all patients were reviewed to trace clinical characteristics including gender, age at diagnosis […] medical therapy within 12 weeks prior to primary surgery, indication […]”* [33]; *“We identified 41 patients who received IFX at some timepoint prior to surgery. Twenty-two of these patients received IFX within 12 weeks prior to primary surgery […].”* [33]
Demonstration that outcome of interest was not presented at start of study	-	No statement.
Comparability		
Comparability of cohorts on the basis of the design and analysis: controlled for a critical factor and or other medication	-	Not adjusted for critical factor and or other medication by conducting a multivariate analysis, only a univariate analysis was performed. [33]
Comparability of cohorts on the basis of the design and analysis: controlled for additional factor	-	Not adjusted for additional factor by conducting a multivariate analysis, only a univariate analysis was performed. [33]
Outcome		
Assessment of outcome	⋆	Clinical charts of all patients were reviewed. [33]
Was the follow-up long enough for outcomes to occur	⋆	*“Short-term postoperative complications were recorded within the first 30 days after primary surgery […]”* [33]
Adequacy of follow up of cohorts	-	No statement.
**Total score**	⋆ ⋆ ⋆ ⋆ ⋆ (5)	

Table S2.31. NOS assessment [22] – Study ID: Gainsbury et al., 2011 [34]

**Criteria for cohort studies**	**Authors judgement**	**Support for judgement**
Selection		
Representativeness of the exposed cohort	⋆	*“Data were collected from an IRB-approved IPAA Registry at Boston University Medical Center. We identified 81 consecutive UC patients who underwent IPAA between September 2005 and May 2009 by a single surgeon (J.M.B.). […] Patients with a diagnosis of UC registered in the IPAA database who underwent IPAA at Boston University Medical Center between September 2005 and May 2009 were included in this study […]”* [34]
Selection of the non-exposed cohort	⋆	The exposed and non-exposed cohort were obtained from the same hospitals and time period: *“Of the 81 subjects, 29 had received IFX treatment within 12 weeks of the first stage of their IPAA surgery. Fifty-two control subjects remained as the non-IFX group.”* [34]
Ascertainment of exposure	⋆	Medical records were reviewed: *“Medical records of all included subjects were retrospectively reviewed.”* [34]; *“A total of 81 patients meeting inclusion criteria underwent IPAA during the study period. Twenty-nine of those were treated with IFX within the predetermined 12-week period prior to surgery and 52 remained as non-IFX control subjects.”* [34]
Demonstration that outcome of interest was not presented at start of study	-	No statement.
Comparability		
Comparability of cohorts on the basis of the design and analysis: controlled for a critical factor and or other medication	⋆	Adjusted for the use of any steroids; 6-MP and methotrexate by conducting a multivariate analysis. [34]
Comparability of cohorts on the basis of the design and analysis: controlled for additional factor	⋆	Adjusted for laparoscopic colectomy; BMI and failed medical therapy by conducting a multivariate analysis. [34]
Outcome		
Assessment of outcome	⋆	Medical records were reviewed. [34]

Was the follow-up long enough for outcomes to occur	⋆	*"Short-term postoperative complications were defined as having occurred between the first-stage IPAA surgery until up to within 30 days after the last-stage IPAA surgery, the closure of the diverting loop ileostomy."* [34]
Adequacy of follow up of cohorts	-	No statement.
**Total score**	⋆ ⋆ ⋆ ⋆ ⋆ ⋆ ⋆ (7)	

Table S2.32. NOS assessment [22] – Study ID: Gu et al., 2013 [35]

Criteria for cohort studies	Authors judgement	Support for judgement
Selection		
Representativeness of the exposed cohort	⋆	*"After institutional review board approval, all adult patients who underwent STC/EI for UC or IC during 2006 through 2010 were identified by International Classification of Diseases, Ninth Revision diagnosis and Current Procedural Terminology-4 procedure codes. Patients who underwent subsequent CP/IPAA were identified from an institutional review board-approved, prospectively maintained pouch database. Patients who underwent TPC/IPAA during the same period were also identified from the pouch database. Only patients operated on for medically refractory colitis were included."* [35]
Selection of the non-exposed cohort	⋆	The exposed and non-exposed cohort were obtained from the same database, hospitals and time period: *"Of the 181 patients who underwent initial TPC/IPAA for medically refractory UC/IC over the time period, 25 patients (13.8%) received anti-TNF-α agents, including IFX (n = 23) and adalimumab (n = 2)."* [35]
Ascertainment of exposure	⋆	Data obtained from a prospectively maintained pouch database. [35]
Demonstration that outcome of interest was not presented at start of study	-	No statement.

Comparability		
Comparability of cohorts on the basis of the design and analysis: controlled for a critical factor and or other medication	-	Not adjusted for critical factor and or other medication.
Comparability of cohorts on the basis of the design and analysis: controlled for additional factor	-	Not adjusted for additional factor.
Outcome		
Assessment of outcome	⋆	Data obtained from a prospectively maintained pouch database. [35]
Was the follow-up long enough for outcomes to occur	⋆	*"Demographics, perioperative data, 30-day and cumulative 1-year postoperative complication rates, and long-term pouch functional outcomes were retrospectively collected and compared between the 2 groups […]"* [35]
Adequacy of follow up of cohorts	-	No statement.
**Total score**	⋆ ⋆ ⋆ ⋆ ⋆ (5)	

Table S2.33. NOS assessment [22] – Study ID: Kim et al., 2020 [39]

Criteria for cohort studies	Authors judgement	Support for judgement
Selection		
Representativeness of the exposed cohort	⋆	*"A prospectively maintained database from single tertiary hospital, evaluating the role of clinical, serologic and genetic markers with clinical phenotypes in IBD was queried for data on affected individuals with medically refractory UC undergoing first abdominal major surgery between January 2007 and June 2017. Patients requiring surgery for chronic disease with cancer, CD, indeterminate colitis (IC), or those treated with ustekinumab prior to surgery were excluded."* [39]
Selection of the non-exposed cohort	⋆	*"Over the 11-year period, 285 patients had an abdominal colectomy by one surgeon. Total cohort data was tabulated (Table 1). A total of 25 procedures were identified as meeting entry criteria with exposure to vedolizumab. We used 1:3 matching based on age, gender*

		and number of stages of patients treated with vedolizumab, of 77 patients with non-biologics, only 54 patients were eligible for matching condition. Seventy-four controls unexposed to vedolizumab yet who received anti-TNF therapy were matched 1:3. Anti-TNF agents used included infliximab (n = 48), adalimumab (n = 26)." [39]
Ascertainment of exposure	⋆	Data obtained from a prospectively maintained database and medical records were reviewed: *"A prospectively maintained database from single tertiary hospital, evaluating the role of clinical, serologic and genetic markers with clinical phenotypes in IBD was queried for data on affected individuals with medically refractory UC undergoing first abdominal major surgery between January 2007 and June 2017. Patients requiring surgery for chronic disease with cancer, CD, indeterminate colitis (IC), or those treated with ustekinumab prior to surgery were excluded."* [39]; *"Over the 11-year period, 285 patients had an abdominal colectomy by one surgeon. Total cohort data was tabulated (Table 1). A total of 25 procedures were identified as meeting entry criteria with exposure to vedolizumab. We used 1:3 matching based on age, gender and number of stages of patients treated with vedolizumab, of 77 patients with non-biologics, only 54 patients were eligible for matching condition. Seventy-four controls unexposed to vedolizumab yet who received anti-TNF therapy were matched 1:3. Anti-TNF agents used included infliximab (n = 48), adalimumab (n = 26)."* [39]
Demonstration that outcome of interest was not presented at start of study	-	No statement.
Comparability		
Comparability of cohorts on the basis of the design and analysis: controlled for a critical factor and or other medication	⋆	Adjusted for use of anti-TNF; 6-MP or methotrexate by conducting multivariate analysis. [39]
Comparability of cohorts on the basis of the design and analysis: controlled for additional factor	⋆	Matched based on age, gender and surgical procedure: *"Those individuals with documented preoperative exposure to more than three times of vedolizumab within 12 weeks prior to surgery were selected and then matched 3:3:1 into 3 preoperative treatment groups (no biologics, anti-TNF treated, vedolizumab treated) based on age, gender and surgical procedure (abdominal colectomy with Brooke ileostomy or abdominal colectomy with ileal pouch-anal anastomosis (IPAA) with loop ileostomy)."* [39]<br>Adjusted for acute disease activity and laparoscopy by conducting multivariate analysis. [39]

Exposure		
Assessment of outcome	⋆	Data obtained from a prospectively maintained database and medical records were reviewed. [39]
Was the follow-up long enough for outcomes to occur	⋆	*"Postoperative complications were recorded within 30 days of the index operation."* [39]
Adequacy of follow up of cohorts	-	No statement.
**Total score**	⋆ ⋆ ⋆ ⋆ ⋆ ⋆ ⋆ (7)	

Table S2.34. NOS assessment [22] – Study ID: Nelson et al., 2014 [51]

Criteria for cohort studies	Authors judgement	Support for judgement
Selection		
Representativeness of the exposed cohort	⋆	*"This retrospective cohort study was approved by the Institutional Review Board of the University of Chicago. Using ICD codes 45.7 to 45.83 and 556.0 to 556.9, we identified 978 patients with UC who underwent colectomy at the University of Chicago Hospitals from October 1, 2006 to October 1, 2012. Through a preliminary chart review, the database was limited to the following inclusion criteria: (1) a confirmed diagnosis of UC by histology and endoscopy, (2) colectomy for acute severe UC refractory to medical therapy, (3) treatment with IV corticosteroids, IFX, or cyclosporine during the hospitalization leading to colectomy."* [51]
Selection of the non-exposed cohort	⋆	The exposed and non-exposed cohort were obtained from the same database, hospitals and time period: *"Patients were grouped according to the medical rescue therapy used before colectomy and charts were reviewed retrospectively."* [51]
Ascertainment of exposure	⋆	Data obtained by codes from a database of University of Chicago Hospitals and medical records were reviewed. [51]
Demonstration that outcome of interest was not presented at start of study	-	Patients from the exposed and non-exposed cohort presented with an increased median C-reactive protein at surgery (the exposed cohort presented with a C-

		reactive protein level of 64 mg/L (0-371), the non-exposed cohort with 38 mg/L (0-242) and 35 mg/L (0-144). [51]
Comparability		
Comparability of cohorts on the basis of the design and analysis: controlled for a critical factor and or other medication	-	Not adjusted for a critical factor and or other medication by conducting multivariate analysis, only univariable and multivariable analysis were performed. [51]
Comparability of cohorts on the basis of the design and analysis: controlled for additional factor	-	Not adjusted for additional factor by conducting multivariate analysis, only univariable and multivariable analysis were performed. [51]
Outcome		
Assessment of outcome	⋆	Data obtained by codes from a database of University of Chicago Hospitals and medical records were reviewed. [51]
Was the follow-up long enough for outcomes to occur	⋆	*"Consistent with previous studies on postoperative complications after IFX therapy, analysis was focused on short-term complications occurring within 30 days of colectomy."* [51]
Adequacy of follow up of cohorts	-	No statement.
**Total score**	⋆ ⋆ ⋆ ⋆ ⋆ (5)	

Table S2.35. NOS assessment [22] – Study ID: Nørgård et al., 2012 [52]

Criteria for cohort studies	Authors judgement	Support for judgement
Selection		
Representativeness of the exposed cohort	⋆	*"In Denmark (population approximately 5.5 million people), all citizens have free access to a tax supported health care system. Its uniform organisation allowed us to use a population-based study design. In addition, the availability of nationwide Danish registries made it possible to assess data from Statistic Denmark on (i) patients with UC; (ii) treatment with anti-TNF-a agents; (iii) therapeutic drug use before date of first operation of*

		UC and (iv) post-operative complications (death, reoperation, anastomosis leakage, percutaneous abscess drainage and bacteremia) […]" [52]
Selection of the non-exposed cohort	⋆	The exposed and non-exposed cohort were obtained from the same database, hospitals and time period: *"During the study period of 8 years, 1226 colectomies were registered in UC patients aged ≥15 years. The exposed cohort comprised 199 (16.2%) patients treated with anti-TNF-a agents within 12 weeks before surgery, and the remaining were not treated with anti-TNF-a agent within 12 weeks before surgery, i.e. unexposed cohort 1 = 1027 (83.8%)."* [52]
Ascertainment of exposure	⋆	Data obtained from the National Patient Registry by codes and out-patient drug prescriptions from a nationwide prescription database maintained by Danish Medicine Agency, as well by codes. [52]
Demonstration that outcome of interest was not presented at start of study	-	No statement.
Comparability		
Comparability of cohorts on the basis of the design and analysis: controlled for a critical factor and or other medication	-	Odds ratio adjusted for use of steroids within 4 weeks before surgery: *"We used logistic regression analyses to compute relative risk estimates for adverse post-operative outcomes (reoperations, anastomosis leakage, intra-abdominal abscess, bacteremia and death within 30 days and 60 days after the first UC operation) associated with anti-TNF-a agent therapy pre-operatively, […] adjusted for potential confounders. Adjustment was made for age (years 15–25 as reference, 26–55, >55), gender (females as reference), comorbidity (Charlson Index 0 as reference, Charlson Index 1–2, Charlson Index >2), calendar period (2003– 2004 as reference, 2005–2006, 2007–2008, 2009–2010), duration of UC (<5 years as reference, ≥5 years), use of steroids within 4 weeks before surgery (no as reference, yes) and number of inpatient days at hospital within 4 weeks before surgery (<2 weeks as reference, ≥2 weeks) in a logistic regression model. The purpose of including use of steroids and number of inpatient days at hospital within 4 weeks before surgery was to counter in a possible impact of disease activity."* [52]
Comparability of cohorts on the basis of the design and analysis: controlled for additional factor	-	Odds ratio adjusted for age; gender; comorbidity; calendar period; duration of UC and number of inpatient days at hospital within 4 weeks before surgery: *"We used logistic regression analyses to compute relative risk estimates for adverse post-operative outcomes (reoperations, anastomosis leakage, intra-abdominal abscess, bacteremia and death within 30 days and 60 days after the first UC operation) associated with anti-TNF-a agent therapy pre-operatively, […] adjusted for potential confounders. Adjustment was*

		made for age (years 15–25 as reference, 26–55, >55), gender (females as reference), comorbidity (Charlson Index 0 as reference, Charlson Index 1–2, Charlson Index >2), calendar period (2003– 2004 as reference, 2005–2006, 2007–2008, 2009–2010), duration of UC (<5 years as reference, ≥5 years), use of steroids within 4 weeks before surgery (no as reference, yes) and number of inpatient days at hospital within 4 weeks before surgery (<2 weeks as reference, ≥2 weeks) in a logistic regression model. The purpose of including use of steroids and number of inpatient days at hospital within 4 weeks before surgery was to counter in a possible impact of disease activity." [52]
Outcome		
Assessment of outcome	⋆	Data obtained by codes from the National Patient Registry. [52]
Was the follow-up long enough for outcomes to occur	⋆	*"We evaluated all outcomes occurring within 30 and 60 days after UC colectomy."* [52]
Adequacy of follow up of cohorts	⋆	*"[…] only patients available for full follow-up were included."* [52]
**Total score**	⋆ ⋆ ⋆ ⋆ ⋆ ⋆ (6)	

Table S2.36. NOS assessment [22] – Study ID: Selvasekar et al., 2007 [56]

Criteria for cohort studies	Authors judgement	Support for judgement
Selection		
Representativeness of the exposed cohort	⋆	*"This study was approved by the Institutional Review Board of the Mayo Foundation. Between 2002 and 2005, consecutive patients treated preoperatively with IFX at other institutions or at the Mayo Clinic, Rochester, who ultimately underwent IPAA, were identified from a prospectively maintained institutional procedural database."* [56]
Selection of the non-exposed cohort	⋆	The exposed and non-exposed cohort were obtained from the same database, hospitals and time period: *"This study was approved by the Institutional Review Board of the Mayo Foundation. Between 2002 and 2005, consecutive patients treated preoperatively with IFX at other institutions or at the Mayo Clinic, Rochester, who ultimately underwent IPAA, were identified from a prospectively maintained institutional*

		procedural database. This group was compared with consecutive patients who underwent IPAA without earlier exposure to IFX therapy between 2003 and 2005." [56]
Ascertainment of exposure	⋆	Data obtained from a prospectively maintained database, all charts of patients were reviewed and medical records were abstracted: *"All charts of identified patients were reviewed retrospectively. Medical records were abstracted for patient […]"* [56]
Demonstration that outcome of interest was not presented at start of study	-	No statement.
Comparability		
Comparability of cohorts on the basis of the design and analysis: controlled for a critical factor and or other medication	⋆	Adjusted for the use of IFX; steroid (high dose, moderate dose, low dose) and AZA by conducting multivariate analysis. [56]
Comparability of cohorts on the basis of the design and analysis: controlled for additional factor	⋆	Adjusted for age and colitis severity by conducting multivariate analysis. [56]
Outcome		
Assessment of outcome	⋆	Data obtained from a prospectively maintained database, all charts of patients were reviewed and medical records were abstracted. [56]
Was the follow-up long enough for outcomes to occur	⋆	*"Postoperative short-term complications of IPAA within the first 30 days were recorded."* [56]
Adequacy of follow up of cohorts	-	No statement.
**Total score**	⋆ ⋆ ⋆ ⋆ ⋆ ⋆ ⋆ (7)	

**Table S2.37.** NOS assessment [22] – Study ID: Uchino et al., 2013 [66]		
**Criteria for cohort studies**	**Authors judgement**	**Support for judgement**
Selection		

Representativeness of the exposed cohort	⋆	*"UC patients who underwent laparotomy at Hyogo College of Medicine between January 2010 and September 2012 were included in this study."* [66]
Selection of the non-exposed cohort	⋆	The exposed and non-exposed cohort were obtained from the same database, hospitals and time period: *"A total of 196 patients who underwent colectomy were analyzed. Patients' demographics and clinical characteristics are shown in Table 1. Overall, 22/196 (11.2 %) patients were on IFX before surgery, and 53/196 (27.0 %) patients […]"* [66]
Ascertainment of exposure	⋆	Data obtained from a prospectively maintained database: *"[…] analysis of prospectively collected data […]"* [66]
Demonstration that outcome of interest was not presented at start of study	-	Overall, 175 patients were classified as having a contaminated wound class III (21 out of 22 patients of the exposed cohort and 155 out of 174 patients of the non-exposed cohort) and 21 patients were classified as having a dirty-infected wound class IV (1 out of 22 patients of the exposed cohort and 20 out of 174 patients of the non-exposed cohort). [66]
Comparability		
Comparability of cohorts on the basis of the design and analysis: controlled for a critical factor and or other medication	⋆	Adjusted by conducting multivariate analysis for overall SSI (IFX administration and preoperative prednisolone dose ≥0.2 mg/kg/day); incisional SSI (IFX administration); organ/ space SSI (immunomodulator administration and preoperative prednisolone dose ≥0.2 mg/kg/day): *"In addition, the following three factors were included in the multivariate logistic regression analysis: duration from onset of UC ≥6.3 years, IFX administration, and perioperative blood transfusion. On the other hand, severe/fulminant disease activity, preoperative PSL dose ≥0.2 mg/kg/day, urgent/emergent surgery, […] complications. IFX administration was not shown to be an independent risk factor for any complications (Table 5)."* [66]
Comparability of cohorts on the basis of the design and analysis: controlled for additional factor	⋆	Adjusted by conducting multivariate analysis for overall SSI (national nosocomial infection surveillance risk index ≥2; pancolitis; disease activity = severe or fulminant; preoperative hospital stay > 6 days); incisional SSI (national nosocomial infection surveillance risk index ≥2; duration from onset of UC ≥6.3 years; perioperative blood transfusion and ASA score ≥ 3); organ/ space SSI (contaminated wound; preoperative hospital stay > 6 days; national nosocomial infection surveillance risk index ≥2; disease activity = severe or fulminant and urgent or emergent surgery); total infectious complication (intraoperative blood loss ≥200 ml; contaminated wound; disease activity = severe or fulminant;

		pancolitis; male gender; age at surgery ≥ 43 years; urgent or emergent surgery and postoperative blood sugar ≥200 mg/dl): *"In addition, the following three factors were included in the multivariate logistic regression analysis: duration from onset of UC ≥6.3 years, IFX administration, and perioperative blood transfusion. On the other hand, severe/fulminant disease activity, preoperative PSL dose ≥0.2 mg/kg/day, urgent/emergent surgery, […] complications. IFX administration was not shown to be an independent risk factor for any complications (Table 5)."* [66]
Outcome		
Assessment of outcome	⋆	Data obtained from a prospectively maintained database. [66]
Was the follow-up long enough for outcomes to occur	⋆	*"The criteria for a diagnosis of SSI were an infection that occurred within 30 days after the operation and at least one of the following: (1) purulent discharge from the incision […]"* [66]
Adequacy of follow up of cohorts	-	No statement.
**Total score**	⋆ ⋆ ⋆ ⋆ ⋆ ⋆ ⋆ (7)	

Table S2.38. NOS assessment [22] – Study ID: Uchino et al., 2015 [64]

Criteria for cohort studies	Authors judgement	Support for judgement
Selection		
Representativeness of the exposed cohort	⋆	*"We prospectively obtained surveillance data that included the patient's background, characteristics of UC, treatment for UC, surgical details and SSI starting from April 2006 at the Department of Inflammatory Bowel Disease at Hyogo College of Medicine, and we reviewed these data for a retrospective evaluation. Only those patients with a confirmed diagnosis of UC by endoscopy and histology were included in the initial selection. Patients who were surgically treated with an ileal-pouch anal anastomosis (IPAA) procedure as an initial surgery were included in this study."* [64]
Selection of the non-exposed cohort	⋆	The exposed and non-exposed cohort were obtained from the same database, hospitals and time period. [64]
Ascertainment of exposure	⋆	Data obtained from a prospectively maintained database of Hyogo College of Medicine and records were reviewed: *"[…] Department of Inflammatory Bowel Disease at Hyogo College of Medicine, and we reviewed these data for a retrospective evaluation. […]*

		immunomodulator administration (thiopurines, which include AZA and 6-mercaptopurine (6-MP), or CNIs, which include tacrolimus and CyA), BIO administration, immunosuppressive therapy with single, double or triple agents, pre-operative prednisolone (PSL) at a dose of ≥ 18 mg, total administered PSL dose of ≥ 12,000 mg […]" [64]
Demonstration that outcome of interest was not presented at start of study	-	No statement.
Comparability		
Comparability of cohorts on the basis of the design and analysis: controlled for a critical factor and or other medication	⋆	Adjusted by conducting multivariate analysis for overall SSI (use of prednisolone; use of biologic drug; total administered PSL dose of ≥ 12,000 mg and immunosuppressive therapy with double or triple agents): *"In addition, the following 12 factors were included in the multivariate logistic regression analysis: BMI ≥ 25 kg/m 2, disease severity ≥ severe, PSL use, biologic use, total administered PSL dose of ≥ 12,000 mg, immunosuppressive therapy with double or triple agents, urgent surgery, intra-operative bleeding ≥ 232 ml, intra-operative transfusion, ASA score ≥ 3 and BS level ≥ 180 mg/dl."* [64];<br><br>Adjusted by conducting multivariate analysis for incisional SSI (use of prednisolone; total given prednisolone dose 12,000 mg; biological use and immunosuppressive therapy with single/double/triple agents): *"For INC SSI, only PSL use was shown to be a significant risk factor. In addition, the following 10 factors were included in the multivariate logistic regression analysis: duration of UC ≥ 108 months, PSL use, biologic use, total administered PSL dose ≥ 12,000 mg, immunosuppressive therapy with single, double or triple agents, urgent surgery, intra-operative transfusion and ASA score ≥ 3."* [64];<br><br>Adjusted by conducting multivariate analysis for organ/ space (use of prednisolone and AZA/6-MP): *"In O/S SSI, no significant risk factors were found in the univariate analysis. The following 6 factors were included in the multivariate logistic regression analysis: disease severity ≥ severe, PSL use, AZA/6-MP use, intra-operative bleeding ≥ 232 ml, ASA score ≥ 3 and BS level ≥ 180 mg/dl."* [64]
Comparability of cohorts on the basis of the design and analysis: controlled for additional factor	⋆	Adjusted by conducting multivariate analysis for overall SSI (adjusted for BMI ≥ 25 kg/m^2; disease severity; urgent surgery; intra-operative bleeding ≥ 232 ml; intra-operative transfusion; ASA score ≥ 3 and blood sugar level ≥ 180 mg/dl): *"In addition, the following 12 factors were included in the multivariate logistic regression analysis: BMI ≥ 25 kg/m 2, disease severity ≥ severe, PSL use, biologic use, total administered PSL dose of ≥*

		12,000 mg, immunosuppressive therapy with double or triple agents, urgent surgery, intra-operative bleeding ≥ 232 ml, intra-operative transfusion, ASA score ≥ 3 and BS level ≥ 180 mg/dl." [64];<br><br>Adjusted by conducting multivariate analysis for incisional SSI (duration of UC ≥108 months; urgent surgery; intra-operative transfusion and ASA score ≥3): *"For INC SSI, only PSL use was shown to be a significant risk factor. In addition, the following 10 factors were included in the multivariate logistic regression analysis: duration of UC ≥ 108 months, PSL use, biologic use, total administered PSL dose ≥ 12,000 mg, immunosuppressive therapy with single, double or triple agents, urgent surgery, intra-operative transfusion and ASA score ≥ 3."* [64];<br><br>Adjusted by conducting multivariate analysis for organ/ space SSI (disease severity; intra-operative bleeding ≥ 232 ml; ASA score ≥ 3 and blood sugar level ≥ 180 mg/dl): *"In O/S SSI, no significant risk factors were found in the univariate analysis. The following 6 factors were included in the multivariate logistic regression analysis: disease severity ≥ severe, PSL use, AZA/6-MP use, intra-operative bleeding ≥ 232 ml, ASA score ≥ 3 and BS level ≥ 180 mg/dl."* [64];
Outcome		
Assessment of outcome	⋆	Data obtained from a prospectively maintained database of Hyogo College of Medicine and records were reviewed. [64]
Was the follow-up long enough for outcomes to occur	⋆	*"The criterion for a diagnosis of SSI was an infection that occurred within 30 days after the operation."* [64]
Adequacy of follow up of cohorts	-	No statement. [64]
**Total score**	⋆ ⋆ ⋆ ⋆ ⋆ ⋆ ⋆ (7)	

Table S2.39. NOS assessment [22] – Study ID: Uchino et al., 2019 [63]

Criteria for cohort studies	Authors judgement	Support for judgement
Selection		
Representativeness of the exposed cohort	⋆	*"We prospectively obtained surveillance data that included the patient's background, characteristics of UC, treatment for UC, surgical details, and SSI starting from April 2006 at the Department of Inflammatory Bowel Disease at Hyogo College of Medicine, and we reviewed these data for a retrospective evaluation. Only patients with a confirmed diagnosis of UC by endoscopy and histology were included in the initial selection. Patients who were surgically treated with total colectomy and ileostomy, total proctocolectomy (TPC) with end-ileostomy, or ileal-pouch anal anastomosis (IPAA) with or without ileostomy as an initial surgery were included in this study."* [63]
Selection of the non-exposed cohort	⋆	The exposed and non-exposed cohort were obtained from the same database, hospitals and time period: *"A total of 301 patients with UC were surgically treated during the study period. The patient demographics and characteristics are shown in Table 1. PSL, CNIs, and anti-TNF-α antibodies were administered to 117/301 (38.9%), 119/301 (39.5%), and 146/301 (48.5%) patients, respectively. Among patients treated with anti-TNF-α antibodies, 124 patients received IFX, 53 patients received ADA, and 6 patients received GOL."* [63]
Ascertainment of exposure	⋆	Data obtained from a prospectively maintained database of Hyogo College of Medicine and records were reviewed retrospectively: *"[…] at the Department of Inflammatory Bowel Disease at Hyogo College of Medicine, and we reviewed these data for a retrospective evaluation."* [63]
Demonstration that outcome of interest was not presented at start of study	-	Overall, 16 patients were classified as having a wound class ≥ 3. [63]
Comparability		
Comparability of cohorts on the basis of the design and analysis: controlled for a critical factor and or other medication	⋆	Adjusted by conducting multivariate analysis for overall SSI (use of anti-TNF-α antibody):<br>*"In addition, the following nine factors were included in the multivariate logistic regression analysis: age ≥ 65 years, pre-operative serum Alb < 2.5 g/dL, anti-TNF-α antibody use,*

		ASA score ≥ 3, wound class ≥ 3, urgent/emergent surgery, duration of surgery ≥ 252 min, amount of blood loss during surgery ≥ 330 mL, and perioperative blood transfusion." [63]<br><br>Adjusted by conducting multivariate analysis for incisional SSI (total amount of prednisolone administered ≥ 9000 mg): *"In addition, the following six factors were included in the multivariate logistic regression analysis: age ≥ 65 years, total amount of PSL administered ≥ 9000 mg, ASA score ≥ 3, surgical indication of cancer/dysplasia, amount of blood loss during surgery ≥ 330 mL, and perioperative blood transfusion."* [63]<br><br>Adjusted by conducting multivariate analysis for organ/ space SSI (use of preoperative prednisolone dose ≥ 40 mg/day and anti-TNF-α antibody): *"In addition, the following eight factors were included in the multivariate logistic regression analysis: pre-operative serum Alb < 2.5 g/dL, preoperative PSL dose ≥ 40 mg/day, anti-TNF-α antibody use, ASA score ≥ 3, urgent/emergent surgery, duration of surgery ≥ 252 min, amount of blood loss during surgery ≥ 330 mL, and perioperative blood transfusion. […] In O/S SSI, no significant risk factors were found in the univariate analysis. The following 6 factors were included in the multivariate logistic regression analysis: disease severity ≥ severe, PSL use, AZA/6-MP use, intra-operative bleeding ≥ 232 ml, ASA score ≥ 3 and BS level ≥ 180 mg/dl."* [63]
Comparability of cohorts on the basis of the design and analysis: controlled for additional factor	⋆	Adjusted by conducting multivariate analysis for overall SSI (age ≥ 65 years; pre-operative serum Albumin < 2.5 g/dL; ASA score ≥ 3; wound class ≥ 3; urgent/emergent surgery; duration of surgery ≥ 252 min; amount of blood loss during surgery ≥ 330 mL and perioperative blood transfusion): *"In addition, the following nine factors were included in the multivariate logistic regression analysis: age ≥ 65 years, pre-operative serum Alb < 2.5 g/dL, anti-TNF-α antibody use, ASA score ≥ 3, wound class ≥ 3, urgent/emergent surgery, duration of surgery ≥ 252 min, amount of blood loss during surgery ≥ 330 mL, and perioperative blood transfusion."* [63]<br><br>Adjusted by conducting multivariate analysis for incisional SSI (age ≥ 65 years; ASA score ≥ 3; surgical indication of cancer/ dysplasia; amount of blood loss during surgery ≥ 330 mL and perioperative blood transfusion): *"In addition, the following six factors were included in the multivariate logistic regression analysis: age ≥ 65*

		years, total amount of PSL administered ≥ 9000 mg, ASA score ≥ 3, surgical indication of cancer/dysplasia, amount of blood loss during surgery ≥ 330 mL, and perioperative blood transfusion" [63]<br><br>Adjusted by conducting multivariate analysis for organ/ space SSI (pre-operative serum Albumin < 2.5 g/dL; preoperative prednisolone dose ≥ 40 mg/day; anti-TNF-α antibody use; ASA score ≥ 3; urgent/emergent surgery; duration of surgery ≥ 252 min; amount of blood loss during surgery ≥ 330 mL and perioperative blood transfusion): *"In addition, the following eight factors were included in the multivariate logistic regression analysis: pre-operative serum Alb < 2.5 g/dL, preoperative PSL dose ≥ 40 mg/day, anti-TNF-α antibody use, ASA score ≥ 3, urgent/emergent surgery, duration of surgery ≥ 252 min, amount of blood loss during surgery ≥ 330 mL, and perioperative blood transfusion."* [63]
Outcome		
Assessment of outcome	⋆	Data obtained from a prospectively maintained database of Hyogo College of Medicine and records were reviewed retrospectively. [63]
Was the follow-up long enough for outcomes to occur	⋆	*"The criterion for a diagnosis of SSI was an infection that occurred within 30 days after the operation […]"* [63]
Adequacy of follow up of cohorts	-	No statement.
**Total score**	⋆ ⋆ ⋆ ⋆ ⋆ ⋆ ⋆ (7)	

**Table S2.40.** NOS assessment [22] – Study ID: Ward et al., 2018 [67]		
**Criteria for cohort studies**	**Authors judgement**	**Support for judgement**
Selection		
Representativeness of the exposed cohort	⋆	*"Hospital Episode Statistics (HES) data were used to identify patients undergoing a subtotal colectomy for UC. HES are data maintained by NHS Digital providing details of all NHS funded admissions to hospitals in England. Within the NHS in England, the anti-*

		TNF agents infliximab, adalimumab and golimumab are classified as high cost drugs, the use of which must be coded to obtain funding using the Office of Population Censuses and Surveys (OPCS) Classification of Interventions and Procedures code, version 4. […] All patients with a diagnosis of UC (International Classification of Disease, ICD-10 code K51) undergoing subsequent subtotal colectomy OPCS-4 codes H05.2, H05.3, H05.8, H05.0, H29) between 1 April 2006 and 31 March 2015 made up the study population." [67]
Selection of the non-exposed cohort	⋆	The exposed and non-exposed cohort were obtained from the same database, hospitals and time period: *"[…] leaving a final study population of 6225 patients (see Fig. 1). 753 patients (12.1%) received anti- TNF therapy in the 12-week period prior to colectomy. A smaller group of 418 patients (6.7%) received anti- TNF therapy in the 4-week period prior to colectomy."* [67]
Ascertainment of exposure	⋆	Data obtained by codes using the Office of Population Censuses and Surveys (OPCS) Classification of Interventions and Procedure code, version 4: *"Within the NHS in England, the anti-TNF agents infliximab, adalimumab and golimumab are classified as high cost drugs, the use of which must be coded to obtain funding using the Office of Population Censuses and Surveys (OPCS) Classification of Interventions and Procedures code, version 4 […] Patients were defined as receiving anti-TNF therapy by the presence of the OPCS-4 code X92.1 occurring."* [67]
Demonstration that outcome of interest was not presented at start of study	-	No statement.
Comparability		
Comparability of cohorts on the basis of the design and analysis: controlled for a critical factor and or other medication	-	Not adjusted for critical factor and or other medication.
Comparability of cohorts on the basis of the design and analysis: controlled for additional factor	⋆	Adjusted for demographic variables (age group; gender; ethnicity; deprivation; Charlson score; smoking status; surgical approach; colectomy as unplanned date and colectomy during spells of UC diagnosis) by conducting a multivariate analysis: *"A multivariate logistic regression model was performed using any postoperative complication as the outcome variable with the use of anti-TNF therapy within 12 or 4 weeks preoperatively and all demographic variables listed in Table 1 as the covariates."* [67]
Outcome		
Assessment of outcome	⋆	Data obtained by ICD-10 codes: *"A number of different postoperative complications were extracted (cardiovascular, pulmonary, gastrointestinal, neurological, renal/endocrine,*

		infectious and wound problems) by way of ICD-10 diagnoses occurring within 30 days of the date of subtotal colectomy." [67]
Was the follow-up long enough for outcomes to occur	⋆	*"A number of different postoperative complications were extracted (cardiovascular, pulmonary, gastrointestinal, neurological, renal/endocrine, infectious and wound problems) by way of ICD-10 diagnoses occurring within 30 days of the date of subtotal colectomy."* [67]
Adequacy of follow up of cohorts	-	No statement.
**Total score**	⋆ ⋆ ⋆ ⋆ ⋆ ⋆ (6)	

Table S2.41. NOS assessment [22] – Study ID: Zittan et al., 2016 [74]

Criteria for cohort studies	Authors judgement	Support for judgement
Selection		
Representativeness of the exposed cohort	⋆	*"Data on all patients with UC who underwent IPAA from January 2002 to June 2013 were retrieved for review from the inflammatory bowel disease (IBD) Surgical Database at Mount Sinai Hospital in Toronto, Canada. The clinical characteristics, dates, and descriptions of the operative procedures were documented, and the data extracted were independently assessed by 2 physicians (E.Z., R.M.)."* [74]
Selection of the non-exposed cohort	⋆	The exposed and non-exposed cohort were obtained from the same database, hospitals and time period: *"Individuals with documented preoperative exposure to anti-TNF (n = 196) compared with the control group (individuals who were not exposed to biological therapy, n = 562). Anti-TNF exposure was verified by reviewing both inpatient and outpatient charts of each individual."* [74]
Ascertainment of exposure	⋆	Medical charts were reviewed: *"The clinical characteristics, dates, and descriptions of the operative procedures were documented, and the data extracted were independently assessed by 2 physicians (E.Z., R.M.)."* [74]
Demonstration that outcome of interest was not presented at start of study	-	No statement.

Comparability	⋆	*We decided to give one star for comparability because some of the important factors were adjusted, while others were not.*
Comparability of cohorts on the basis of the design and analysis: controlled for a critical factor and or other medication		Adjusted for steroid use by conducting multivariate analysis: *"In our multivariate analysis, the use of steroids was independently associated with the occurrence of short-term postoperative infectious complications."* [74]
Comparability of cohorts on the basis of the design and analysis: controlled for additional factor		Adjusted for the stage of surgery by conducting multivariate analysis: *"Moreover, we found no significant association between anti-TNF use and combined adverse outcome (DVT, ileus, pelvic abscess, and anastomotic leaks) into one in the multivariate analysis adjusting the stage of IPAA surgery, P = 0.55 (OR 1.41, 95% CI 0.63–3.17)."* [74]
Outcome		
Assessment of outcome	⋆	Medical charts were reviewed. [74]
Was the follow-up long enough for outcomes to occur	⋆	*"Patients without adequate clinical documentation of the 30-day postoperative period were excluded."* [74]
Adequacy of follow up of cohorts	⋆	*"Patients without adequate clinical documentation of the 30-day postoperative period were excluded."* [74]
**Total score**	⋆ ⋆ ⋆ ⋆ ⋆ ⋆ ⋆ (7)	

Table S2.42. NOS assessment [22] – Study ID: El-Hussuna et al., 2018 [32]

Criteria for cohort studies	Authors judgement	Support for judgement
Selection		
Representativeness of the exposed cohort	⋆	*"The null hypothesis was that pre-operative administration of anti-TNF-α agents within 12 weeks before surgery, have no significant effect on surgical stress response. To investigate this; a prospective, non-interventional multi-center pilot study was designed. […] Inclusion criteria: adult patients with Crohn's disease (CD) or ulcerative colitis (UC) who were scheduled to elective intestinal resection or terminal stoma closure in three Danish university hospitals during the study period (March 2014–May 2016). Open as well as laparoscopic approaches were included."* [32]

Selection of the non-exposed cohort	⋆	The exposed and non-exposed cohort were obtained from the same hospitals and time period: *"This explorative study succeeded, thus, in recruiting 46 patients, of which 18 had one type or another of anti-TNF-α agent treatment within 3 months prior to surgery."* [32]
Ascertainment of exposure	⋆	Data obtained from a prospectively maintained database and medical records were examined: *"Age, gender, duration of disease, number of previous laparotomies, intraabdominal infection as preoperative abscess or fistula, and medication for Crohn's disease were recorded preoperatively. […] Patients were identified using outpatient's clinic records, operation lists and reports from IBD conferences. All patients who fulfilled the inclusion criteria accepted to participate in the project."* [32]
Demonstration that outcome of interest was not presented at start of study	-	No statement.
Comparability	⋆	*We decided to give one star for comparability since some adjustment was taken*
Comparability of cohorts on the basis of the design and analysis: controlled for a critical factor and or other medication		*"We did not find an association between anti-TNF-α treatment and post-operative outcomes. This did not appear to be explained by possible clinically and intra-operative factors that might influence the stress response to surgery or drug concentration and presence of anti-drug antibodies, as we adjusted for these […]"* [32]
Comparability of cohorts on the basis of the design and analysis: controlled for additional factor		*"We did not find an association between anti-TNF-α treatment and post-operative outcomes. This did not appear to be explained by possible clinically and intra-operative factors that might influence the stress response to surgery or drug concentration and presence of anti-drug antibodies, as we adjusted for these […]"* [32]
Outcome		
Assessment of outcome	⋆	Data obtained from a prospectively maintained database and medical records were examined. [32]
Was the follow-up long enough for outcomes to occur	⋆	*"[…] in addition to 30-days, postoperative complication and length of hospital stay (LOS)."* [32]
Adequacy of follow up of cohorts	-	No statement.
**Total score**	⋆ ⋆ ⋆ ⋆ ⋆ ⋆ (6)	

**Table S2.43.** NOS assessment [22] – Study ID: Krane et al., 2013 [42]		
**Criteria for cohort studies**	**Authors judgement**	**Support for judgement**
Selection		
Representativeness of the exposed cohort	⋆	*"This study is a retrospective analysis of a prospectively collected, institutional review board-approved IBD database. Consecutive IBD patients referred for surgical management at the University of Chicago Medical Center from January 2004 through June 2011 and treated laparoscopically were identified."* [42]
Selection of the non-exposed cohort	⋆	The exposed and non-exposed cohort were obtained from the same database, hospitals and time period: *"As a result, 518 consecutive patients treated by minimally invasive resection were included in the study. One hundred forty-two patients underwent preoperative therapy with IFX (IFX group), whereas 376 did not receive IFX (NIFX group)."* [42]
Ascertainment of exposure	⋆	Data obtained from a prospectively collected database. [42]
Demonstration that outcome of interest was not presented at start of study	-	Overall, 7% of exposed cohort (10 out of 142) and 3,7% of non-exposed cohort (14 out of 376) presented with a Clostridium difficile infection preoperatively. [42]
Comparability		
Comparability of cohorts on the basis of the design and analysis: controlled for a critical factor and or other medication	⋆	Adjusted for use of steroids and other immunosuppressants by conducting multivariate analysis. [42]
Comparability of cohorts on the basis of the design and analysis: controlled for additional factor	⋆	Adjusted for type of IBD; activity of disease and comorbidities by conducting multivariate analysis. [42]
Outcome		
Assessment of outcome	⋆	Data obtained from a prospectively collected database. [42]

Was the follow-up long enough for outcomes to occur	⋆	*"A prospective protocol was designed to evaluate the following parameters: patient's characteristics, indications for surgery, operative variables, short-term (within 30 days from surgery) morbidity according to Dindo classification, 12 long-term morbidity, and reoperation rate."* [42]
Adequacy of follow up of cohorts	⋆	*"Only patients with a 6-month minimum follow-up were considered."* [42]
**Total score**	⋆ ⋆ ⋆ ⋆ ⋆ ⋆ ⋆ ⋆ (8)	

**Table S2.44.** NOS assessment [22] – Study ID: Kunitake et al., 2008 [43]		
**Criteria for cohort studies**	**Authors judgement**	**Support for judgement**
Selection		
Representativeness of the exposed cohort	⋆	*"Retrospective data were gathered on 413 consecutive patients who underwent abdominal surgery at MGH for complications of CD, UC, and indeterminate colitis (IC) between January 15, 1993 and June 27, 2007. These patients were identified from 1.8 million patients in the MGH medical records database, using the research patient database query tool (research patient data repository) to identify patients who underwent abdominal surgery for the diagnosis of UC (ICD-9:556), toxic gastroenteritis and colitis (ICD-9:558.2), CD (ICD-9:555), regional enteritis of the large intestine (ICD-9:555.1), regional enteritis of the small with large intestine (ICD-9:555.2), regional enteritis of the small intestine alone (ICD-9:555), and regional enteritis of an unspecified site (ICD-9:555.9; Fig. 1)."* [43]
Selection of the non-exposed cohort	⋆	The exposed and non-exposed cohort were obtained from the same database, hospitals and time period: *"This search identified 101 patients who received IFX≤ 12 weeks before surgery. These 101 patients were then compared to the remaining 312 patients with respect to demographics, comorbidities, preoperative nutritional status, surgical indications, intraoperative findings, and rates of postoperative complications (defined as an*

		occurrence within the first 30 days after the surgical procedure or during the index admission).” [43]
Ascertainment of exposure	⋆	Medical records were reviewed: *“This initial search identified 455 patients, whose electronic medical records were individually reviewed to confirm that IBD had been recorded as the indication for surgery. […] This search identified 101 patients who received IFX≤ 12 weeks before surgery. These 101 patients were then compared to the remaining 312 patients with respect to demographics, comorbidities, preoperative nutritional status, surgical indications, intraoperative findings, and rates of postoperative complications (defined as an occurrence within the first 30 days after the surgical procedure or during the index admission).”* [43]
Demonstration that outcome of interest was not presented at start of study	-	Overall, 4% of exposed cohort (4 out of 101) and 10,9% of non-exposed cohort (34 out of 312) presented with an intra-abdominal abscess preoperatively. [43]
Comparability		
Comparability of cohorts on the basis of the design and analysis: controlled for a critical factor and or other medication	-	Unclear whether univariate or multivariate analysis was performed: *“Logistic regression analysis seeking to identify other factors that might have impacted the rates of postoperative infections was then performed. The model included the presence of a preexisting intra-abdominal abscess and steroid exposure and the diagnosis of CD or UC and IFX exposure. These variables were chosen either because the variable was statistically different between the IFX and non-IFX groups on our prior univariate comparison of cohorts or based on prior literature. None of these variables were found to be a reliable predictor of postoperative infections (steroids [OR=1.2, p=0.74], IFX [OR 2.5, p=0.14], preoperative diagnosis of CD [OR 0.7, p=0.63] or UC [OR 0.6, p=0.48], and preoperative infection [OR=1.2, p=0.76]).”* [43]
Comparability of cohorts on the basis of the design and analysis: controlled for additional factor	-	Unclear whether univariate or multivariate analysis was performed: *“Logistic regression analysis seeking to identify other factors that might have impacted the rates of postoperative infections was then performed. The model included the presence of a preexisting intra-abdominal abscess and steroid exposure and the diagnosis of CD or UC and IFX exposure. These variables were chosen either because the variable was statistically different between the IFX and non-IFX groups on our prior univariate comparison of*

		cohorts or based on prior literature. None of these variables were found to be a reliable predictor of postoperative infections (steroids [OR=1.2, p=0.74], IFX [OR 2.5, p=0.14], preoperative diagnosis of CD [OR 0.7, p=0.63] or UC [OR 0.6, p=0.48], and preoperative infection [OR=1.2, p=0.76]).” [43]
Outcome		
Assessment of outcome	⋆	Medical records were reviewed: *“This search identified 101 patients who received IFX≤ 12 weeks before surgery. These 101 patients were then compared to the remaining 312 patients with respect to demographics, comorbidities, preoperative nutritional status, surgical indications, intraoperative findings, and rates of postoperative complications (defined as an occurrence within the first 30 days after the surgical procedure or during the index admission).”* [43]
Was the follow-up long enough for outcomes to occur	⋆	*“[…] postoperative complications (defined as an occurrence within the first 30 days after the surgical procedure or during the index admission).”* [43]
Adequacy of follow up of cohorts	-	No statement.
**Total score**	⋆ ⋆ ⋆ ⋆ ⋆ (5)	

**Table S2.45.** NOS assessment [22] – Study ID: Lau et al., 2015 [21]		
**Criteria for cohort studies**	**Authors judgement**	**Support for judgement**
Selection		
Representativeness of the exposed cohort	⋆	*“Consecutive UC and CD adult patients undergoing major abdominal surgery by a single surgeon in a tertiary referral center over a 13-year period ending October 2012 were initially identified. […] A prospectively maintained IBD registry of patient’s clinical profiles including demographics and disease characteristics was retrospectively reviewed. Demographic information included patient sex, age at time of surgery, preoperative morbidity, and smoking history. Disease characteristics included type of IBD (UC or CD), type of preoperative medication use, […]”* [21]

Selection of the non-exposed cohort	⋆	The exposed and non-exposed cohort were obtained from the same database, hospitals and time period: *"From December 1999 to October 2012, 217 (21%) patients satisfied study entry criteria and comprised the study cohort (Table 1). The mean age of the study cohort was 36.9 years (SD, 15.5), and 57% of the study cohort had CD. Anti-TNFα agents were used before surgery in 65% of the study cohort, most commonly infliximab. […] Sixty-seven study cohort patients (31%) had detectable serum anti-TNFα drug levels (Table 1)."* [21]
Ascertainment of exposure	⋆	Data obtained from a prospectively collected database and medical records were reviewed: *"A prospectively maintained IBD registry of patient's clinical profiles including demographics and disease characteristics was retrospectively reviewed. Demographic information included patient sex, age at time of surgery, preoperative morbidity, and smoking history. Disease characteristics included type of IBD (UC or CD), type of preoperative medication use, […]"* [21]
Demonstration that outcome of interest was not presented at start of study	-	Overall, 10 % of exposed cohort (7 out of 67 patients) and 5% of non-exposed cohort (8 out of 150 patients) patients presented with intra-abdominal abscess at surgery. [21]
Comparability		
Comparability of cohorts on the basis of the design and analysis: controlled for a critical factor and or other medication	⋆	Adjusted for use of steroids, 6-MP, AZA and albumin by conducting a multivariate analysis: *"Model Analysis of Postoperative Outcomes in CD Patients Using Multivariate Logistic Regression and Serum Anti-TNFα Drug Level Cutoff Value of 3 μg/mL […]"* [21]
Comparability of cohorts on the basis of the design and analysis: controlled for additional factor	-	Not adjusted for additional factor.
Outcome		
Assessment of outcome	⋆	Data obtained from a prospectively collected database: *"Postoperative morbidity and mortality were prospectively recorded during the 30-day period beginning from the date of surgery using inpatient medical records and office chart notes. […] Postsurgical length of hospitalization and 30-day hospital readmission rated were also noted."* [21]
Was the follow-up long enough for outcomes to occur	⋆	*"Postoperative morbidity and mortality were prospectively recorded during the 30-day period beginning from the date of surgery using inpatient medical records and office chart notes. […] Postsurgical length of hospitalization and 30-day hospital readmission rated were also noted."* [21]
Adequacy of follow up of cohorts	-	No statement.

**Total score**	⋆ ⋆ ⋆ ⋆ ⋆ ⋆ (6)	

**Table S2.46.** NOS assessment [22] – Study ID: Monsinjon et al., 2017 [47]		
**Criteria for cohort studies**	**Authors judgement**	**Support for judgement**
Selection		
Representativeness of the exposed cohort	⋆	*"All the patients who underwent subtotal colectomy for SAC after failure of medical treatment were identified from our prospective single-center institutional review board approved database. Two groups of patients were constituted according to the occurrence of postoperative morbidity during the hospital stay or within 30 days after surgery: […] From January 2006 to October 2015, 65 patients undergoing subtotal colectomy for SAC were included. Nineteen patients presented with postoperative morbidity (29%, group A) and 46 patients had an uneventful course (71%, group B)."* [47]
Selection of the non-exposed cohort	⋆	The exposed and non-exposed cohort were obtained from the same database, hospitals and time period: *"At the time of surgery, 37 and 39% of patients from groups A and B were under anti-TNF therapy, respectively (p = 1.00)."* [47]
Ascertainment of exposure	⋆	Data obtained from a prospectively maintained database:*"All the patients who underwent subtotal colectomy for SAC after failure of medical treatment were identified from our prospective single-center institutional review board approved database. Two groups of patients were constituted according to the occurrence of postoperative morbidity during the hospital stay or within 30 days after surgery: […]"* [47]
Demonstration that outcome of interest was not presented at start of study	-	No statement.
Comparability		
Comparability of cohorts on the basis of the design and analysis: controlled for a critical factor and or other medication	-	Not adjusted for critical factor and or other medication.

Comparability of cohorts on the basis of the design and analysis: controlled for additional factor	-	Not adjusted for additional factor.
Outcome		
Assessment of outcome	⋆	Data obtained from a prospectively maintained database. [47]
Was the follow-up long enough for outcomes to occur	⋆	*"Two groups of patients were constituted according to the occurrence of postoperative morbidity during the hospital stay or within 30 days after surgery: […]"* [47]
Adequacy of follow up of cohorts	-	No statement.
**Total score**	⋆ ⋆ ⋆ ⋆ ⋆ (5)	

Table S2.47. NOS assessment [22] – Study ID: Regadas et al., 2011 [54]

Criteria for cohort studies	Authors judgement	Support for judgement
Selection		
Representativeness of the exposed cohort	⋆	*"After Institutional Review Board approval, a retrospective analysis was undertaken from a prospectively collected database of patients who underwent ileostomy reversal following ileostomy construction between January 2001 and April 2008."* [54]
Selection of the non-exposed cohort	⋆	The exposed and non-exposed cohort were obtained from the same database, hospitals and time period: *"Twenty eight of 249 patients included in the study, 28 formed the infliximab group, 72 the steroid group, 35 the steroids + immunosuppressive group and 114 the control group."* [54]
Ascertainment of exposure	⋆	Data obtained from a prospectively collected database. [54]
Demonstration that outcome of interest was not presented at start of study	-	No statement.

Comparability		
Comparability of cohorts on the basis of the design and analysis: controlled for a critical factor and or other medication	-	Not adjusted for critical factor and or other medication.
Comparability of cohorts on the basis of the design and analysis: controlled for additional factor	-	Not adjusted for additional factor.
Outcome		
Assessment of outcome	⋆	Data obtained from a prospectively collected database. [54]
Was the follow-up long enough for outcomes to occur	⋆	*"[…] postoperative complications and mortality within 30 days after ileostomy closure were recorded [9]."* [54]
Adequacy of follow up of cohorts	-	No statement.
**Total score**	⋆ ⋆ ⋆ ⋆ ⋆ (5)	

Table S2.48. NOS assessment [22] – Study ID: Rizzo et al., 2011 [55]

Criteria for cohort studies	Authors judgement	Support for judgement
Selection		
Representativeness of the exposed cohort	⋆	*"The medical records of patients attending the IBD Unit at Complesso Integrato Columbus Hospital of the Catholic University of Rome and who underwent CD- or UC-related abdominal surgery between January 2004 and May 2010 were retrospectively analyzed."* [55]
Selection of the non-exposed cohort	⋆	The exposed and non-exposed cohort were obtained from the same database, hospitals and time period: *"From January 2004 to May 2010, 114 patients (71 male and 43 female) with a median age of 39 years (range 16–74) underwent abdominal surgery for IBD in our division. Seventy-six patients (67%) were affected by CD and 38 (33%) by UC (Table 1). Fifty-four patients (47%; 37 CD and 17 UC) were treated with anti-TNF-alpha within 12 weeks of surgery and categorized in the anti-TNF group."* [55]
Ascertainment of exposure	⋆	Medical records were analysed. [55]

Demonstration that outcome of interest was not presented at start of study	-	No statement.
Comparability		
Comparability of cohorts on the basis of the design and analysis: controlled for a critical factor and or other medication	⋆	Adjusted for steroid use and anti-TNF and high-dose steroid use by conducting a multivariate analysis: *"After multivariate analysis (Table 5), therapy with high-dose corticosteroid was determined to be an independent factor influencing the incidence of postoperative complications (OR: 7.3048; CI: 1.4157–37.6921; P value=0.0175). […] After multivariate analysis (Table 7), only therapy with high dose of corticosteroid was shown to be an independent factor for increasing the risk of postoperative infectious complications (OR: 5.5875; CI: 1.0264–30.4171; P value=0.0466)."* [55]
Comparability of cohorts on the basis of the design and analysis: controlled for additional factor	⋆	Adjusted for age; gender and previous surgery for IBD by conducting a multivariate analysis: *"Parameters with a P value <0.125 by univariate analysis were entered into a multivariate logistic regression model to identify independent predictors for the occurrence of postoperative complications"* [55]
Outcome		
Assessment of outcome	⋆	Medical records were analysed. [55]
Was the follow-up long enough for outcomes to occur	⋆	*"Postoperative complications within 30 days after surgery were recorded and grouped into five general categories as previously […]"* [55]
Adequacy of follow up of cohorts	-	No statement.
**Total score**	⋆ ⋆ ⋆ ⋆ ⋆ ⋆ ⋆ (7)	

**Table S2.49.** NOS assessment [22] – Study ID: Shwaartz et al., 2016 [58]		
**Criteria for cohort studies**	**Authors judgement**	**Support for judgement**
Selection		
Representativeness of the exposed cohort	⋆	*"This study is a retrospective review of 282 patients with UC or CD from 2013 to 2015 undergoing intestinal surgery with primary anastomosis at a tertiary referral center for IBD. The study was approved by the local IRB at the Mount Sinai Medical Center in New York,*

		NY. Patients who were less than 18 years of age, underwent emergency operation, or did not have anastomosis were excluded […]" [58]
Selection of the non-exposed cohort	⋆	The exposed and non-exposed cohort were obtained from the same database, hospitals and time period: *"Two hundred eighty-two IBD patients who had intestinal surgery were identified. Seventy-three patients (25.9 %) took anti-TNF medications within 2 months of their operation, and 209 patients (74.1 %) were not exposed to anti-TNF medications within 2 months of their surgery (Table 1) […]"* [58]
Ascertainment of exposure	⋆	Electronical medical records were reviewed: *"Electronical medical records and operative/anesthesia reports were reviewed. […] The patient's electronic medical record was assessed to determine usage of steroids (i.e., prednisone, hydrocortisone, budesonide), immunomodulators (i.e., methotrexate, mesalamine, azathioprine), and anti-TNF medications (infliximab, adalimumab, certolizumab pegol)."* [58]
Demonstration that outcome of interest was not presented at start of study	-	No statement.
Comparability	⋆	*We decided to give one star for comparability since some important factors were adjusted, while others were not.*
Comparability of cohorts on the basis of the design and analysis: controlled for a critical factor and or other medication		Adjusted by conducting a multivariate analysis for anastomotic leak (use of anti-TNF agents; use of corticosteroids; use of immunomodulatory drugs); for intra-abdominal abscess (use of anti-TNF agents); for wound infection (use of anti-TNF agents; use of 5-ASA); for extra-abdominal infection (use of anti-TNF agents); for readmission (use of anti-TNF agents): *"Multivariate logistic regression demonstrated that exposure to anti-TNF medications was not an independent risk factor for developing postoperative anastomotic leak (p = 0.59, OR 1.64, 95%CI 0.27–10.21), intra- abdominal abscess (p = 0.12, OR 3.09, 95 % CI 0.73–13.06), wound infection (p = 0.45, OR 1.56, 95 % CI 0.49–4.92), extra-abdominal infection (p = 0.24, OR 2.53, 95 % CI 0.54–11.96), and readmission (p = 0.48, OR 1.46, 95 % CI 0.51–4.15) (Table 3). Preoperative steroid use (p = 0.049) and handsewn anastomosis (p= 0.01) were independent predictors of anastomotic leak in multivariate logistic regression."* [58]
Comparability of cohorts on the basis of the design and analysis: controlled for additional factor		Adjusted by conducting a multivariate analysis for anastomotic leak (handsewn anastomosis; albumin); for intra-abdominal abscess (coronary artery disease); for wound infection (handsewn anastomosis); for extra-abdominal infection (hypertension); for readmission (smoking; BMI): *"Multivariate logistic regression demonstrated that exposure to anti-TNF medications was not an independent risk factor for developing postoperative anastomotic leak (p = 0.59, OR 1.64, 95%CI 0.27–10.21), intra-*

		abdominal abscess (p = 0.12, OR 3.09, 95 % CI 0.73–13.06), wound infection (p = 0.45, OR 1.56, 95 % CI 0.49–4.92), extra-abdominal infection (p = 0.24, OR 2.53, 95 % CI 0.54–11.96), and readmission (p = 0.48, OR 1.46, 95 % CI 0.51–4.15) (Table 3). Preoperative steroid use (p = 0.049) and handsewn anastomosis (p= 0.01) were independent predictors of anastomotic leak in multivariate logistic regression." [58]
Outcome		
Assessment of outcome	⋆	Medical records were reviewed. [58]
Was the follow-up long enough for outcomes to occur	⋆	*"We examined the 30-day postoperative outcomes of IBD patients who received anti-TNF agents within 2 months of surgery […]"*[58]
Adequacy of follow up of cohorts	-	No statement. [58]
**Total score**	⋆ ⋆ ⋆ ⋆ ⋆ ⋆ (6)	

Table S2.50. NOS assessment [22] – Study ID: Waterman et al., 2012 [68]

Criteria for cohort studies	Authors judgement	Support for judgement
Selection		
Representativeness of the exposed cohort	⋆	*"The IBD Surgical Database at Mount Sinai Hospital in Toronto, Canada contains prospectively collected demographic and phenotypic data for all patients with IBD who underwent surgery since 1989. The dates and detailed description of the operative procedures are documented. The data on all IBD affected individuals who underwent abdominal surgery during the biologic era from January 2000 to June 2010 were reviewed. Those individuals with documented preoperative exposure to infliximab and/or adalimumab within 180 days from the date of surgery were selected. Anti-tumour necrosis factor (anti-TNF) exposure was verified by reviewing both the inpatient and outpatient charts of each individual."* [68]
Selection of the non-exposed cohort	⋆	*"Matched controls selection. Subjects were chosen from the same database as described above. Non-exposure to biologics within 180 days before the date of operation was verified."* [68]
Ascertainment of exposure	⋆	*"The IBD Surgical Database at Mount Sinai Hospital in Toronto, Canada contains prospectively collected demographic and phenotypic data for all patients with IBD who underwent surgery since 1989. The dates and detailed description of the operative procedures are documented. The data on all IBD affected individuals who underwent*

		abdominal surgery during the biologic era from January 2000 to June 2010 were reviewed. Those individuals with documented preoperative exposure to infliximab and/or adalimumab within 180 days from the date of surgery were selected. Anti-tumour necrosis factor (anti-TNF) exposure was verified by reviewing both the inpatient and outpatient charts of each individual." [68]
Demonstration that outcome of interest was not presented at start of study	-	No statement.
Comparability		
Comparability of cohorts on the basis of the design and analysis: controlled for a critical factor and or other medication	⋆	Matched for exposure of preoperative prednisone ≥ 20 mg/day or equivalent corticosteroid use within 7 days: *"Subjects were chosen from the same database as described above. Non-exposure to biologics within 180 days before the date of operation was verified. Only patients who had complete documentation as described above were reviewed. Care was taken to ensure that each control procedure was matched to only one case procedure. For each procedure on biologics, we carefully matched 1e2 control procedures using the following four criteria: 1. Main operative procedure (eg, small bowel resection, subtotal colectomy (STC), etc) 2. IBD subtype (CD vs UC/IBD unclassified) 3. Exposure to preoperative prednisone ≥20 mg/day or equivalent CS within 7 days 4. Patient age at surgery (<50 or ≥50 years)."* [68]
Comparability of cohorts on the basis of the design and analysis: controlled for additional factor	⋆	Matched based on operative procedure, IBD subtype and patient age: *"Subjects were chosen from the same database as described above. Non-exposure to biologics within 180 days before the date of operation was verified. Only patients who had complete documentation as described above were reviewed. Care was taken to ensure that each control procedure was matched to only one case procedure. For each procedure on biologics, we carefully matched 1e2 control procedures using the following four criteria: 1. Main operative procedure (eg, small bowel resection, subtotal colectomy (STC), etc) 2. IBD subtype (CD vs UC/IBD unclassified) 3. Exposure to preoperative prednisone ≥20 mg/day or equivalent CS within 7 days 4. Patient age at surgery (<50 or ≥50 years)."* [68]<br><br>Adjusted for acute disease activity and laparoscopy by conducting multivariate analysis. [68]
Exposure		
Assessment of outcome	⋆	Data obtained from a prospectively maintained database and medical records were reviewed. [68]

Was the follow-up long enough for outcomes to occur	⋆	*"In this study, subjects with IBD undergoing abdominal surgery with recent exposure to infliximab or adalimumab were evaluated to assess for 30 day surgical outcomes in a large surgical tertiary care referral centre."* [68]
Adequacy of follow up of cohorts	⋆	*"Patients without adequate clinical records documenting the 30 day postoperative clinical outcomes were excluded."* [68]
**Total score**	⋆ ⋆ ⋆ ⋆ ⋆ ⋆ ⋆ ⋆ (8)	

Table S2.51. NOS assessment [22] – Study ID: Yamada et al., 2017 [70]

Criteria for cohort studies	Authors judgement	Support for judgement
Selection		
Representativeness of the exposed cohort	⋆	*"Medical records of patients registered in a prospectively collected IBD database were retrospectively reviewed for those who underwent surgery between June 2014 and April 2016. Th e study was approved by the institutional review board (IRB 16-0061). The study was undertaken based on a priori defi ned protocol. Study patients included adults with UC or CD who received vedolizumab, anti-TNF-α agents or no biological therapy within 4 weeks of an IBD surgery."* [70]
Selection of the non-exposed cohort	⋆	The exposed and non-exposed cohort were obtained from the same database, hospitals and time period: *"Study patients included adults with UC or CD who received vedolizumab, anti-TNF-α agents or no biological therapy within 4 weeks of an IBD surgery."* [70]
Ascertainment of exposure	⋆	Data was obtained from a prospectively collected IBD database and medical records were reviewed retrospectively. [70]
Demonstration that outcome of interest was not presented at start of study	-	No statement.
Comparability		

Comparability of cohorts on the basis of the design and analysis: controlled for a critical factor and or other medication	⋆	Adjusted for use of systemic steroids by conducting a multivariate analysis: *"Multivariate analysis revealed that age >65 years (OR 3.56, 95%CI 1.30–9.76, P =0.01) and low serum albumin level (OR 2.26, 95% CI 1.28–4.00, P =0.005) remained as risk factors for postoperative complications."* [70]
Comparability of cohorts on the basis of the design and analysis: controlled for additional factor	⋆	Adjusted for age (>65); albumin (>3.6 g/dl) and hemoglobin (<10.5 g/dL) by conducting a multivariate analysis: *"Multivariate analysis revealed that age >65 years (OR 3.56, 95%CI 1.30–9.76, P =0.01) and low serum albumin level (OR 2.26, 95% CI 1.28–4.00, P =0.005) remained as risk factors for postoperative complications."* [70]
Outcome		
Assessment of outcome	⋆	Data was obtained from a prospectively collected IBD database and medical records were reviewed retrospectively. [70]
Was the follow-up long enough for outcomes to occur	⋆	*"The primary endpoint was postoperative complications, which were defined as […] occurring within 30 days of the index operation."* [70]
Adequacy of follow up of cohorts	⋆	*"Patients were excluded if they did not have 30 days of follow-up aft er their operation, their operation was performed at an outside hospital, or data was missing."* [70]
**Total score**	⋆ ⋆ ⋆ ⋆ ⋆ ⋆ ⋆ ⋆ (8)	

Abbreviations:

ADA = Adalimumab
ALB = Albumin [63]
ASA Score = American Society of Anesthesiologists Score
AZA = Azathioprine
BIO = Biological Drug
BMI = Body-Mass-Index
CC = Colon Cancer [24]
CCT = Corticosteroids [30]
IRB = Institution Review Board
IV= Intravenous
kg = Kilogram
l = Liter
mg = Milligram
MGH = Massachusetts General Hospital [43]
min. = Minute
ml = Milliliter

CD = Crohn's Disease
CI = Confidence Interval
CNI = Calcineurin Inhibitor
CP = Completion Proctectomy [35]
CyA = Cyclosporine A [63]
dL = Deciliter
e.g. = Exempli Gratia
EI = End Ileostomy [35]
ePOC = Early Postoperative Complication [36]
g = Gram
GETAID = Groupe d`étude Thérapeutique des Affections Inflammatoires du Tube Digestif [4]
GOL = Golimumab [63]
HSJ = São João Hospital Center [30]
IASC = Intraabdominal Septic Complication
IBD = Inflammatory Bowel Disease
IBDU = Inflammatory Bowel Disease of Unknown Origin
IC = Indeterminate Colitis [35]
ICD = International Classification of Diseases
ICR = Ileocolic Resection [9]
ID: Identity Document
i.e. = Id Est
IFX = Infliximab
INC SSI = Incisional Surgical-Site Infection [63]
IPAA = Ileal Pouch-Anal Anastomosis
MO = Missouri [61]
m^2 = Square Meter
ND = No Drug Group [29]
NHS = National Health Service **[67]**
NOS = Newcastle-Ottawa Scale
OD = Immunosuppressive Agent Group [29]
OR = Odds Ratio
O/S SSI = Organ/ Space Surgical-Site Infection [63]
PIIC = Postoperative Intra-Abdominal Infectious Complications
PSL = Prednisolone
PSM = Propensity Score method [40]
SAC = Severe Acute Colitis [47]
SD = Standard Deviation
SSI = Surgical-Site Infection
STC = Subtotal Colectomy [35]
TNF = Tumor Necrosis Factor
TPC = Total Proctocolectomy
UC = Ulcerative Colitis
UGI = Upper Gastrointestinal Tract [48]
UMB = University of Maryland, Baltimore IBD Program [59]
UR = Unplanned Hospital Readmission **[69]**
5-ASA = 5-Aminosalicylic Acid
6-MP = 6-Mercaptopurine
µg = Microgram

Table S3.1.-3.2.: Other 30-day postoperative complications

Table S3.1 Other 30-day general and surgical-site postoperative complications.

Author and year	General postoperative complications †				Surgical site-complications‡											
	ONIPC, n		OC-Dm, n		S-SSI, n		D-SSI, n		FIS, n		ILE, n		SBO, n		HEM, n	
	I §	C §	I §	C §	I §	C §	I §	C §	I §	C §	I §	C §	I §	C §	I §	C §
**Appau et al., 2008 [25]**	n.a.	n.a.	n.a.	n.a.	n.a.	n.a.	n.a.	n.a.	n.a.	n.a.	n.a.	n.a.	n.a.	n.a.	n.a.	n.a.
**Bafford et al., 2013 [26]**	n.a.	n.a.	n.a.	n.a.	n.a.	n.a.	n.a.	n.a.	n.a.	n.a.	n.a.	n.a.	n.a.	n.a.	n.a.	n.a.
**Bregnbak et al., 2012 [27]**	6	10	n.a.	n.a.	n.a.	n.a.	n.a.	n.a.	n.a.	n.a.	6	7	n.a.	n.a.	n.a.	n.a.
**Brouquet et al., 2018 [28]**	n.a.	n.a.	n.a.	n.a.	n.a.	n.a.	n.a.	n.a.	n.a.	n.a.	n.a.	n.a.	n.a.	n.a.	n.a.	n.a.
**Canedo et al., 2010 [29]**	n.a.	n.a.	n.a.	n.a.	n.a.	n.a.	n.a.	n.a.	n.a.	n.a.	n.a.	n.a.	n.a.	n.a.	n.a.	n.a.
**El-Hussuna et al., 2012 [31]**	n.a.	n.a.	n.a.	n.a.	n.a.	n.a.	n.a.	n.a.	n.a.	n.a.	n.a.	n.a.	n.a.	n.a.	n.a.	n.a.
**El-Hussuna et al., 2018 [32]**	n.a.	n.a.	n.a.	n.a.	1	2	n.a.	n.a.	n.a.	n.a.	n.a.	n.a.	n.a.	n.a.	n.a.	n.a.

**Ferrante et al., 2009 [33]**	n.a.	n.a.	n.a.	n.a.	n.a.	n.a.	n.a.	n.a.	n.a.	n.a.	n.a.	n.a.	n.a.	n.a.	n.a.	n.a.
**Gainsbury et al., 2011 [34]**	12	16	n.a.	n.a.	n.a.	n.a.	n.a.	n.a.	n.a.	n.a.	n.a.	n.a.	n.a.	n.a.	n.a.	n.a.
**Gu et al., 2013 *(1)* * [35]**	n.a.	n.a.	n.a.	n.a.	n.a.	n.a.	n.a.	n.a.	n.a.	n.a.	n.a.	n.a.	1	6	0	5
**Gu et al., 2013 *(2)* * [35]**	n.a.	n.a.	n.a.	n.a.	n.a.	n.a.	n.a.	n.a.	n.a.	n.a.	17	34	6	12	3	9
**Gutierrez et al., 2019 [36]**	n.a.	n.a.	n.a.	n.a.	n.a.	n.a.	n.a.	n.a.	n.a.	n.a.	n.a.	n.a.	n.a.	n.a.	n.a.	n.a.
**Indar et al., 2009 [37]**	n.a.	n.a.	n.a.	n.a.	n.a.	n.a.	n.a.	n.a.	n.a.	n.a.	n.a.	n.a.	n.a.	n.a.	n.a.	n.a.
**Jouvin et al., 2018 [38]**	n.a.	n.a.	n.a.	n.a.	n.a.	n.a.	n.a.	n.a.	n.a.	n.a.	n.a.	n.a.	n.a.	n.a.	n.a.	n.a.
**Kim et al., 2020 [39]**	n.a.	n.a.	n.a.	n.a.	n.a.	n.a.	n.a.	n.a.	n.a.	n.a.	16	8	n.a.	n.a.	n.a.	n.a.
**Kotze et al., 2017 [41]**	n.a.	n.a.	n.a.	n.a.	n.a.	n.a.	n.a.	n.a.	n.a.	n.a.	n.a.	n.a.	3	5	n.a.	n.a.
**Kotze et al., 2018 [40]**	n.a.	n.a.	5	8	n.a.	n.a.	n.a.	n.a.	n.a.	n.a.	n.a.	n.a.	0	3	n.a.	n.a.
**Krane et al., 2013 [42]**	n.a.	n.a.	24	60	n.a.	n.a.	n.a.	n.a.	n.a.	n.a.	8	9	3	9	n.a.	n.a.
**Kunitake et al., 2008 [43]**	n.a.	n.a.	n.a.	n.a.	n.a.	n.a.	n.a.	n.a.	n.a.	n.a.	n.a.	n.a.	n.a.	n.a.	1	2
**Lau et al., 2015 *(1)* * [21]**	n.a.	n.a.	n.a.	n.a.	n.a.	n.a.	n.a.	n.a.	n.a.	n.a.	n.a.	n.a.	n.a.	n.a.	n.a.	n.a.
**Lau et al., 2015 *(2)* * [21]**	n.a.	n.a.	n.a.	n.a.	n.a.	n.a.	n.a.	n.a.	n.a.	n.a.	n.a.	n.a.	n.a.	n.a.	n.a.	n.a.
**Lightner et al., 2019 [44]**	n.a.	n.a.	2	3	n.a.	n.a.	n.a.	n.a.	n.a.	n.a.	n.a.	n.a.	n.a.	n.a.	n.a.	n.a.
**Maeda et al., 2015 [45]**	n.a.	n.a.	n.a.	n.a.	8	27	n.a.	n.a.	n.a.	n.a.	n.a.	n.a.	n.a.	n.a.	n.a.	n.a.
**Mascarenhas et al., 2012 [46]**	n.a.	n.a.	n.a.	n.a.	n.a.	n.a.	n.a.	n.a.	n.a.	n.a.	1	0	n.a.	n.a.	n.a.	n.a.
**Melo-Pinto et al., 2018 [30]**	n.a.	n.a.	n.a.	n.a.	n.a.	n.a.	n.a.	n.a.	n.a.	n.a.	n.a.	n.a.	n.a.	n.a.	n.a.	n.a.
**Monsinjon et al., 2017 [47]**	n.a.	n.a.	n.a.	n.a.	n.a.	n.a.	n.a.	n.a.	n.a.	n.a.	n.a.	n.a.	n.a.	n.a.	n.a.	n.a.
**Morar et al., 2015 [48]**	n.a.	n.a.	n.a.	n.a.	n.a.	n.a.	n.a.	n.a.	n.a.	n.a.	n.a.	n.a.	n.a.	n.a.	n.a.	n.a.

**Myrelid et al., 2014 [49]**	n.a.	n.a.	43	66	n.a.	n.a.	n.a.	n.a.	n.a.	n.a.	13	38	n.a.	n.a.	n.a.	n.a.
**Nasir et al., 2010 [50]**	n.a.	n.a.	n.a.	n.a.	n.a.	n.a.	n.a.	n.a.	n.a.	n.a.	n.a.	n.a.	n.a.	n.a.	n.a.	n.a.
**Nelson et al., 2014 [51]**	5	20	n.a.	n.a.	2	6	n.a.	n.a.	n.a.	n.a.	n.a.	n.a.	n.a.	n.a.	n.a.	n.a.
**Nørgård et al., 2012 [52]**	n.a.	n.a.	n.a.	n.a.	n.a.	n.a.	n.a.	n.a.	n.a.	n.a.	n.a.	n.a.	n.a.	n.a.	n.a.	n.a.
**Nørgård et al., 2013 [53]**	n.a.	n.a.	n.a.	n.a.	n.a.	n.a.	n.a.	n.a.	n.a.	n.a.	n.a.	n.a.	n.a.	n.a.	n.a.	n.a.
**Regadas et al., 2011 [54]**	n.a.	n.a.	n.a.	n.a.	0	10	n.a.	n.a.	0	2	2	41	1	9	n.a.	n.a.
**Rizzo et al., 2011 [55]**	n.a.	n.a.	n.a.	n.a.	n.a.	n.a.	n.a.	n.a.	n.a.	n.a.	2	0	n.a.	n.a.	3	1
**Selvasekar et al., 2007 [56]**	n.a.	n.a.	n.a.	n.a.	n.a.	n.a.	n.a.	n.a.	n.a.	n.a.	n.a.	n.a.	n.a.	n.a.	n.a.	n.a.
**Serradori et al., 2013 [57]**	n.a.	n.a.	n.a.	n.a.	n.a.	n.a.	n.a.	n.a.	n.a.	n.a.	n.a.	n.a.	n.a.	n.a.	n.a.	n.a.
**Shwaartz et al., 2016 [58]**	n.a.	n.a.	n.a.	n.a.	n.a.	n.a.	n.a.	n.a.	n.a.	n.a.	n.a.	n.a.	n.a.	n.a.	n.a.	n.a.
**Syed et al., 2013 [59]**	n.a.	n.a.	n.a.	n.a.	n.a.	n.a.	n.a.	n.a.	2	4	n.a.	n.a.	n.a.	n.a.	7	12
**Tang et al., 2020 [60]**	n.a.	n.a.	**↑33**	**↓61**	**↑15**	**↓24**	9	26	n.a.	n.a.	3	4	3	10	4	14
**Tay et al., 2003 [61]**	n.a.	n.a.	n.a.	n.a.	n.a.	n.a.	n.a.	n.a.	0	3	n.a.	n.a.	n.a.	n.a.	n.a.	n.a.
**Tiberi et al., 2020 [62]**	n.a.	n.a.	n.a.	n.a.	n.a.	n.a.	n.a.	n.a.	n.a.	n.a.	n.a.	n.a.	n.a.	n.a.	n.a.	n.a.
**Uchino et al., 2013 [65]**	n.a.	n.a.	n.a.	n.a.	**↑4**	**↓70**	5	29	n.a.	n.a.	n.a.	n.a.	n.a.	n.a.	n.a.	n.a.
**Uchino et al., 2013 [66]**	n.a.	n.a.	n.a.	n.a.	1	31	0	15	n.a.	n.a.	n.a.	n.a.	n.a.	n.a.	n.a.	n.a.
**Uchino et al., 2015 [64]**	n.a.	n.a.	n.a.	n.a.	2	22	3	10	n.a.	n.a.	n.a.	n.a.	n.a.	n.a.	n.a.	n.a.
**Uchino et al., 2019 [63]**	n.a.	n.a.	n.a.	n.a.	9	11	7	14	n.a.	n.a.	n.a.	n.a.	n.a.	n.a.	n.a.	n.a.
**Ward et al., 2018 [67]**	n.a.	n.a.	n.a.	n.a.	n.a.	n.a.	n.a.	n.a.	n.a.	n.a.	n.a.	n.a.	n.a.	n.a.	n.a.	n.a.
**Waterman et al., 2012 [68]**	n.a.	n.a.	n.a.	n.a.	n.a.	n.a.	n.a.	n.a.	n.a.	n.a.	n.a.	n.a.	n.a.	n.a.	n.a.	n.a.
**White et al., 2012 [69]**	n.a.	n.a.	n.a.	n.a.	n.a.	n.a.	n.a.	n.a.	n.a.	n.a.	n.a.	n.a.	n.a.	n.a.	n.a.	n.a.
**Yamada et al., 2017 *(1)* * [70]**	15	25	n.a.	n.a.	1	3	n.a.	n.a.	n.a.	n.a.	4	5	n.a.	n.a.	2	5

**Yamada et al., 2017** ***(2)*** *** [70]**	6	30	n.a.	n.a.	4	2	n.a.	n.a.	n.a.	n.a.	1	6	n.a.	n.a.	1	2
**Yamamoto et al., 2016 [71]**	n.a.	n.a.	n.a.	n.a.	n.a.	n.a.	n.a.	n.a.	n.a.	n.a.	n.a.	n.a.	n.a.	n.a.	n.a.	n.a.
**Yu et al., 2019 [72]**	n.a.	n.a.	n.a.	n.a.	n.a.	n.a.	n.a.	n.a.	n.a.	n.a.	n.a.	n.a.	n.a.	n.a.	n.a.	n.a.
**Zhu et al., 2020 [73]**	n.a.	n.a.	n.a.	n.a.	n.a.	n.a.	n.a.	n.a.	n.a.	n.a.	n.a.	n.a.	n.a.	n.a.	n.a.	n.a.
**Zittan et al., 2016 [74]**	n.a.	n.a.	n.a.	n.a.	n.a.	n.a.	n.a.	n.a.	n.a.	n.a.	n.a.	n.a.	n.a.	n.a.	n.a.	n.a.

n = Number; n.a. = Not available; Bold marked = Significant outcome (↓ = significantly lower; ↑ = significantly higher);
[*] Gu et al., 2013; Lau et al. and 2015; Yamada et al., 2017 include two discrete data sets each;
[†] ONIPC = Overall postoperative complications; OC-Dm = Overall Clavien-Dindo minor complications;
[‡] S-SSI = Superficial surgical-site infections; D-SSI = Deep or organ space surgical-site infections; FIS = Fistula formation; ILE = Ileus; SBO = Small bowel obstruction; HEM = Hemorrhage;
[§] I = Intervention group (anti-TNF-α drug exposure within 12 weeks prior to intestinal surgery); C = Control group

Table S3.2 30-day other non-surgical-site postoperative complications

**Author and year**	**Non-surgical-site postoperative complications** [†]											
	**OINSSC, n**		**THR, n**		**CV, n**		**PNEU, n**		**UTI, n**		**SEP, n**	
	**I** [§]	**C** [§]	**I** [§]	**C** [§]	**I** [§]	**C** [§]	**I** [§]	**C** [§]	**I** [§]	**C** [§]	**I** [§]	**C** [§]
**Appau et al., 2008 [25]**	n.a.	n.a.	n.a.	n.a.	n.a.	n.a.	n.a.	n.a.	1	0	**↑12**	**↓32**
**Bafford et al., 2013 [26]**	n.a.	n.a.	n.a.	n.a.	0	0	n.a.	n.a.	n.a.	n.a.	n.a.	n.a.
**Bregnbak et al., 2012 [27]**	n.a.	n.a.	0	1	n.a.	n.a.	1	1	0	1	0	1
**Brouquet et al., 2018 [28]**	n.a.	n.a.	n.a.	n.a.	n.a.	n.a.	n.a.	n.a.	n.a.	n.a.	n.a.	n.a.
**Canedo et al., 2010 [29]**	n.a.	n.a.	n.a.	n.a.	n.a.	n.a.	1	3	n.a.	n.a.	n.a.	n.a.
**El-Hussuna et al., 2012 [31]**	n.a.	n.a.	n.a.	n.a.	n.a.	n.a.	n.a.	n.a.	n.a.	n.a.	n.a.	n.a.
**El-Hussuna et al., 2018 [32]**	n.a.	n.a.	n.a.	n.a.	n.a.	n.a.	n.a.	n.a.	n.a.	n.a.	n.a.	n.a.
**Ferrante et al., 2009 [33]**	1	10	n.a.	n.a.	n.a.	n.a.	n.a.	n.a.	1	5	0	3
**Gainsbury et al., 2011 [34]**	n.a.	n.a.	n.a.	n.a.	n.a.	n.a.	n.a.	n.a.	n.a.	n.a.	n.a.	n.a.

**Gu et al., 2013 *(1)* * [35]**	n.a.	n.a.	n.a.	n.a.	n.a.	n.a.	n.a.	n.a.	n.a.	n.a.	n.a.	n.a.
**Gu et al., 2013 *(2)* * [35]**	n.a.	n.a.	n.a.	n.a.	n.a.	n.a.	2	2	2	8	n.a.	n.a.
**Gutierrez et al., 2019 [36]**	n.a.	n.a.	n.a.	n.a.	n.a.	n.a.	n.a.	n.a.	n.a.	n.a.	n.a.	n.a.
**Indar et al., 2009 [37]**	n.a.	n.a.	n.a.	n.a.	n.a.	n.a.	n.a.	n.a.	n.a.	n.a.	n.a.	n.a.
**Jouvin et al., 2018 [38]**	n.a.	n.a.	n.a.	n.a.	n.a.	n.a.	n.a.	n.a.	n.a.	n.a.	n.a.	n.a.
**Kim et al., 2020 [39]**	n.a.	n.a.	n.a.	n.a.	n.a.	n.a.	0	1	n.a.	n.a.	n.a.	n.a.
**Kotze et al., 2017 [41]**	n.a.	n.a.	n.a.	n.a.	n.a.	n.a.	7	3	1	2	n.a.	n.a.
**Kotze et al., 2018 [40]**	n.a.	n.a.	n.a.	n.a.	n.a.	n.a.	0	2	0	1	n.a.	n.a.
**Krane et al., 2013 [42]**	n.a.	n.a.	5	21	0	2	0	1	0	1	n.a.	n.a.
**Kunitake et al., 2008 [43]**	n.a.	n.a.	3	2	0	2	n.a.	n.a.	n.a.	n.a.	n.a.	n.a.
**Lau et al., 2015 *(1)* * [21]**	n.a.	n.a.	n.a.	n.a.	n.a.	n.a.	n.a.	n.a.	n.a.	n.a.	n.a.	n.a.
**Lau et al., 2015 *(2)* * [21]**	n.a.	n.a.	n.a.	n.a.	n.a.	n.a.	n.a.	n.a.	n.a.	n.a.	n.a.	n.a.
**Lightner et al., 2019 [44]**	n.a.	n.a.	n.a.	n.a.	n.a.	n.a.	n.a.	n.a.	n.a.	n.a.	n.a.	n.a.
**Maeda et al., 2015 [45]**	n.a.	n.a.	n.a.	n.a.	n.a.	n.a.	n.a.	n.a.	n.a.	n.a.	n.a.	n.a.
**Mascarenhas et al., 2012 [46]**	n.a.	n.a.	n.a.	n.a.	n.a.	n.a.	n.a.	n.a.	n.a.	n.a.	n.a.	n.a.
**Melo-Pinto et al., 2018 [30]**	n.a.	n.a.	n.a.	n.a.	n.a.	n.a.	n.a.	n.a.	n.a.	n.a.	n.a.	n.a.
**Monsinjon et al., 2017 [47]**	n.a.	n.a.	n.a.	n.a.	n.a.	n.a.	n.a.	n.a.	n.a.	n.a.	n.a.	n.a.
**Morar et al., 2015 [48]**	n.a.	n.a.	n.a.	n.a.	n.a.	n.a.	n.a.	n.a.	n.a.	n.a.	n.a.	n.a.
**Myrelid et al., 2014 [49]**	n.a.	n.a.	n.a.	n.a.	n.a.	n.a.	n.a.	n.a.	n.a.	n.a.	n.a.	n.a.
**Nasir et al., 2010 [50]**	n.a.	n.a.	n.a.	n.a.	3	8	n.a.	n.a.	n.a.	n.a.	n.a.	n.a.
**Nelson et al., 2014 [51]**	2	4	2	5	n.a.	n.a.	n.a.	n.a.	n.a.	n.a.	n.a.	n.a.
**Nørgård et al., 2012 [52]**	n.a.	n.a.	n.a.	n.a.	n.a.	n.a.	n.a.	n.a.	n.a.	n.a.	n.a.	n.a.
**Nørgård et al., 2013 [53]**	n.a.	n.a.	n.a.	n.a.	n.a.	n.a.	n.a.	n.a.	n.a.	n.a.	n.a.	n.a.
**Regadas et al., 2011 [54]**	n.a.	n.a.	n.a.	n.a.	n.a.	n.a.	n.a.	n.a.	n.a.	n.a.	0	2
**Rizzo et al., 2011 [55]**	n.a.	n.a.	1	0	n.a.	n.a.	n.a.	n.a.	n.a.	n.a.	n.a.	n.a.
**Selvasekar et al., 2007 [56]**	n.a.	n.a.	n.a.	n.a.	n.a.	n.a.	n.a.	n.a.	n.a.	n.a.	n.a.	n.a.
**Serradori et al., 2013 [57]**	n.a.	n.a.	n.a.	n.a.	n.a.	n.a.	n.a.	n.a.	n.a.	n.a.	n.a.	n.a.
**Shwaartz et al., 2016 [58]**	3	4	n.a.	n.a.	n.a.	n.a.	n.a.	n.a.	n.a.	n.a.	n.a.	n.a.

**Syed et al., 2013 [59]**	n.a.	n.a.	10	4	1	3	4	8	n.a.	n.a.	11	17
**Tang et al., 2020 [60]**	n.a.	n.a.	n.a.	n.a.	n.a.	n.a.	n.a.	n.a.	n.a.	n.a.	1	4
**Tay et al., 2003 [61]**	n.a.	n.a.	n.a.	n.a.	n.a.	n.a.	n.a.	n.a.	n.a.	n.a.	n.a.	n.a.
**Tiberi et al., 2020 [62]**	n.a.	n.a.	n.a.	n.a.	n.a.	n.a.	n.a.	n.a.	n.a.	n.a.	n.a.	n.a.
**Uchino et al., 2013 [65]**	n.a.	n.a.	n.a.	n.a.	n.a.	n.a.	n.a.	n.a.	n.a.	n.a.	n.a.	n.a.
**Uchino et al., 2013 [66]**	n.a.	n.a.	n.a.	n.a.	n.a.	n.a.	n.a.	n.a.	n.a.	n.a.	n.a.	n.a.
**Uchino et al., 2015 [64]**	n.a.	n.a.	n.a.	n.a.	n.a.	n.a.	n.a.	n.a.	n.a.	n.a.	n.a.	n.a.
**Uchino et al., 2019 [63]**	n.a.	n.a.	n.a.	n.a.	n.a.	n.a.	n.a.	n.a.	n.a.	n.a.	n.a.	n.a.
**Ward et al., 2018 [67]**	n.a.	n.a.	n.a.	n.a.	n.a.	n.a.	n.a.	n.a.	n.a.	n.a.	n.a.	n.a.
**Waterman et al., 2012 [68]**	n.a.	n.a.	n.a.	n.a.	n.a.	n.a.	3	9	6	11	n.a.	n.a.
**White et al., 2012 [69]**	n.a.	n.a.	n.a.	n.a.	n.a.	n.a.	n.a.	n.a.	n.a.	n.a.	n.a.	n.a.
**Yamada et al., 2017 *(1)* * [70]**	n.a.	n.a.	n.a.	n.a.	0	0	1	3	0	0	n.a.	n.a.
**Yamada et al., 2017 *(2)* * [70]**	n.a.	n.a.	n.a.	n.a.	0	0	0	2	1	2	n.a.	n.a.
**Yamamoto et al., 2016 [71]**	n.a.	n.a.	n.a.	n.a.	n.a.	n.a.	n.a.	n.a.	n.a.	n.a.	n.a.	n.a.
**Yu et al., 2019 [72]**	n.a.	n.a.	n.a.	n.a.	n.a.	n.a.	n.a.	n.a.	n.a.	n.a.	n.a.	n.a.
**Zhu et al., 2020 [73]**	n.a.	n.a.	n.a.	n.a.	n.a.	n.a.	n.a.	n.a.	n.a.	n.a.	n.a.	n.a.
**Zittan et al., 2016 [74]**	n.a.	n.a.	1	23	n.a.	n.a.	n.a.	n.a.	n.a.	n.a.	n.a.	n.a.

n = Number; n.a. = Not available; Bold marked = Significant outcome (↓ = significantly lower; ↑ = significantly higher);

* Gu et al., 2013; Lau et al. and 2015; Yamada et al., 2017 include two discrete data sets each;

† OINSSC = Overall infectious non-surgical-site complications; THR = Thrombosis; CV = Cardiovascular complications; PUL = Pulmonary complications; PNEU = Pneumonia; UTI = Urinary tract infection; SEP = Sepsis;

§ I = Intervention group (anti-TNF-α drug exposure within 12 weeks prior to intestinal surgery); C = Control group

Table S4: Random-effects model meta-analysis for 30-day overall postoperative complications in the anti-TNF-α (intervention) and control group. Analysis for publication bias; sensitivity analysis and subgroup analysis.

OPC	OR with 95% CI - RE	Heterogeneity	Egger's test [24]
**Overall**	OR, 1.23; 95% CI, 1.04-1.45; ***p*=0.02 [↑ (I); ↓(C)]**	I^2 = 44%; ***p*=0.006**	*p*=0.204
**Sensitivity analysis (SA)**			
**Excluded study**	**OR with 95% CI - RE**	**Heterogeneity**	
**Bafford et al., 2013 [26]**	OR, 1.24; 95% CI, 1.05-1.47; ***p*=0.01 [↑ (I); ↓(C)]**	I^2 = 45%; ***p*=0.005**	
**Bregnbak et al., 2012 [27]**	OR, 1.23; 95% CI; 1.04-1.46; ***p*=0.02 [↑ (I); ↓(C)]**	I^2 = 46%; ***p*=0.004**	
**Brouquet et al., 2018 [28]**	OR, 1.17; 95% CI; 1.01-1.36; ***p*=0.04 [↑ (I); ↓(C)]**	I^2 = 28%; *p*=0.08	
**El-Hussuna et al., 2012 [31]**	OR, 1.24; 95% CI; 1.05-1.46; ***p*=0.01 [↑ (I); ↓(C)]**	I^2 = 45%; ***p*=0.005**	
**El-Hussuna et al., 2018 [32]**	OR, 1.23; 95% CI; 1.04-1.46; ***p*=0.02 [↑ (I); ↓(C)]**	I^2 = 46%; ***p*=0.004**	
**Gainsbury et al., 2011 [34]**	OR, 1.23; 95% CI; 1.04-1.46; ***p*=0.02 [↑ (I); ↓(C)]**	I^2 = 46%; ***p*=0.004**	
**Gu et al., 2013 *(1)* * [35]**	OR, 1.25; 95% CI; 1.06-1.47; ***p*=0.009 [↑ (I); ↓(C)]**	I^2 = 44%; ***p*=0.007**	
**Gu et al., 2013 *(2)* * [35]**	OR, 1.25; 95% CI; 1.06-1.49; ***p*=0.009 [↑ (I); ↓(C)]**	I^2 = 43%; ***p*=0.009**	
**Gutierrez et al., 2019 [36]**	OR, 1.18; 95% CI; 1.02-1.38; ***p*=0.03 [↑ (I); ↓(C)]**	I^2 = 33%; ***p*=0.04**	
**Indar et al., 2009 [37]**	OR, 1.22; 95% CI; 1.03-1.45; ***p*=0.02 [↑ (I); ↓(C)]**	I^2 = 46%; ***p*=0.004**	
**Jouvin et al., 2018 [38]**	OR, 1.20; 95% CI; 1.02-1.42; ***p*=0.03 [↑ (I); ↓(C)]**	I^2 = 43%; ***p*=0.008**	
**Kim et al., 2020 [39]**	OR, 1.22; 95% CI; 1.03-1.45; ***p*=0.02 [↑ (I); ↓(C)]**	I^2 = 46%; ***p*=0.004**	
**Kotze et al., 2018 [40]**	OR, 1.21; 95% CI; 1.03-1.43; ***p*=0.02 [↑ (I); ↓(C)]**	I^2 = 45%; ***p*=0.005**	
**Krane et al., 2013 [42]**	OR, 1.24; 95% CI; 1.04-1.47; ***p*=0.02 [↑ (I); ↓(C)]**	I^2 = 45%; ***p*=0.005**	
**Kunitake et al., 2008 [43]**	OR, 1.23; 95% CI; 1.04-1.46; ***p*=0.02 [↑ (I); ↓(C)]**	I^2 = 46%; ***p*=0.004**	
**Lau et al., 2015 (1) * [21]**	OR, 1.21; 95% CI; 1.02-1.43; ***p*=0.03 [↑ (I); ↓(C)]**	I^2 = 44%; ***p*=0.006**	
**Lau et al., 2015 (2) * [21]**	OR, 1.22; 95% CI; 1.03-1.45; ***p*=0.02 [↑ (I); ↓(C)]**	I^2 = 46%; ***p*=0.004**	
**Melo-Pinto et al., 2018 [30]**	OR, 1.25; 95% CI; 1.07-1.47; ***p*=0.004 [↑ (I); ↓(C)]**	I^2 = 38%; ***p*=0.02**	
**Monsinjon et al., 2017 [47]**	OR, 1.23; 95% CI; 1.04-1.46; ***p*=0.01 [↑ (I); ↓(C)]**	I^2 = 46%; ***p*=0.004**	
**Myrelid et al., 2014 [49]**	OR, 1.22; 95% CI; 1.03-1.45; ***p*=0.02 [↑ (I); ↓(C)]**	I^2 = 46%; ***p*=0.004**	
**Nasir et al., 2010 [50]**	OR, 1.23; 95% CI; 1.04-1.46; ***p*=0.02 [↑ (I); ↓(C)]**	I^2 = 46%; ***p*=0.004**	
**Regadas et al., 2011 [54]**	OR, 1.24; 95% CI; 1.05-1.46; ***p*=0.01 [↑ (I); ↓(C)]**	I^2 = 44%; ***p*=0.006**	
**Rizzo et al., 2011 [55]**	OR, 1.22; 95% CI; 1.03-1.44; ***p*=0.02 [↑ (I); ↓(C)]**	I^2 = 45%; ***p*=0.005**	
**Tang et al., 2020 [60]**	OR, 1.21; 95% CI; 1.02-1.44; ***p*=0.03 [↑ (I); ↓(C)]**	I^2 = 45%; ***p*=0.005**	
**Tiberi et al., 2020 [62]**	OR, 1.22; 95% CI; 1.03-1.45; ***p*=0.02 [↑ (I); ↓(C)]**	I^2 = 46%; ***p*=0.004**	
**Ward et al., 2018 [67]**	OR, 1.24; 95% CI; 1.04-1.48; ***p*=0.02 [↑ (I); ↓(C)]**	I^2 = 44%; ***p*=0.007**	
**Yamada et al., 2017 (1) * [70]**	OR, 1.26; 95% CI; 1.07-1.49; ***p*=0.006 [↑ (I); ↓(C)]**	I^2 = 42%; ***p*=0.01**	

**Yamada et al., 2017 (2) * [70]**		OR, 1.22; 95% CI; 1.03-1.45; ***p*=0.02 [↑ (I); ↓(C)]**	I^2 = 46%; ***p*=0.004**		
**Yamamoto et al., 2016 [71]**		OR, 1.25; 95% CI; 1.05-1.48; ***p*=0.01 [↑ (I); ↓(C)]**	I^2 = 44%; ***p*=0.006**		
**Yu et al., 2019 [72]**		OR, 1.22; 95% CI; 1.02-1.45; ***p*=0.03 [↑ (I); ↓(C)]**	I^2 = 46%; ***p*=0.004**		
**Subgroup analysis**					
**Subgroups**		**OR with 95% CI - RE**	**TSD**	**SA**	**TSD**
**IBD**	CD	OR, 1.36; 95% CI, 1.04-1.78; ***p*=0.02 [↑ (I); ↓(C)]**	*p*=0.22	CD	*p*=0.08
	UC/IC	OR, 1.03; 95% CI, 0.86-1.22; *p*=0.76		UC/IC	
	IBD	OR, 1.08; 95% CI, 0.79-1.46; *p*=0.64		x	
**Potential risk factors**					
**OA**	>50%	OR, 1.08; 95% CI, 0.91-1.28; *p*=0.37	*p*=0.24	>50%	*p*=0.86
	<50%	OR, 1.05; 95% CI, 0.78-1.42; *p*=0.76		<50%	
	U	OR, 1.41; 95% CI, 1.06-1.88; ***p*=0.02 [↑ (I); ↓(C)]**		x	
**ELEMS**	EL	OR, 1.18; 95% CI, 0.92-1.51; *p*=0.20	*p*=0.95	EL	*p*=0.75
	ELEMS	OR, 1.25; 95% CI, 0.97-1.60; ***p*=0.08**		ELEMS	
	U	OR, 1.20; 95% CI, 0.84-1.73; *p*=0.32		x	
**PRI**	<50%	OR, 1.45; 95% CI, 1.14-1.85; ***p*=0.0003 [↑ (I); ↓(C)]**	*p*=0.19	<50%	*p*=0.12
	>50%	OR, 1.10; 95% CI, 0.85-1.42; *p*=0.47		>50%	
	U	OR, 1.06; 95% CI, 0.78-1.45; *p*=0.70		x	
**CSIM**	>50%	OR, 1.19; 95% CI, 1.01-1.41; ***p*=0.04 [↑ (I); ↓(C)]**	*p*=0.82	>50%	*p*=0.63
	<50%	OR, 1.31; 95% CI, 0.95-1.80; *p*=0.10		<50%	
	U	OR, 1.09; 95% CI, 0.66-1.82; *p*=0.73		x	

* Gu et al., 2013; Lau et al. and 2015; Yamada et al., 2017 include two discrete data sets each;
Bold marked = Significant outcome (↓ = significantly lower; ↑ = significantly higher);
I = Intervention group (anti-TNF-α drug exposure within 12 weeks prior to intestinal surgery); C = Control group;
OR = Odds ratio; CI = Confidence interval; RE = Random-effects model meta-analysis; TSD = Test for subgroup difference;
IBD = Inflammatory bowel disease; CD = Crohn's disease; UC = Ulcerative colitis; IC = Indeterminate colitis; OA = Open surgery or conversion to open surgery; U = Unknown; EL = Elective surgery; ELEMS = Elective surgery and emergency surgery; PRI = Protective ileostomy; CSIM = Concomitant corticosteroid and/ or immunomodulatory drug administration

Table S5: Random-effects model meta-analysis for 30-day overall infectious postoperative complications in the anti-TNF-α (intervention) and control group. Analysis for publication bias; sensitivity analysis and subgroup analysis.

**OIC**	**OR with 95% CI - RE**	**Heterogeneity**	**Egger's test [24]**
**Overall**	OR, 1.16; 95% CI, 0.92-1.45; *p*=0.21	I^2 = 43%; ***p*=0.02**	*p*=0.154
**Sensitivity analysis (SA)**			
**Excluded study**	**OR with 95% CI - RE**	**Heterogeneity**	
**Bregnbak et al., 2012 [27]**	OR, 1.20; 95% CI; 0.96-1.49; *p*=0.21	I^2 = 40%; ***p*=0.03**	
**Ferrante et al., 2009 [33]**	OR, 1.19; 95% CI; 0.95-1.48; *p*=0.13	I^2 = 41%; ***p*=0.03**	
**Gainsbury et al., 2011 [34]**	OR, 1.18; 95% CI; 0.94-1.49; *p*=0.16	I^2 = 44%; ***p*=0.02**	

**Jouvin et al., 2018 [38]**		OR, 1.12; 95% CI; 0.89-1.41; *p*=0.35	I^2 = 42%; ***p*=0.02**		
**Kim et al., 2020 [39]**		OR, 1.20; 95% CI; 0.96-1.50; *p*=0.11	I^2 = 41%; ***p*=0.03**		
**Krane et al., 2013 [42]**		OR, 1.16; 95% CI; 0.91-1.47; *p*=0.24	I^2 = 46%; ***p*=0.01**		
**Kunitake et al., 2008 [43]**		OR, 1.20; 95% CI; 0.95-1.50; *p*=0.12	I^2 = 42%; ***p*=0.02**		
**Lau et al., 2015 (1) * [21]**		OR, 1.12; 95% CI; 0.89-1.41; *p*=0.32	I^2 = 42%; ***p*=0.02**		
**Lau et al., 2015 (2) * [21]**		OR, 1.16; 95% CI; 0.92-1.46; *p*=0.22	I^2 = 46%; ***p*=0.01**		
**Lightner et al., 2019 [44]**		OR, 1.17; 95% CI; 0.92-1.49; *p*=0.20	I^2 = 45%; ***p*=0.01**		
**Myrelid et al., 2014 [49]**		OR, 1.15; 95% CI; 0.90-1.46; *p*=0.26	I^2 = 46%; ***p*=0.01**		
**Nelson et al., 2014 [51]**		OR, 1.16; 95% CI; 0.90-1.46; *p*=0.26	I^2 = 46%; ***p*=0.01**		
**Regadas et al., 2011 [54]**		OR, 1.17; 95% CI; 0.94-1.47; *p*=0.17	I^2 = 43%; ***p*=0.02**		
**Rizzo et al., 2011 [55]**		OR, 1.15; 95% CI; 0.91-1.45; *p*=0.25	I^2 = 46%; ***p*=0.01**		
**Selvasekar et al., 2007 [56]**		OR, 1.12; 95% CI; 0.90-1.40; *p*=0.32	I^2 = 40%; ***p*=0.03**		
**Syed et al., 2013 [59]**		OR, 1.12; 95% CI; 0.88-1.42; *p*=0.36	I^2 = 43%; ***p*=0.02**		
**Tang et al., 2020 [60]**		OR, 1.11; 95% CI; 0.88-1.39; *p*=0.38	I^2 = 39%; ***p*=0.04**		
**Tay et al., 2003 [61]**		OR, 1.15; 95% CI; 0.91-1.45; *p*=0.25	I^2 = 46%; ***p*=0.01**		
**Ward et al., 2018 [67]**		OR, 1.17; 95% CI; 0.92-1.50; *p*=0.20	I^2 = 43%; ***p*=0.02**		
**Yamada et al., 2017 (1) * [70]**		OR, 1.18; 95% CI; 0.93-1.49; *p*=0.17	I^2 = 44%; ***p*=0.02**		
**Yamada et al., 2017 (2) * [70]**		OR, 1.12; 95% CI; 0.89-1.41; *p*=0.32	I^2 = 43%; ***p*=0.02**		
**Yu et al., 2019 [72]**		OR, 1.13; 95% CI; 0.89-1.45; *p*=0.31	I^2 = 46%; ***p*=0.01**		
**Subgroup analysis**					
**Subgroups**		**OR with 95% CI - RE**	**TSD**	**SA**	**TSD**
**IBD**	CD	OR, 1.44; 95% CI, 1.13-1.82; ***p*=0.003 [↑ (I); ↓(C)]**	*p*=0.09	CD	*p*=0.09
	UC/IC	OR, 0.91; 95% CI, 0.57-1.46; *p*=0.69		UC/IC	
	IBD	OR, 0.88; 95% CI, 0.53-1.48; *p*=0.64		x	
**Potential risk factors**					
**OA**	>50%	OR, 1.10; 95% CI, 0.86-1.40; *p*=0.47	*p*=0.38	>50%	*p*=0.44
	<50%	OR, 0.83; 95% CI, 0.43-1.61; *p*=0.58		<50%	
	U	OR, 1.38; 95% CI, 0.94-2.02; *p*=0.102		x	
**ELEMS**	EL	OR, 1.28; 95% CI, 0.77-2.12; *p*=0.34	*p*=0.29	EL	*p*=0.35
	ELEMS	OR, 0.96; 95% CI, 0.70-1.33; *p*=0.81		ELEMS	
	U	OR, 1.45; 95% CI, 0.94-2.22; *p*=0.09		x	
**PRI**	<50%	OR, 1.36; 95% CI, 1.02-1.82; ***p*=0.04 [↑ (I); ↓(C)]**	*p*=0.44	<50%	*p*=0.26
	>50%	OR, 0.99; 95% CI, 0.63-1.58; *p*=0.98		>50%	
	U	OR, 1.05; 95% CI, 0.64-1.71; *p*=0.85		x	
**CSIM**	>50%	OR, 0.94; 95% CI, 0.63-1.39; *p*=0.75	*p*=0.30	>50%	*p*=0.13
	<50%	OR, 1.35; 95% CI, 1.03-1.78; ***p*=0.03**		<50%	
	U	OR, 1.38; 95% CI, 0.74-2.57; *p*=0.31		x	

* Lau et al. and 2015; Yamada et al., 2017 include two discrete data sets each;
Bold marked = Significant outcome (↓ = significantly lower; ↑ = significantly higher);
I = Intervention group (anti-TNF-α drug exposure within 12 weeks prior to intestinal surgery); C = Control group;
OR = Odds ratio; CI = Confidence interval; RE = Random-effects model meta-analysis; TSD = Test for subgroup difference;

IBD = Inflammatory bowel disease; CD = Crohn's disease; UC = Ulcerative colitis; IC = Indeterminate colitis; OA = Open surgery or conversion to open surgery; U = Unknown; EL = Elective surgery; ELEMS = Elective surgery and emergency surgery; PRI = Protective ileostomy; CSIM = Concomitant corticosteroid and/ or immunomodulatory drug administration

Table S6: Fixed-effects model meta-analysis for 30-day overall postoperative Clavien-Dindo major complications in the anti-TNF-α (intervention) and control group. Subgroup analysis.

OC-DMC		OR with 95% CI - FE		Heterogeneity	
**Overall**		OR, 1.13; 95% CI, 0.85-1.50; p=0.40		I^2 = 0%; p=0.91	
**Subgroup analysis**					
**Subgroups**		**OR with 95% CI - FE**	**TSD**	**SA**	**TSD**
**IBD**	CD	OR, 1.16; 95% CI, 0.85-1.58; p=0.35	p=0.69	CD	-
	UC/IC	-		UC/IC	
	IBD	OR, 0.99; 95% CI, 0.50-1.99; p=0.98		x	
**Potential risk factors**					
**OA**	>50%	OR, 1.11; 95% CI, 0.76-1.63; p=0.60	p=0.63	>50%	p=1.0
	<50%	OR, 1.11; 95% CI, 0.72-1.70; p=0.64		<50%	
	U	OR, 2.78; 95% CI, 0.43-17.99; p=0.28		x	
**ELEMS**	EL	OR, 1.02; 95% CI, 0.67-1.56; p=0.91	p=0.58	EL	p=0.65
	ELEMS	OR, 1.17; 95% CI, 0.78-1.76; p=0.44		ELEMS	
	U	OR, 2.06; 95% CI, 0.58-7.32; p=0.26		x	
**PRI**	<50%	OR, 1.15; 95% CI, 0.84-1.57; p=0.39	p=0.86	<50%	p=0.71
	>50%	OR, 0.99; 95% CI, 0.50-1.99; p=0.98		>50%	
	U	OR, 1.62; 95% CI, 0.28-9.27; p=0.59		x	
**CSIM**	>50%	OR, 1.02; 95% CI, 0.71-1.47; p=0.91	p=0.67	>50%	p=0.43
	<50%	OR, 1.30; 95% CI, 0.81-2.07; p=0.27		<50%	
	U	OR, 1.62; 95% CI, 0.28-9.27; p=0.59		x	

OR = Odds ratio; CI = Confidence interval; FE = Fixed-effects model meta-analysis; TSD = Test for subgroup difference;
SA = Sensitivity analysis; IBD = Inflammatory bowel disease; CD = Crohn's disease; UC = Ulcerative colitis; IC = Indeterminate colitis; OA = Open surgery or conversion to open surgery; U = Unknown; EL = Elective surgery; ELEMS = Elective surgery and emergency surgery; PRI = Protective ileostomy; CSIM = Concomitant corticosteroid and/ or immunomodulatory drug administration

Table S7: Fixed-effects model meta-analysis for 30-day postoperative reoperation rate in the anti-TNF-α (intervention) and control group. Analysis for publication bias and subgroup analysis.

Reoperation		OR with 95% CI - FE		Heterogeneity	Egger's test [24]
**Overall**		OR, 1.02; 95% CI, 0.82-1.26; p=0.88		I^2 = 0%; p=0.80	p=0.439
**Subgroup analysis**					
**Subgroups**		**OR with 95% CI - FE**	**TSD**	**SA**	**TSD**
**IBD**	CD	OR, 1.13; 95% CI, 0.84-1.52; p=0.43	p=0.57	CD	p=0.30
	UC/IC	OR, 0.89; 95% CI, 0.64-1.24; p=0.49		UC/IC	
	IBD	OR, 1.07; 95% CI, 0.52-2.20; p=0.8		x	
**Potential risk factors**					

**OA**	>50%	OR, 1.08; 95% CI, 0.83-1.41; *p*=0.56	*p*=0.74	>50%	*p*=0.68
	<50%	OR, 0.93; 95% CI, 0.48-1.82; *p*=0.84		<50%	
	U	OR, 0.90; 95% CI, 0.58-1.37; *p*=0.61		x	
**ELEMS**	EL	OR, 0.84; 95% CI, 0.46-1.56; *p*=0.59	*p*=0.31	EL	*p*=0.24
	ELEMS	OR, 1.31; 95% CI, 0.89-1.93; *p*=0.18		ELEMS	
	U	OR, 0.93; 95% CI, 0.70-1.23; *p*=0.61		x	
**PRI**	<50%	OR, 1.21; 95% CI, 0.83-1.78; *p*=0.32	*p*=0.60	<50%	*p*=0.34
	>50%	OR, 0.95; 95% CI, 0.69-1.31; *p*=0.76		>50%	
	U	OR, 0.97; 95% CI, 0.61-1.53; *p*=0.88		x	
**CSIM**	>50%	OR, 1.16; 95% CI, 0.77-1.74; *p*=0.49	*p*=0.76	>50%	*p*=0.50
	<50%	OR, 0.98; 95% CI, 0.75-1.28; *p*=0.87		<50%	
	U	OR, 0.92; 95% CI, 0.47-1.81; *p*=0.81		x	

OR = Odds ratio; CI = Confidence interval; FE = Fixed-effects model meta-analysis; TSD = Test for subgroup difference; SA = Sensitivity analysis; IBD = Inflammatory bowel disease; CD = Crohn's disease; UC = Ulcerative colitis; IC = Indeterminate colitis; OA = Open surgery or conversion to open surgery; U = Unknown; EL = Elective surgery; ELEMS = Elective surgery and emergency surgery; PRI = Protective ileostomy; CSIM = Concomitant corticosteroid and/ or immunomodulatory drug administration

Table S8: Fixed-effects model meta-analysis for 30-day postoperative mortality in the anti-TNF-α (intervention) and control group. Analysis for publication bias and subgroup analysis.

**Mortality**		**OR with 95% CI - FE**	**Heterogeneity**		**Egger's test [24]**
**Overall**		OR, 0.73; 95% CI, 0.41-1.30; *p*=0.28	I^2 = 30%; *p*=0.14		*p*=0.361
**Subgroup analysis**					
**Subgroups**		**OR with 95% CI - FE**	**TSD**	**SA**	**TSD**
**IBD**	CD	OR, 0.75; 95% CI, 0.36-1.60; *p*=0.46	*p*=0.10	CD	*p*=0.28
	UC/IC	OR, 0.32; 95% CI, 0.08-1.25; *p*=0.10		UC/IC	
	IBD	OR, 2.82; 95% CI, 0.64-12.35; *p*=0.17		x	
**Potential risk factors**					
**OA**	>50%	OR, 0.53; 95% CI, 0.22-1.28; *p*=0.16	*p*=0.38	>50%	*p*=0.17
	<50%	OR, 2.15; 95% CI, 0.36-12.94; *p*=0.40		<50%	
	U	OR, 0.80; 95% CI, 0.34-1.93; *p*=0.62		x	
**ELEMS**	EL	OR, 0.63; 95% CI, 0.16-2.45; *p*=0.51	***p*=0.04**	EL	*p*=0.08
	ELEMS	OR, 3.69; 95% CI, 0.91-14.89; *p*=0.07		ELEMS	
	U	OR, 0.46; 95% CI, 0.20-1.06; *p*=0.07		x	
**PRI**	<50%	OR, 1.54; 95% CI, 0.60-3.92; *p*=0.37	*p*=0.18	<50%	*p*=0.12
	>50%	OR, 0.50; 95% CI, 0.17-1.43; *p*=0.20		>50%	
	U	OR, 0.45; 95% CI, 0.14-1.53; *p*=0.20		x	
**CSIM**	>50%	OR, 2.02; 95% CI, 0.81-5.08; *p*=0.13	***p*=0.02**	>50%	***p*=0.006**
	<50%	OR, 0.32; 95% CI, 0.12-0.83; ***p*=0.02 [↓(I); ↑(C)]**		<50%	
	U	OR, 2.69; 95% CI, 0.17-43.39; *p*=0.49		x	

Bold marked = Significant outcome (↓ = significantly lower; ↑ = significantly higher);
I = Intervention group (anti-TNF-α drug exposure within 12 weeks prior to intestinal surgery); C = Control group;

OR = Odds ratio; CI = Confidence interval; FE = Fixed-effects model meta-analysis; TSD = Test for subgroup difference;
SA = Sensitivity analysis; IBD = Inflammatory bowel disease; CD = Crohn's disease; UC = Ulcerative colitis; IC = Indeterminate colitis; OA = Open surgery or conversion to open surgery; U = Unknown; EL = Elective surgery; ELEMS = Elective surgery and emergency surgery; PRI = Protective ileostomy; CSIM = Concomitant corticosteroid and/ or immunomodulatory drug administration

Table S9: Random-effects model meta-analysis for 30-day overall postoperative infectious surgical-site complications in the anti-TNF-α (intervention) and control group. Analysis for publication bias; sensitivity analysis and subgroup analysis.

OISSC	OR with 95% CI - RE	Heterogeneity	Egger's test [24]
**Overall**	OR, 0.81; 95% CI, 0.45-1.45; p=0.48	I^2 = 76%; $\boldsymbol{p<0.0001}$	p=0.439

**Sensitivity analysis (SA)**		
**Excluded Study**	**OR with 95% CI - RE**	**Heterogeneity**
**Ferrante et al., 2009 [33]**	OR, 0.87; 95% CI, 0.48-1.87; p=0.65	I^2 = 77%; $\boldsymbol{p<0.0001}$
**Kotze et al., 2017 [41]**	OR, 0.72; 95% CI, 0.39-1.34; p=0.60	I^2 = 75%; $\boldsymbol{p<0.0001}$
**Kotze et al., 2018 [40]**	OR, 0.75; 95% CI, 0.41-1.40; p=0.37	I^2 = 77%; $\boldsymbol{p<0.00001}$
**Maeda et al., 2015 [45]**	OR, 0.87; 95% CI, 0.46-1.62; p=0.65	I^2 = 76%; $\boldsymbol{p<0.0001}$
**Nelson et al., 2014 [51]**	OR, 0.79; 95% CI, 0.42-1.47; p=0.45	I^2 = 78%; $\boldsymbol{p<0.00001}$
**Serradori et al., 2013 [57]**	OR, 0.71; 95% CI, 0.39-1.28; p=0.25	I^2 = 74%; $\boldsymbol{p<0.0001}$
**Tang et al., 2020 [60]**	OR, 0.72; 95% CI, 0.40-1.32; p=0.29	I^2 = 71%; $\boldsymbol{p=0.0003}$
**Uchino et al., 2013 [65]**	OR, 0.93; 95% CI, 0.53-1.65; p=0.81	I^2 = 70%; $\boldsymbol{p=0.0005}$
**Uchino et al., 2013 [66]**	OR, 0.89; 95% CI, 0.50-1.59; p=0.70	I^2 = 76%; $\boldsymbol{p<0.0001}$
**Uchino et al., 2015 [64]**	OR, 0.87; 95% CI, 0.47-1.61; p=0.65	I^2 = 77%; $\boldsymbol{p<0.0001}$
**Uchino et al., 2019 [63]**	OR, 0.82; 95% CI, 0.43-1.59; p=0.57	I^2 = 77%; $\boldsymbol{p<0.00001}$

**Subgroup analysis**					
**Subgroups**		**OR with 95% CI - RE**	**TSD**	**SA**	**TSD**
**IBD**	CD	OR, 1.18; 95% CI, 0.53-2.62; p=0.69	p=0.10	CD	-
	UC/IC	OR, 0.52; 95% CI, 0.31-0.89; $\boldsymbol{p=0.02}$ **[↓(I); ↑(C)]**		UC/IC	
	IBD	-		x	
**Potential risk factors**					
**OA**	>50%	OR, 0.64; 95% CI, 0.23-1.76; p=0.39	p=0.64	>50%	p=0.94
	<50%	OR, 0.69; 95% CI, 0.17-2.70; p=0.59		<50%	
	U	OR, 1.23; 95% CI, 0.45-3.35; p=0.69		x	
**ELEMS**	EL	OR, 2.02; 95% CI, 1.31-3.11; $\boldsymbol{p=0.002}$ **[↑(I); ↓(C)]**	$\boldsymbol{p<0.00001}$	EL	$\boldsymbol{p<0.00001}$
	ELEMS	OR, 0.31; 95% CI, 0.18-0.55; $\boldsymbol{p<0.0001}$ **[↓(I); ↑(C)]**		ELEMS	
	U	OR, 0.80; 95% CI, 0.36-1.78; p=0.58		x	
**PRI**	<50%	OR, 1.43; 95% CI, 0.60-3.43; p=0.42	p=0.10	<50%	p=0.31
	>50%	OR, 0.55; 95% CI, 0.11-2.85; p=0.48		>50%	
	U	OR, 0.49; 95% CI, 0.31-0.77; $\boldsymbol{p=0.002}$ **[↓(I); ↑(C)]**		x	
**CSIM**	>50%	OR, 1.60; 95% CI, 0.95-2.72; p=0.08	$\boldsymbol{p=0.007}$	>50%	p=0.27
	<50%	OR, 0.54; 95% CI, 0.08-3.51; p=0.52		<50%	

	U	OR, 0.52; 95% CI, 0.33-0.84; ***p*=0.007 [↓(I); ↑(C)]**		x	
Bold marked = Significant outcome (↓ = significantly lower; ↑ = significantly higher);<br>I = Intervention group (anti-TNF-α drug exposure within 12 weeks prior to intestinal surgery); C = Control group;<br>OR = Odds ratio; CI = Confidence interval; RE = Random-effects model meta-analysis; TSD = Test for subgroup difference;<br>IBD = Inflammatory bowel disease; CD = Crohn's disease; UC = Ulcerative colitis; IC = Indeterminate colitis; OA = Open surgery or conversion to open surgery; U = Unknown; EL = Elective surgery; ELEMS = Elective surgery and emergency surgery; PRI = Protective ileostomy; CSIM = Concomitant corticosteroid and/ or immunomodulatory drug administration					

Table S10: Fixed-effects model meta-analysis for 30-day postoperative intra-abdominal septic complications in the anti-TNF-α (intervention) and control group. Subgroup analysis.

IASC		OR with 95% CI - FE		Heterogeneity	
**Overall**		OR, 1.18; 95% CI, 1.44-2.49; ***p*<0.00001 [↑(I); ↓(C)]**		I^2 = 47%; *p*=0.06	
**Subgroup Analysis**					
**Subgroups**		**OR with 95% CI - FE**	**TSD**	**SA**	**TSD**
**IBD**	CD	OR, 1.92; 95% CI, 1.45-2.53; ***p*<0.00001 [↑(I); ↓(C)]**	*p*=0.47	CD	-
	UC/IC	OR, 0.76; 95% CI, 0.06-9.10; *p*=0.83		UC/IC	
	IBD	-		x	
**Potential risk factors**					
**OA**	>50%	OR, 1.38; 95% CI, 0.92-2.06; *p*=0.12	***p*=0.04**	>50%	*p*=0.65
	<50%	OR, 0.76; 95% CI, 0.06-9.10; *p*=0.83		<50%	
	U	OR, 2.69; 95% CI, 1.85-3.91; ***p*<0.00001 [↑(I); ↓(C)]**		x	
**ELEMS**	EL	OR, 1.95; 95% CI, 0.94-4.05; ***p*=0.07 [↑(I); ↓(C)]**	*p*=0.31	EL	*p*=0.55
	ELEMS	OR, 1.51; 95% CI, 0.99-2.30; *p*=0.06		ELEMS	
	U	OR, 2.38; 95% CI, 1.58-3.59; ***p*<0.0001 [↑(I); ↓(C)]**		x	
**PRI**	<50%	OR, 2.11; 95% CI, 1.49-2.98; ***p*<0.0001 [↑(I); ↓(C)]**	*p*=0.31	<50%	-
	>50%	-		>50%	
	U	OR, 1.57; 95% CI, 1.00-2.47; ***p*=0.05 [↑(I); ↓(C)]**		x	
**CSIM**	>50%	OR, 1.38; 95% CI, 0.33-5.72; *p*=0.66	***p*=0.005**	>50%	*p*=0.93
	<50%	OR, 1.47; 95% CI, 1.04-2.07; ***p*=0.03 [↑(I); ↓(C)]**		<50%	
	U	OR, 4.00; 95% CI, 2.41-6.62; ***p*<0.00001 [↑(I); ↓(C)]**		x	
Bold marked = Significant outcome (↓ = significantly lower; ↑ = significantly higher);<br>I = Intervention group (anti-TNF-α drug exposure within 12 weeks prior to intestinal surgery); C = Control group;<br>OR = Odds ratio; CI = Confidence interval; FE = Fixed-effects model meta-analysis; TSD = Test for subgroup difference; SA = Sensitivity analysis; IBD = Inflammatory bowel disease; CD = Crohn's disease; UC = Ulcerative colitis; IC = Indeterminate colitis; OA = Open surgery or conversion to open surgery; U = Unknown; EL = Elective surgery; ELEMS = Elective surgery and emergency					

surgery; PRI = Protective ileostomy; CSIM = Concomitant corticosteroid and/ or immunomodulatory drug administration

Table S11: Fixed-effects model meta-analysis for 30-day postoperative anastomotic leakage in the anti-TNF-α (intervention) and control group. Analysis for publication bias and subgroup analysis.

AL		OR with 95% CI - FE	Heterogeneity		Egger's test [24]
**Overall**		OR, 1.02; 95% CI, 0.79-1.32; *p*=0.86	I^2 = 0%; *p*=0.88		*p*=0.216
**Subgroup analysis**					
**Subgroups**		**OR with 95% CI - FE**	**TSD**	**SA**	**TSD**
**IBD**	CD	OR, 1.23; 95% CI, 0.86-1.75; *p*=0.26	*p*=0.51	CD	*p*=0.31
	UC/IC	OR, 0.89; 95% CI, 0.53-1.49; *p*=0.65		UC/IC	
	IBD	OR, 0.92; 95% CI, 0.51-1.66; *p*=0.79		x	
**Potential risk factors**					
**OA**	>50%	OR, 1.07; 95% CI, 0.71-1.59; *p*=0.76	*p*=1.0	>50%	*p*=0.96
	<50%	OR, 1.05; 95% CI, 0.66-1.66; *p*=0.83		<50%	
	U	OR, 1.07; 95% CI, 0.64-1.79; *p*=0.80		x	
**ELEMS**	EL	OR, 1.12; 95% CI, 0.57-2.20; *p*=0.75	*p*=0.63	EL	*p*=0.86
	ELEMS	OR, 1.20; 95% CI, 0.81-1.78; *p*=0.36		ELEMS	
	U	OR, 0.91; 95% CI, 0.60-1.38; *p*=0.65		x	
**PRI**	<50%	OR, 1.01; 95% CI, 0.65-1.57; *p*=0.97	*p*=0.83	<50%	*p*=0.92
	>50%	OR, 0.98; 95% CI, 0.59-1.62; *p*=0.92		>50%	
	U	OR, 1.18; 95% CI, 0.74-1.88; *p*=0.47		x	
**CSIM**	>50%	OR, 1.13; 95% CI, 0.78-1.65; *p*=0.51	*p*=0.81	>50%	*p*=0.77
	<50%	OR, 1.04; 95% CI, 0.69-1.57; *p*=0.84		<50%	
	U	OR, 0.84; 95% CI, 0.37-1.91; *p*=0.68		x	

Bold marked = Significant outcome (↓ = significantly lower; ↑ = significantly higher);
I = Intervention group (anti-TNF-α drug exposure within 12 weeks prior to intestinal surgery); C = Control group;
OR = Odds ratio; CI = Confidence interval; FE = Fixed-effects model meta-analysis; TSD = Test for subgroup difference;
SA = Sensitivity analysis; IBD = Inflammatory bowel disease; CD = Crohn's disease; UC = Ulcerative colitis; IC = Indeterminate colitis; OA = Open surgery or conversion to open surgery; U = Unknown; EL = Elective surgery; ELEMS = Elective surgery and emergency surgery; PRI = Protective ileostomy; CSIM = Concomitant corticosteroid and/ or immunomodulatory drug administration

Table S12: Fixed-effects model meta-analysis for 30-day postoperative superficial surgical-site infections in the anti-TNF-α (intervention) and control group. Analysis for publication bias; sensitivity analysis and subgroup analysis

Superficial SSI	OR with 95% CI - RE	Heterogeneity	Egger's test [24]
**Overall**	OR, 0.67; 95% CI, 0.32-1.40; *p*=0.29	I^2 = 69%; ***p*=0.0004**	*p*=0.643
**Sensitivity analysis (SA)**			

Excluded study	OR with 95% CI - RE	Heterogeneity
**El-Hussuna et al., 2018 [32]**	OR, 0.66; 95% CI, 0.30-1.45; *p*=0.30	I^2 = 72%; ***p*=0.0002**
**Maeda et al., 2015 [45]**	OR, 0.70 95% CI, 0.30-1.64; *p*=0.42	I^2 = 70%; ***p*=0.0004**
**Nelson et al., 2014 [51]**	OR, 0.67; 95% CI, 0.30-1.49; *p*=0.32	I^2 = 72%; ***p*=0.0002**
**Regadas et al., 2011 [54]**	OR, 0.69; 95% CI, 0.32-1.49; *p*=0.34	I^2 = 72%; ***p*=0.0004**
**Tang et al., 2020 [60]**	OR, 0.54; 95% CI, 0.28-1.03; *p*=0.06	I^2 = 47%; ***p*=0.05**
**Uchino et al., 2013 [65]**	OR, 0.80; 95% CI, 0.39-1.66; *p*=0.55	I^2 = 62%; ***p*=0.005**
**Uchino et al., 2013 [66]**	OR, 0.73; 95% CI, 0.34-1.57; *p*=0.42	I^2 = 70%; ***p*=0.0004**
**Uchino et al., 2015 [64]**	OR, 0.74; 95% CI, 0.34-1.62; *p*=0.45	I^2 = 70%; ***p*=0.0005**
**Uchino et al., 2019 [63]**	OR, 0.64; 95% CI, 0.27-1.52; *p*=0.31	I^2 = 72%; ***p*=0.0002**
**Yamada et al., 2017 (1) * [70]**	OR, 0.69; 95% CI, 0.32-1.51; *p*=0.35	I^2 = 72%; ***p*=0.0002**
**Yamada et al., 2017 (2) * [70]**	OR, 0.54; 95% CI, 0.27-1.09; *p*=0.09	I^2 = 63%; ***p*=0.004**

**Subgroup analysis**					
**Subgroups**		**OR with 95% CI - RE**	**TSD**	**SA**	**TSD**
**IBD**	CD	OR, 0.58; 95% CI, 0.15-2.26; *p*=0.43	*p*=0.93	CD	*p*=0.74
	UC/IC	OR, 0.78; 95% CI, 0.25-2.44; *p*=0.67		UC/IC	
	IBD	OR, 0.55; 95% CI, 0.08-3.58; *p*=0.53		x	

*Yamada et al., 2017 includes two discrete data sets;
I = Intervention group (anti-TNF-α drug exposure within 12 weeks prior to intestinal surgery); C = Control group;
OR = Odds ratio; CI = Confidence interval; RE = Random-effects model meta-analysis; TSD = Test for subgroup difference;
IBD = Inflammatory bowel disease; CD = Crohn's disease; UC = Ulcerative colitis; IC = Indeterminate colitis; OA = Open surgery or conversion to open surgery; U = Unknown; EL = Elective surgery; ELEMS = Elective surgery and emergency surgery; PRI = Protective ileostomy; CSIM = Concomitant corticosteroid and/ or immunomodulatory drug administration

Table S13: Fixed-effects model meta-analysis for 30-day postoperative ileus in the anti-TNF-α (intervention) and control group. Analysis for publication bias and subgroup analysis.

Ileus	OR with 95% CI - FE	Heterogeneity	Egger's test [24]
**Overall**	OR, 1.11; 95% CI, 0.81-1.51; *p*=0.52	I^2 = 32%; *p*=0.15	*p*=0.115

**Subgroup analysis**					
**Subgroups**		**OR with 95% CI - FE**	**TSD**	**SA**	**TSD**
**IBD**	CD	OR, 0.94; 95% CI, 0.55-1.61; *p*=0.82	*p*=0.76	CD	*p*=0.50
	UC/IC	OR, 1.20; 95% CI, 0.76-1.89; *p*=0.44		UC/IC	
	IBD	OR, 1.23; 95% CI, 0.61-2.47; *p*=0.56		x	

OR = Odds ratio; CI = Confidence interval; FE = Fixed-effects model meta-analysis; TSD = Test for subgroup difference;

SA = Sensitivity analysis; IBD = Inflammatory bowel disease; CD = Crohn's disease; UC = Ulcerative colitis; IC = Indeterminate colitis

Table S14: Fixed-effects model meta-analysis for 30-day postoperative small bowel obstruction in the anti-TNF-α (intervention) and control group. Analysis for publication bias and subgroup analysis.

**SBO**		**OR with 95% CI - FE**		**Heterogeneity**	
**Overall**		OR, 0.76; 95% CI, 0.43-1.32; *p*=0.33		I^2 = 0%; *p*=0.86	
**Subgroup analysis**					
**Subgroups**		**OR with 95% CI - FE**	**TSD**	**SA**	**TSD**
**IBD**	CD	OR, 0.55; 95% CI, 0.22-1.39; *p*=0.21	*p*=0.69	CD	*p*=0.41
	UC/IC	OR, 0.95; 95% CI, 0.38-2.36; *p*=0.91		UC/IC	
	IBD	OR, 0.88; 95% CI, 0.29-2.69; *p*=0.82		x	
OR = Odds ratio; CI = Confidence interval; FE = Fixed-effects model meta-analysis; TSD = Test for subgroup difference;<br>SA = Sensitivity analysis; IBD = Inflammatory bowel disease; CD = Crohn's disease; UC = Ulcerative colitis; IC = Indeterminate colitis					

Table S15: Fixed-effects model meta-analysis for 30-day postoperative hemorrhage in the anti-TNF-α (intervention) and control group. Subgroup analysis.

**Hemorrhage**		**OR with 95% CI - FE**		**Heterogeneity**	
**Overall**		OR, 0.83; 95% CI, 0.49-1.41; *p*=0.50		I^2 = 0%; *p*=0.86	
**Subgroup analysis**					
**Subgroups**		**OR with 95% CI - FE**	**TSD**	**SA**	**TSD**
**IBD**	CD	OR, 0.73; 95% CI, 0.37-1.43; *p*=0.36	*p*=0.37	CD	*p*=1.0
	UC/IC	OR, 0.73; 95% CI, 0.25-2.12; *p*=0.56		UC/IC	
	IBD	OR, 2.47; 95% CI, 0.50-12.29; *p*=0.27		x	
OR = Odds ratio; CI = Confidence interval; FE = Fixed-effects model meta-analysis; TSD = Test for subgroup difference;<br>SA = Sensitivity analysis; IBD = Inflammatory bowel disease; CD = Crohn's disease; UC = Ulcerative colitis; IC = Indeterminate colitis					

Table S16: Fixed-effects model meta-analysis for 30-day postoperative thrombosis in the anti-TNF-α (intervention) and control group. Subgroup analysis.

**Thrombosis**		**OR with 95% CI - FE**		**Heterogeneity**	
**Overall**		OR, 1.30; 95% CI, 0.75-2.25; *p*=0.34		I^2 = 14%; *p*=0.32	
**Subgroup analysis**					
**Subgroups**		**OR with 95% CI - FE**	**TSD**	**SA**	**TSD**
**IBD**	CD	OR, 3.05; 95% CI, 0.94-9.95; *p*=0.06	*p*=0.24	CD	*p*=0.14
	UC/IC	OR, 0.85; 95% CI, 0.25-2.87; *p*=0.79		UC/IC	
	IBD	OR, 1.03; 95% CI, 0.48-2.24; *p*=0.94		x	
OR = Odds ratio; CI = Confidence interval; FE = Fixed-effects model meta-analysis; TSD = Test for subgroup difference;<br>SA = Sensitivity analysis; IBD = Inflammatory bowel disease; CD = Crohn's disease; UC = Ulcerative colitis; IC = Indeterminate colitis					

Table S17: Fixed-effects model meta-analysis for 30-day postoperative pneumonia in the anti-TNF-α (intervention) and control group. Subgroup analysis.

**Thrombosis**		**OR with 95% CI - FE**		**Heterogeneity**	
**Overall**		OR, 0.82; 95% CI, 0.44-1.54; *p*=0.53		I^2 = 0%; *p*=0.79	
**Subgroup analysis**					
**Subgroups**		**OR with 95% CI - FE**	**TSD**	**SA**	**TSD**
**IBD**	CD	OR, 0.74; 95% CI, 0.36-1.53; *p*=0.42	*p*=0.85	CD	*p*=0.58
	UC/IC	OR, 1.11; 95% CI, 0.32-3.86; *p*=0.86		UC/IC	
	IBD	OR, 0.89; 95% CI, 0.26-3.02; *p*=0.85		x	
OR = Odds ratio; CI = Confidence interval; FE = Fixed-effects model meta-analysis; TSD = Test for subgroup difference; SA = Sensitivity analysis; IBD = Inflammatory bowel disease; CD = Crohn's disease; UC = Ulcerative colitis; IC = Indeterminate colitis					

Table S18: Fixed-effects model meta-analysis for 30-day postoperative urinary tract infections in the anti-TNF-α (intervention) and control group. Subgroup analysis.

**UTI**		**OR with 95% CI - FE**		**Heterogeneity**	
**Overall**		OR, 1.02; 95% CI, 0.54-1.91; *p*=0.96		I^2 = 0%; *p*=0.65	
**Subgroup analysis**					
**Subgroups**		**OR with 95% CI - FE**	**TSD**	**SA**	**TSD**
**IBD**	CD	OR, 0.98; 95% CI, 0.24-4.02; *p*=0.97	*p*=0.68	CD	*p*=0.76
	UC/IC	OR, 0.74; 95% CI, 0.26-2.13; *p*=0.58		UC/IC	
	IBD	OR, 1.41; 95% CI, 0.53-3.72; *p*=0.49		x	
OR = Odds ratio; CI = Confidence interval; FE = Fixed-effects model meta-analysis; TSD = Test for subgroup difference; SA = Sensitivity analysis; IBD = Inflammatory bowel disease; CD = Crohn's disease; UC = Ulcerative colitis; IC = Indeterminate colitis					

Table S19: Fixed-effects model meta-analysis for 30-day postoperative sepsis in the anti-TNF-α (intervention) and control group. Subgroup analysis.

**Sepsis**		**OR with 95% CI - FE**		**Heterogeneity**	
**Overall**		OR, 1.24; 95% CI, 0.75-2.04; *p*=0.41		I^2 = 0%; *p*=0.44	
**Subgroup analysis**					
**Subgroups**		**OR with 95% CI - FE**	**TSD**	**SA**	**TSD**
**IBD**	CD	OR, 1.26; 95% CI, 0.75-2.13; *p*=0.38	*p*=0.90	CD	*p*=0.67
	UC/IC	OR, 0.77; 95% CI, 0.09-6.98; *p*=0.82		UC/IC	
	IBD	OR, 1.54; 95% CI, 0.07-32.90; *p*=0.78		x	
OR = Odds ratio; CI = Confidence interval; FE = Fixed-effects model meta-analysis; TSD = Test for subgroup difference; SA = Sensitivity analysis; IBD = Inflammatory bowel disease; CD = Crohn's disease; UC = Ulcerative colitis; IC = Indeterminate colitis					
